# The potential of high temporal resolution automatic measurements of PM_2.5_ composition as an alternative to the filter-based manual method used in routine monitoring

**DOI:** 10.1016/j.atmosenv.2023.120148

**Published:** 2023-12-15

**Authors:** Marsailidh M. Twigg, Chiara F. Di Marco, Elizabeth A. McGhee, Christine F. Braban, Eiko Nemitz, Richard J.C. Brown, Kevin C. Blakley, Sarah R. Leeson, Agnieszka Sanocka, David C. Green, Max Priestman, Veronique Riffault, Aude Bourin, Maria Cruz Minguillón, Marta Via, Jurgita Ovadnevaite, Darius Ceburnis, Colin O’Dowd, Laurent Poulain, Bastian Stieger, Ulla Makkonen, Ian C. Rumsey, Gregory Beachley, John T. Walker, David M. Butterfield

**Affiliations:** a UK Centre for Ecology & Hydrology, Bush Estate, Penicuik, EH26 0QB, UK; b National Physical Laboratory, Hampton Road, Teddington, London, TW11 0LW, UK; c Ricardo Energy & Environment, Wantage, England, UK; d MRC Centre for Environment and Health, Environmental Research Group, Imperial College London, UK; e IMT Nord Europe, Institut Mines-Télécom, Univ. Lille, Centre for Energy and Environment, F-59000 Lille, France; f Institute of Environmental Assessment and Water Research (IDAEA-CSIC), Barcelona, 08034, Spain; g The Ryan Institute’s Centre for Climate and Air Pollution Studies, School of Natural Sciences, University of Galway, Galway, H91 CF50, Ireland; h Atmospheric Chemistry Department (ACD), Leibniz Institute for Tropospheric Research (TROPOS), Permoserstr. 15, 04318 Leipzig, Germany; i Finnish Meteorological Institute, 00560, Helsinki, Finland; j Office of Research and Development, US Environmental Protection Agency, Research Triangle Park, NC 27711, USA; k Office of Atmospheric Programs, US Environmental Protection Agency, Washington, DC 20460, USA

**Keywords:** PM_2.5,_ inorganic aerosol, ACSM, HR-TOF-AMS, MARGA, AIM

## Abstract

Under the EU Air Quality Directive (AQD) 2008/50/EC member states are required to undertake routine monitoring of PM_2.5_ composition at background stations. The AQD states for PM_2.5_ speciation this should include at least: nitrate $\left({\text{NO}}_{3}^{-}\right)$, sulfate $\left({\text{SO}}_{4}^{2-}\right)$, chloride (Cl^−^), ammonium (NH4^+^), sodium (Na^+^), potassium (K^+^), magnesium (Mg^2+^), calcium (Ca^2+^), elemental carbon (EC) and organic carbon (OC). Until 2017, it was the responsibility of each country to determine the methodology used to report the composition for the inorganic components of PM_2.5_. In August 2017 a European standard method of measurement of PM_2.5_ inorganic chemical components (${\text{NO}}_{3}^{-},\;{\text{SO}}_{4}^{2-}$, Cl^−^, ${\text{NH}}_{4}^{+}$, Na^+^, K^+^, Mg^2+^, Ca^2+^) as deposited on filters (EN16913:2017) was published. From August 2019 this then became the European standard method. This filter method is labour-intensive and provides limited time resolution and is prone to losses of volatile compounds. There is therefore increasing interest in the use of alternative automated methods. For example, the UK reports hourly PM_2.5_ chemical composition using the Monitor for AeRosols and Gases in Ambient air (MARGA, Metrohm, NL). This study is a pre-assessment review of available data to demonstrate if or to what extent equivalence is possible using either the MARGA or other available automatic methods, including the Aerosol Chemical Speciation Monitor (ACSM, Aerodyne Research Inc. US) and the Ambient Ion Monitor (AIM, URG, US).

To demonstrate equivalence three objectives were to be met. The first two objectives focused on data capture and were met by all three instruments. The third objective was to have less than a 50% expanded uncertainty compared to the reference method for each species. Analysis of this objective was carried out using existing paired datasets available from different regions around the world. It was found that the MARGA (2006–2019 model) had the potential to demonstrate equivalence for all species in the standard, though it was only through a combination of case studies that it passed uncertainty criteria. The ACSM has the potential to demonstrate equivalence for ${\text{NH}}_{4}^{+}$, ${\text{SO}}_{4}^{2-}$ and in some conditions ${\text{NO}}_{3}^{-}$, but did not for Cl^−^ due to its inability to quantify refractory aerosol such as sea salt. The AIM has the potential for ${\text{NH}}_{4}^{+}$, ${\text{NO}}_{3}^{-}$, ${\text{SO}}_{4}^{2-}$, Cl^−^ and Mg^2+^. Future investigations are required to determine if the AIM could be optimised to meet the expanded uncertainty criterion for Na^+^, K^+^ and Ca^2+^.

The recommendation is that a second stage to demonstrate equivalence is required which would include both laboratory and field studies of the three candidate methods and any other technologies identified with the potential to report the required species.

## 1. Introduction

Particulate matter of 2.5 μm (PM_2.5_) in aerodynamic diameter or less is of concern to human health. Epidemiological studies have so far been unable to demonstrate if it is chronic exposure to total PM or individual compounds contained within PM_2.5_, which are detrimental to health, and to establish different toxicities for different aerosols. As such the World Health Organisation (WHO) concluded in the Review of Health Aspects of Air Pollution (REVIHAAP) (WHO Regional Office for Europe, 2013) that any long term exposure to PM_2.5_ is a threat to human health and encourages nations to reduce PM exposure.

In Europe, the revised EU Air Quality Directive (AQD) 2008/50/EC^2^ on ambient air quality and cleaner air in Europe specifies that member states are required to carry out measurements of PM_2.5_ total mass and concentrations of appropriate compounds to characterise its chemical composition. The AQD states for PM_2.5_ speciation this should include at least: nitrate (${\text{NO}}_{3}^{-}$), sulfate (${\text{SO}}_{4}^{2-}$), chloride (Cl^−^), ammonium (${\text{NH}}_{4}^{+}$), sodium (Na^+^), potassium (K^+^), magnesium (Mg^2+^), calcium (Ca^2+^), elemental carbon (EC) and organic carbon (OC). The AQD requires measurements to be carried out at rural background sites to better understand the impacts and sources of pollutants in order to develop appropriate policies. Member states are also required, where possible, to co-ordinate measurements with those of the cooperative programme for monitoring and evaluation of the long-range transmission of air pollutants in Europe (EMEP) which was set-up under the 1979 UNECE Convention on Long-range Transboundary Air Pollution (CLRTAP) (Tørseth et al., 2012). At the time when this requirement was introduced into the revised AQD, there was no standard method defined to characterise the chemical composition of PM_2.5_ and as a result each country determined how this requirement of the AQD would be addressed.

The UK had already established two EMEP Supersites (Level II/III) prior to the AQD being transposed into UK law. For Level II sites, the EMEP Monitoring Strategy requests artefact-free methods to distinguish between the gas and aerosol phase of ammonia (${\text{NH}}_{3}{\text{/NH}}_{4}^{+}$) and nitric acid (${\text{HNO}}_{3}{\text{/NO}}_{3}^{-}$) compounds. This is not possible with the simple filter sampler of the reference method (RM) and is typically achieved through 24-h samplers consisting of denuder-filter-pack sampling trains (EMEP/CCC, 2014). These are labour intensive to operate and the daily time-resolution does not provide any information on diel cycles. Instead, the UK chose to adopt the Monitor for AeRosols and Gases in Ambient air (MARGA, Metrohm, NL) system. The dual channel MARGA system deployed in the UK simultaneously provides hourly data on water-soluble inorganic speciated PM_10_ and PM_2.5_ (${\text{NH}}_{4}^{+}$, Na^+^, K^+^, Ca^2+^, Mg^2+^, Cl^−^, ${\text{NO}}_{3}^{-}$ and ${\text{SO}}_{4}^{2-}$), as well as the gases ammonia (NH_3_), nitric acid (HNO_3_), nitrous acid (HONO), sulphur dioxide (SO_2_) and hydrochloric acid (HCl) in one single instrument (Twigg et al., 2015).

In August 2017, however, a standard method of measurement of ${\text{NO}}_{3}^{-}$, ${\text{SO}}_{4}^{2-}$, Cl^−^, ${\text{NH}}_{4}^{+}$, Na^+^, K^+^, Mg^2+^, Ca^2+^ in PM_2.5_ as deposited on filters (EN16913:2017) was published (CEN/TC 264, 2017). From August 2019 this then became the reference method. The new standard requires sampling for 24 h onto filters using the sampling protocol that is laid out in the EN12341:2014 standard for measuring total PM_10_ and PM_2.5_ mass. The EN16913:2017 standard describes how these samples are to be stored and analysed off line by ion chromatography in order to determine PM_2.5_ speciation of inorganic ions. The cations (excluding ${\text{NH}}_{4}^{+}$) can also alternatively be analysed by inductively coupled plasma optical emission spectrometery (ICP-OES) and the ${\text{NH}}_{4}^{+}$ analysed alternatively by photometry or conductometry. The EN16913:2017 standard acknowledges that the method can be subject to losses due to sample evaporation of volatile species. It states that for ${\text{NO}}_{3}^{-}$, ${\text{NH}}_{4}^{+}$ and Cl^−^, there could be an underestimation of up to 30% due to evaporational losses of ammonium nitrate and chloride (NH_4_NO_3_, NH_4_Cl) during filter sampling.

There are however alternative automatic methods (sampling and analysis online), which report all or some of the PM_2.5_ species required by both the AQD and EMEP. These methods include the MARGA, (Metrohm, NL) (Makkonen et al., 2012; Rumsey et al., 2014; Stieger et al., 2018; Twigg et al., 2015), the Aerosol Ion monitor (AIM, URG, ThermoFisher, US) (Beccaceci et al., 2015; Markovic et al., 2012) and the Aerosol Chemical Speciation Monitor (ACSM, Aerodyne Research Inc., US) (Crenn et al., 2015; Crenn et al., 2015; Freney et al., 2019; Poulain et al., 2020; Via et al., 2021), which are increasingly being used for routine monitoring around the world.

Ideally all equipment used in reporting should follow the RM or is able to demonstrate equivalence using an alternative method as described under the Guide to the Demonstration of Equivalence of Ambient Air Monitoring Methods (GDE, 2010). To carry out an equivalence study there are four phases (refer to Table S1 for further details), the first phase being a non experimental pre-assessment to check whether the candidate methods (CM) have the potential for fulfilling the data quality objectives in the directive on the data capture, as well as the measurement uncertainty, which is set by this study (GDE, 2010). This study represents this first phase to provide evidence that the automatic methods (MARGA, AIM, ACSM/HR-TOF-AMS) used in routine monitoring should be considered for future equivalence studies for the EN16913:2017 standard. It is however noted that EN16913:2017 is only recently published and there is currently no requirement yet to implement the standard under the AQD.

## 2. Methods

### 2.1. Description of candidate methods (CMs)

In this study a total of three methods are proposed as CMs for the EN12341:2014. The first two (MARGA and AIM) utilise the same analytical (ion chromatography) principle used in the RM, but are coupled to a system for real-time automated sample collection and analysis. Both wet chemistry systems scrub the gas phase from the sample first and there is no possibility of aerosol volatilisation once in the liquid phase, thus minimising positive and negative sampling artefacts, respectively.

The MARGA (Metrohm, NL) measures simultaneously water soluble aerosols (${\text{NH}}_{4}^{+}$, Na^+^, K^+^, Ca^2+^, Mg^2+^, Cl^−^, ${\text{NO}}_{3}^{-}$ and ${\text{SO}}_{4}^{2-}$) and trace gases (NH_3_, HNO_3_, HONO, SO_2_ and HCl) at hourly resolution. The instrument first captures gases in a wet rotating denuder (Keuken et al., 1967) (WRD) and then water soluble aerosols with a steam jet aerosol collector (Khlystov et al., 1995) (SJAC) reporting concentrations with hourly resolution. Currently there are two versions of the MARGA, the original MARGA (available commercially 2006 to 2019) and the 2060 MARGA (available commercially from 2019). For the purpose of this study only the original MARGA design is assessed, as no data was available on the performance of the MARGA 2060. In the typical configuration, the system measures the following constituents of particulate matter outlined in the EN16913:2017 standard: ${\text{NH}}_{4}^{+}$, Na^+^, K^+^, Ca^2+^, Mg^2+^, Cl^−^, ${\text{NO}}_{3}^{-}$ and ${\text{SO}}_{4}^{2-}$. The PM size cut off is determined by the inlet type and flow rate chosen by the operators and therefore varies between case studies. Table 1 reports the set-ups used in each case. A full description of the method and quality assurance/quality control (QA/QC) protocol used in the UK can be found in Twigg et al. (2015) and detection limits are listed in Supplementary Information, Table S2.

The Ambient Ion Monitor (AIM) 9000-B used in this study provides hourly resolution of particulate anion (Cl^−^, ${\text{NO}}_{3}^{-}$ and ${\text{SO}}_{4}^{2-}$) and cation (Na^+^, ${\text{NH}}_{4}^{+}$, K^+^, Mg^2+^ and Ca^2+^) concentrations (Markovic et al., 2012). To sample, the ambient air is drawn through a membrane-style Liquid Diffusion Denuder where interfering acidic and basic gases are removed. Aerosol collection is similar to that in the MARGA: in order to achieve high collection efficiencies, the particle-laden air stream next enters the Aerosol Super Saturation Chamber to enhance particle growth. An Inertial Particle Separator collects these enlarged particles, which it then stores in an Aerosol Sample Collector until the particles can be injected into the two ion chromatography systems. A full description of the method and quality assurance (QA) that was used in the UK can be found in Beccaceci et al. (2015), with detection limits in the Supplementary Information (Table S3). An alternative model of the AIM (9000-C; not used here) additionally offers analysis of the gases collected by the denuder.

The third method utilises mass spectrometry for analysis, which does not rely on water solublity of the target compounds. Instead, it provides real-time measurements of the chemical composition of submicron non-refactory species that volatilise at a temperature of ~600 °C (Canagaratna et al., 2007; DeCarlo et al., 2006). The emerging gases are subsequently ionised by 70 eV electron impact ionisation and detected using a quadrupole or time-of-flight mass spectrometer. Both the High-Resolution Time-of-Flight Aerosol Mass Spectrometer (HR-TO-F-AMS) and the Aerosol Chemical Speciation Monitor (ACSM) (both Aerodyne Research Inc., US) operate on this principle. The ACSM is a modification of the AMS which is smaller, at lower cost, easier to analyse and ideal for monitoring purposes, whilst the HR-TOF-AMS additionally provides size information. Further details of the HR-TOF-AMS and ACSM can be found in DeCarlo et al. (2006) and Ng et al. (2011) respectively and the detection limits in Table S4. HR-TOF-AMS and ACSM have traditionally been operated with the so-called standard lens (transfer inlet into the vacuum system) which approximates PM_1_; no paired data were available for instruments using the newer PM_2.5_ lens (Peck et al., 2016).

### 2.2. Test datasets

All three automatic methods of interest are or have been used in the UK’s air quality monitoring networks. To determine if the first two data quality objectives are met, data was obtained from the UK-Air website for the MARGA (PM_2.5_ from the Auchencorth Moss (Twigg et al., 2015) and Chilbolton (Walker et al., 2019) field sites) and AIM (PM_10_, North Kensington (Beccaceci et al., 2015)) for three calendar years (2016–2018). ACSM data from North Kensington (Crenn et al., 2015; Freney et al., 2019) using a PM_1_ lens for 2 years (2016–2017) was obtained directly from Imperial College London (*David Green, personal communication)*.

To determine if equivalence was possible, paired datasets were obtained from other studies, which had used the proposed CMs compared to filter samples. In total eight case studies were obtained: 1. Revin, France, 2. Barcelona, Spain, 3. Mace Head, Ireland, 4. Melpitz, Germany, 5. Kumpula, Finland, 6. Research Triangle Park, US, 7. San Pietro Capofiume, Italy and 8. North Kensington, UK. Details of the set-up and sampling period of each paired dataset are summarised in Table 1, including references to each dataset.

### 2.3. Calculation of equivalence

Equivalence is defined under the Terms of Reference for the CEN/TC 264 Ambient Air standards (GDE, 2010). It states that methods other than the RM may be used for implementation of the Directive provided they fulfil the minimum data quality objectives specified in the Directive. Therefore, in this study the priority is to determine if the two air quality objectives of the Directive (2008/50/EC) are met by the CMs. The objectives in Appendix IV of the directive for speciated PM_2.5_ are:

1. Minimum data coverage = 14% (which equates to 8 weeks over 1 calendar year)
2. Minimum data coverage over a 24 h period = 90% (>21.6 h)

In addition, a third data quality objective was set in this study for equivalence to the RM, not currently in the AQD of:

- Expanded uncertainty has to be less than 50 %.

Here, the expanded uncertainty (W_cm_) was studied with the methodology set out by CEN/TC 264/WG15, using the tool currently adopted to demonstrate equivalence for total PM monitors (Equivalence, 2020), but is generic enough to be transferrable to other similar PM-based assessments. This compares RMs and CMs in an orthogonal regression analysis to calculate the W_cm._ If either the slope is found to be significantly different from one and/or the intercept is significantly different from zero in the orthogonal regression, the CM can be calibrated (corrected) using the values obtained in the regression. An orthogonal regression with the corrected CM is then undertaken to determine the W_cm_. Further details of the methodology can be found in the Guidance for the Demonstration of Equivalence of Ambient Air Monitoring Methods (GDE, 2010) and the tool can be downloaded at https://ec.europa.eu/environment/air/quality/assessment.htm. (Refer to Supplementary Material for further details on the calculation of W_cm_).

The following criteria for the W_cm_ analysis have to be met:

Criterion 1: The slope (uncorrected or corrected) is not significantly different from one.
Criterion 2: The intercept (uncorrected or corrected) is not significantly different from zero.
Criterion 3: The expanded uncertainty is less than 50%.

## 3. Results

### 3.1. Data capture

The ACSM and the AIM met both the data coverage and time requirements of the Directive (Table 2 and Table 3 respectively). It is noted that in 2018 the AIM was only operated for the period between 01 January, 2018 and 18 October, 2018 at Marylebone Road. The MARGA (Table 4) also met the data coverage and time requirements of the Directive at Auchencorth Moss, whereas at the Chilbolton Observatory it was found that in 2017, K^+^ did not meet the data capture objective and in 2018 K^+^, Ca^2+^ and Mg^2+^ did not meet the data capture objective. It is noted, that the MARGAs at both sites were replaced at the start of 2018 and low data capture is due to initial operational issues following the replacement of the instrument.

### 3.2. Equivalence

Expanded uncertainty analysis was performed on each location individually, as large datasets, such as the MARGA Melpitz (Case study 4), were found to greatly influence the results when datasets from different case studies were combined. In addition, in the absence of standardised operating procedures, set-up varied (cut-off and sampling inlet length) between sites and therefore direct comparison cannot be made between case studies. All the calculated expanded uncertainties with the orthogonal regressions for each species can be found in the supplementary material (Figs. S1–S45).

#### 3.2.1. ACSM and HR-TOF-AMS

Table 5 summarises the equivalence for the case studies #1 to #3 (Table 1) for the ACSM and HR-TOF-AMS. It was found that equivalence was possible for ${\text{NH}}_{4}^{+}$, ${\text{NO}}_{3}^{-}$ and ${\text{SO}}_{4}^{2-}$, though either the slope or the slope and intercept required correction to meet the equivalence criteria. In the only study that reported chloride (Revin, Case Study 1, Fig. S29), the ACSM failed to pass the expanded uncertainty criterion as expected. It is however noted that the reported concentrations were small (no Cl^−^> 2 μg m^−3^, Table 5) making the assessment on uncertainty challenging (RM mean = 0.122 μg m^−3^). In addition, the CM reported PM_1_ compared to PM_2.5_ reported by the RM. It is likely that the RM contained sea salt and the ACSM is known to be unable to report Cl^−^ from sea salt as it is a refractory compound. For that reason, ACSM chloride data have not been processed any further in this study.

#### 3.2.2. MARGA

Table 6 summarises the performance of the MARGA instruments in Case Studies 4 to 7. It is immediately clear that no set-up passed the W_cm_ criteria for all species but a combination of case studies provides evidence that the MARGA can pass the equivalence criteria for each species.

At Melpitz (Case Study 4) (Stieger et al., 2018), Na^+^, K^+^ and Ca^2+^ failed the W_cm_. Also, both Cl^−^ and ${\text{NO}}_{3}^{-}$ still had a significant slope after correction and therefore would not pass the equivalence test. Whereas at Kumpula (Case Study 5) (Makkonen et al., 2012) only K^+^ failed to meet the W_cm_ criteria of 50% even after correction, however there were only six data points and reported concentrations were low. In the same study, Ca^2+^ and Mg^2+^ also failed to meet the equivalence criteria, due to the intercept still being significant for both, as well as the slope for Mg^2+^, following correction.

For the Research Triangle Park site (Case Study 6), only ${\text{NH}}_{4}^{+}$, ${\text{NO}}_{3}^{-}$ and ${\text{SO}}_{4}^{2-}$ data were available but the site operated 2 MARGAs (Case Studies 6a and 6 b) in parallel against the RM of the US EPA (Rumsey et al., 2014). A disadvantage of this study used a cut-off of PM2.5 and the CM reported ~ PM26. As a result, ${\text{NO}}_{3}^{-}$, which is typically found in both the coarse and fine fractions, had the greatest uncertainty causing one instrument to fail the criterion (Table 6, Case Study 6a) with slopes ranging from 2.041 to 2.890 before correction. However, once the datasets were averaged and corrected the instrument passed the W_cm_ criterion (Case Study 6c, Fig. S14c). Ammonium also passed the criteria either as individual instruments or when averaged. For ${\text{SO}}_{4}^{2-}$ either as individual units or averaged both the intercept and slope correction was required to pass the W_cm_.

In the final MARGA case study at San Pietro Capofiume (Case Study 7), ${\text{NH}}_{4}^{+}$, ${\text{NO}}_{3}^{-}$ and ${\text{SO}}_{4}^{2-}$ passed the W_cm_ criterion even though the CM reported consistently higher concentrations. Potassium also passed equivalence without correction, though there is no significant correlation between the reported RM and CM (R^2^ = 0.161). The poor relationship is likely to be due to the low concentrations of ~0.05 μg m^−3^, which are below the detection limits of the MARGA when the IC uses injection loops rather than pre-concentrator columns that lower the detection limit (Table S1).

#### 3.2.3. AIM

The AIM at North Kensington (Case Study 8) passed the expanded uncertainty criteria for all species, with the exception of Na^+^, K^+^ and Ca^2+^ (Table 7). It was evident in studying the times series, ion balance and the theoretical concentration of sea salt (Figs. S46–47) that Na^+^ was overestimated by the CM compared to the RM. This would explain why it failed to pass the expanded uncertainty. K^+^ was also overestimated compared to the RM and could not be corrected.

## 4. Discussion

As previously discussed, (section 3.1) all three CMs met the data capture objectives, however, the expanded uncertainty criteria was not met for all species by all three CMs. Further discussion of this, and limitations associated with the candidate methods are provided in this section.

### 4.1. Performance of the expanded uncertainty analysis

**Fig. 1.**
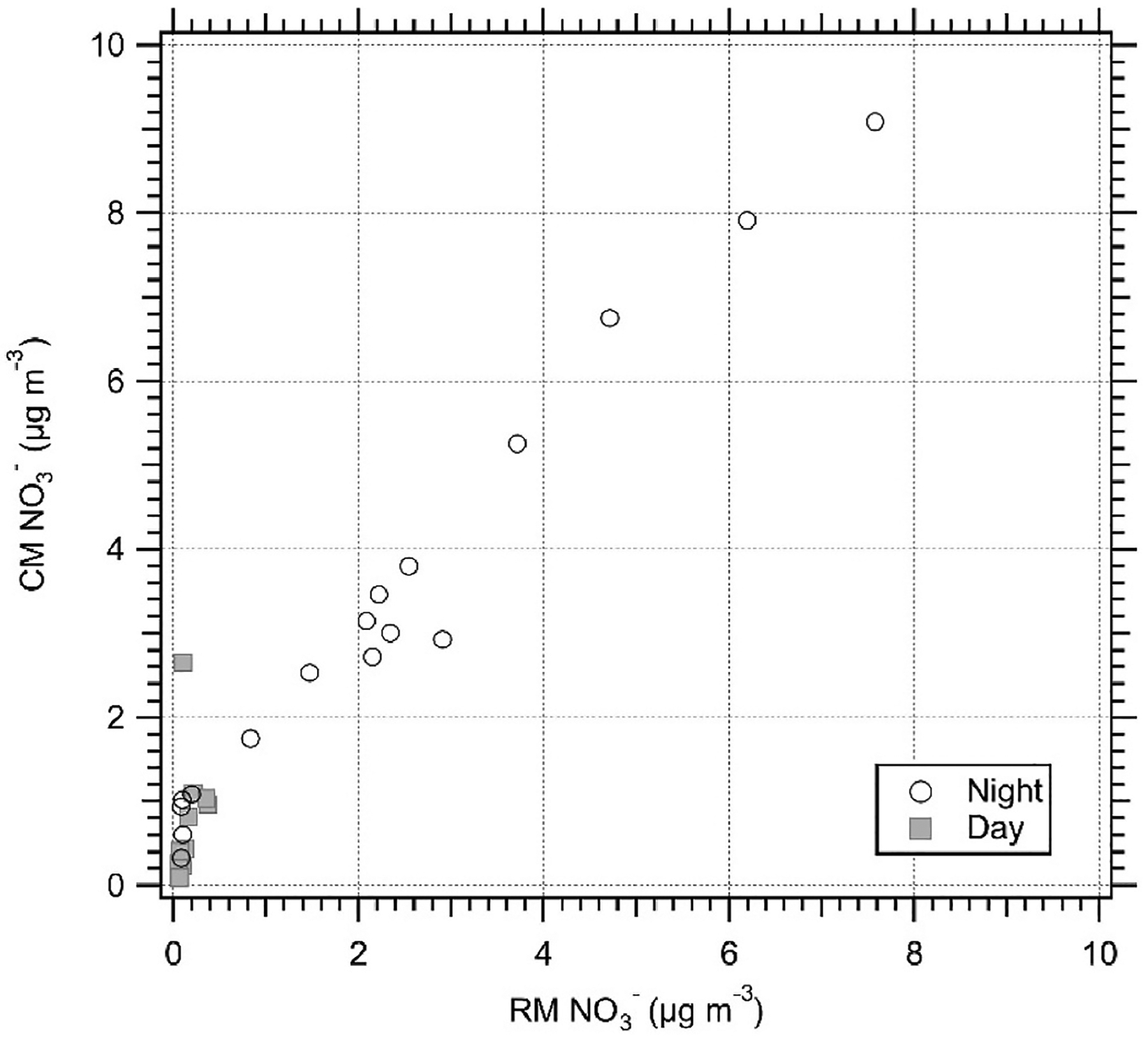
Twelve-hour PM_10_ ${\text{NO}}_{3}^{-}$ measurements at the San Pietro Capofiume field site (Case Study 7) with a MARGA as the CM, split into day and night.

For all case studies, the W_cm_ was passed for ${\text{NH}}_{4}^{+}$ following corrections. However, the corrections required (slope and/or intercept) for the CMs were not consistent between case studies, which is true for all species studied. This is likely due to the varying set-ups and calibration strategies, as well as varying meteorological conditions and chemical regimes between case studies, as outlined in Table 1. The expanded uncertainty criterion for ${\text{NO}}_{3}^{-}$ was passed by all except for the Melpitz data (Case Study 4), which still had a significant slope. Stieger et al. (2018) discussed the differences between filter and the MARGA for ${\text{NO}}_{3}^{-}$ and concluded that in summer NH_4_NO_3_ is lost from filters through volatilisation, leading to an underestimation, whereas in winter the filter reports higher concentrations compared to the MARGA. To investigate this hypothesis of volatilisation from filters the San Pietro Capofiume data was studied (Case Study 7) as sampling was for 12 h rather than 24 h (Table 1, Fig. 1). It was found that during the day ${\text{NO}}_{3}^{-}$ had a large uncertainty (W_cm_ = 11362 %), as concentrations were low, whereas at night, when a larger concentration range was reported (Table 8), the uncertainty met the criteria (W_cm_ = 2.26). No relationship however could be found to link the reported concentration difference between the RM and CM to mean temperature, as it is a controlling mechanism of volatilisation losses from the filter. The effect however could be masked by the low daytime concentrations that were challenging the detection limits of the MARGA that was operating with injection loops (Table S1). It is however beyond the scope of this phase 1 study to investigate the influence of meteorology.

Sulfate was the third species reported by all CMs in the case studies. All studies met the W_cm_ criteria. For the ACSM case studies only the slope was required to be corrected, whereas the HR-TOF-AMS (Case Study 3) required correction of both the slope and the intercept. As the HR-TOF-AMS was based at the coastal site of Mace Head it is expected that total ${\text{SO}}_{4}^{2-}$ reported by the RM includes a significant fraction of sea salt ${\text{SO}}_{4}^{2-}$ which cannot be detected by the HR-ToF-AMS due to its super-micron size and refractory nature.

Out of the case studies using either the ACSM or the HR-TOF-AMS, only one case study provided chloride data as the other studies had not calibrated their instruments for chloride. It was found in the study the ACSM failed the W_cm_ criteria (Revin, Case Study 1), which is not unexpected since the ACSM is insensitive to NaCl as the majority cannot be flash vapourised at 600 °C (Huang et al., 2018; Ovadnevaite et al., 2014) and the Cl^−^ reported is thought mainly to be in the form of NH_4_Cl. However there have been attempts to quantify seasalt Cl^−^ from HR-TOF-AMS high resolution data by quantifying the degree of the incomplete vaporisation or the instrument background signal (Ovadnevaite et al., 2012a; Schmale et al., 2013) therefore in a future equivalence study it is recommended that this possibility should be explored. The MARGA at the San Pietro Capofiume (SPC) site (Case Study 7) also failed on Cl^−^, whereas the other two MARGA case studies passed (Case Studies 4 and 5). This is probably due to the difference in the ambient average concentration, where it was 0.02 μg m^−3^ at SPC compared to 0.26 μg m^−3^ and 0.14 μg m^−3^ at Melpitz (Case study 4) and Kumpula (Case study 5), respectively. The AIM (Case Study 8) also passed the W_cm_ criteria for Cl^−^.

Only the two IC-based CMs (MARGA and AIM) are able to report base cations. Three of the datasets submitted for the MARGA reported Na^+^, all of which were below 2 μg m^−3^ in concentration. The dataset from Kumpula (Case Study 5) passed the expanded uncertainty criteria with an average reported concentration of 0.23 μg m^−3^, however the relationship was not strong, with an R^2^ = 0.61. The other two MARGA datasets did not pass (Case Studies 4 and 6). The AIM also did not pass the expanded uncertainty criteria (Case Study 7), where the average reported concentration was 1.04 μg m^−3^. Beccaceci et al. (2015) discuss that the AIM may have suffered from contamination, which would explain the overestimated Na^+^ concentrations.

For the remaining cations, K^+^, Ca^2+^ and Mg^2+^, not all studies passed the expanded uncertainty criteria and performance was variable for the ion chromatography CMs. Only the MARGA at the SPC site (Case Study 7) passed the criteria for equivalence for K^+^ out of the three MARGA case studies, which is surprising as SPC reported the lowest average concentration of 0.05 μg m^−3^, while Melpitz (Case study 4) and the Kumpula (Case study 5) sites reported 0.12 μg m^−3^ and 0.08 μg m^−3^, respectively. The AIM also failed to demonstrate equivalence; however, the average concentration of 0.03 μg m^−3^ was close to the instrument detection limit. For Ca^2+^, again it was only the SPC site in case study 7 that passed the expanded uncertainty criteria however no relationship could be found when studying the correlation. The SPC site however failed to pass the equivalence criteria for Mg^2+^. Instead, it was the Melpitz (Case study 4) and Kumpula (Case study 5), as well as the AIM (Case study 8) that passed the equivalence criteria for Mg^2+^.

### 4.2 Inlet set up

Under the EN12341:2014 standard (CEN/TC 264, 2014) sampling has to be carried out by using an inert, non-corroding, electrically conducting material such as stainless steel, anodized aluminium or aluminium alloy and it should not have any bends to minimise loses of aerosols. All the CMs presented were not automatically provided with an inlet by the manufacturer and so the inlet set up varied between sites (Table 1). Only the URG AIM used an anodized aluminium inlet with a vertical sampling position, so there was no bend as prescribed by the standard. The other CMs (MARGA, ACSM, HR-TOF-AMS) however all have a horizontal sampling position and therefore an inlet bend is included in the set-ups presented, which is likely to lead to aerosol losses. The inlet of the MARGA is a compromise design also to measure trace gases NH_3_ and HNO_3_ that are considered ‘sticky’ and choice of inlet material is therefore challenging. Evidence from previous studies (Neuman et al., 1999; Whitehead et al., 2008; Zhu et al., 2012) suggests that use of stainless steel or anodized aluminium, whilst minimising particle losses, would lead to adsorption losses of gases to the inlet walls. Therefore, MARGA inlets tend to be constructed of polytetrafluoroethylene (PTFE), perfluoroalkoxy (PFA) or polyethylene (PE), (see Table 1), with Teflon-coated size-selectors to minimise the losses of reactive gases. In the case studies presented there were no consistent lengths either but the EN12341:2014 standard stipulates that inlet length can be no more than 3 m. If the proposed CMs are to be considered in the future for the standard, then additional work would be required to establish a standard inlet design for the candidate methods.

### 4.3. Limitations of the candidate methods

The size cut-off of the ACSM (and HR-TOF-AMS) is controlled by the characteristics of the aerodynamic lenses that focus the particles during transfer into the vacuum. This is controlled by the vacuum aerodynamic diameter rather than the cut-off aerodynamic diameter, which is different in their dependencies on particle density. In the datasets presented, the ACSM instruments were equipped with the standard (PM_1_) aerodynamic lens, but more recently a PM_2.5_ lens was made available by the manufacturer. Most of the studies comparing ACSM to filters in literature are made using PM_1_ lenses and highlight the difficulties of comparing different size cut off instruments. The first PM_2.5_ ACSM setups had some issues with consistency in detecting larger particles, but lately advances in the inlet design, the use of a lens with improved transmission efficiency and the use of a capture vaporizer in the instrument have largely solved the issues in the new generation instruments (Xu et al., 2017; Zhang et al., 2017). It would therefore have to be investigated if the equivalence demonstrated was possible for the ACSM with a PM_2.5_ lens too.

The estimate of total mass loading from the ACSM requires the knowledge of the collection efficiency (CE) for the instrument. The CE of the ACSM needs to be evaluated regularly for the instrument and can depend on the chemical composition and on the relative humidity of the sampled air (Middlebrook et al., 2012). To reduce uncertainties on CE the air is sampled through a Nafion drier placed in front of the ACS-M/AMS inlet. This will decrease the relative humidity, which is measured by a RH sensor between the drier and the ACSM/AMS. The RH is maintained below 40% to avoid any influence on the CE evaluation. A typical technique used to validate the CE involves a comparison between a volume concentration obtained from the ACSM data using the compounds densities and a volume concentration derived from a co-located Scanning Mobility Particle Sizer (SMPS) spectrometer or from a nephelometer. However, the recent development of the aforementioned capture vaporizer with near unity CE is likely to reduce this uncertainty in the future. This is applicable to the ACSM, but is incompatible with the sizing of the HR-TOF-AMS.

The main issue in using the ACSM is that not all species covered by the Directive can be measured by this method, as only non-refractory compounds can be detected by the ACSM. Species like sodium chloride, sodium sulfate, sodium nitrate and the dust/crustal components such as K^+^, Ca^2+^ and Mg ^+^ are not included in the aerosol mass loading provided by the instrument and so to meet the objective would require the presence of additional monitoring equipment. It is however reported in the literature that the HR-TOF-AMS has been used to derive NaCl from sea salt (Ovadnevaite et al., 2012b). This said, the ACSM or the HR-TOF-AMS additionally provides a quantitative measure of organic aerosol mass with additional information that can be used for its source apportionment.

In the absence of internationally agreed standard operating procedures (SOP) for MARGAs and related instruments, implementations vary significantly, and this makes comparisons difficult to interpret and generalise. For example, in the case studies for W_cm_ presented, the instrument set-ups are different compared to the MARGAs already used in routine monitoring in the UK (Twigg et al., 2015) as the MARGA operated at Auchencorth Moss, a remote rural background site (Malley et al., 2014), which was used for studying the data capture (but not for the W_cm_ assessment due to the lack of a RM measurement), operates with pre-concentration columns rather than injection loops to achieve lower detection limit (see Table S1). Similarly, whilst all the MARGAs in the studies presented used a cation eluent based on nitric acid (HNO_3_), the UK MARGA network the Auchencorth Moss uses methanesulfonic acid (MSA) and the Chilbolten instrument p-toluenesulfonic acid instead, because a carry-over of HNO_3_ and artefact in the anion analysis for nitrate has been observed in some of the systems and had to be corrected for in the San Pietro Capofiume data (Makkonen et al., 2012). Therefore, investigations would be required to determine the impact of pre-concentration columns and cation eluent on achieving equivalence, and if a common optimum SOP is required.

For the post processing of chromatograms, the MARGA instrument operators in these studies would have likely been provided with the reanalysis tool by Metrohm. The use of this tool can be challenging due to inconsistent integration of chromatograms as demonstrated by Chen et al. (2017), who recommended the use of another reintegration software (Chromeleon V7.3, Thermo Scientific, Dionex). The issue of inconsistent integration, however, is thought to be resolved in the new model of MARGA (MARGA, 2060), as it uses a new software (MagicIC Net, Metrohm) for the integration of chromatograms.

The case studies presented to demonstrate equivalence all use an earlier model of the MARGA that is no longer commercially available. There are to date no datasets available to demonstrate equivalence using the new MARGA 2060 model. In the 2060 model both the air flow rate and liquid flowrates can be reduced, as well as the WRD being shortened, to try and minimise the liquid consumption. In addition, the mass flow controllers used in the earlier MARGA model, have been replaced by a critical orifice. The use of the critical orifice raises concerns since the mass flow rate is determined by temperature and pressure and controls the speed of the particles going through a cut-off. The inability of the flow rate to respond to changes in ambient temperature and pressure to keep the volumetric flowrate at the size cut constant will likely result in changes in the reported cut-off. Under the current configuration, the MARGA 2060 using a critical orifice would fail to meet the EN12341:2014 standard for the size cut-off of PM_2.5_. The case studies, however, have demonstrated that even with a different size cut-off, equivalence is still possible in many conditions (Case Study 6), at least for components that are dominated by the accumulation mode, contained within PM_1_, PM_2.5_ and PM_10_. Further investigations would be required to determine if the 2060 model could demonstrate equivalence.

The AIM is normally operated with a PM_2.5_ cyclone. During the field test period in 2013 a size selective PM_10_ monitoring head was in operation at North Kensington. Although the method show an overall good correlation for ${\text{NO}}_{3}^{-}$, ${\text{SO}}_{4}^{2-}$, Cl^−^, ${\text{NH}}_{4}^{+}$ and Mg^2+^ there is poor correlation found for Na^+^, K^+^ and Ca^2+^. Beccaceci et al. (2015) outline possible explanations for the differences including positive instrument bias due to contamination, efficiency of particle extraction and removal of gases, but this will require further investigation.

### 4.4. Limitations of the EN16913:2017 standard

The objective of PM_2.5_ chemical composition data under the EU Air Quality Directive (AQD) 2008/50/EC is to provide information on the levels in the background, which is used to assess the potential contribution from long-range transport, to support source apportionment analysis of the contributors to total PM_2.5_, and for understanding the behaviour of specific PM pollutants (EC, 2015). Under the EN16913:2017 a 24-h average is produced for each species, compared to the proposed CMs which produce online results at a higher time resolution of 1 h or better. The current EN16913:2017 standard of 24 h makes interpretation with regards to long-range transport and source apportionment challenging as atmospheric conditions change at a higher temporal resolution. This is especially important for disentangling air quality events in near real time to determine which aspects are from domestic (national) emissions and which are the result of long-range transport (imported). The current EN16913:2017 standard makes it impossible to respond to air quality events in near real time as it has a delay in reporting due to samples only being collected typically on a weekly frequency (though at some sites this delay can be up to 16 days), followed by analysis offline in a laboratory. The advantage of the sub-daily resolution from potential CMs is that it provides additional information on the temporal pattern of emissions and the thermodynamic effects on gas/aerosol partitioning.

The EN16913:2017 methodology may not accurately report atmospheric concentrations and acknowledges that up to 30% losses of volatile compounds such as NH_4_NO_3_ can occur (CEN/TC 264, 2017). The losses experienced by the RM for PM_2.5_ mass sampling makes this imperfect measurement data less useable for the assessment of atmospheric chemistry and transport models or to constrain emissions. Indeed, some equivalence datasets, such as the summer MARGA data from Melpitz, appear to have been affected by this shortcoming of the RMs. Rather than attempting to match an imperfect method (RM), future work should also investigate whether CMs can be artificially degraded through a simulation of the impact of the losses that would be encountered by the RM, likely as a function of temperature and humidity.

Evidence suggests that the organic fraction of PM_2.5_, not currently reported under the Directive may be of greatest concern to human health for acute exposure to PM due to its oxidative potential (Daellenbach et al., 2020), compared to the inorganic species covered by the EN16913:2017 standard. As organic PM is complex, high temporal resolution measurements would facilitate identification of the sources necessary to develop and monitor mitigation strategies.

### 4.5. Other potential candidate methods

There are other methods available, which could potentially report components of the EN16913:2017 standard. The UK now operates insitu X-ray fluorescence spectrometry (XRF) instruments (Xact 625 Ambient Metals Monitor, Cooper Environmental Services) at its three UK NERC Urban Supersites, which is a non-destructive method to provide elemental composition. The system is able to quantify 24 elements (Si, S, Cl, K, Ca, Ti, V, Cr, Mn, Fe, Co, Ni, Cu, Zn, As, Se, Cd, Sn Sb, Ba, Pt, Hg, Pb, Bi, Pd) including K and Ca, which are in the EN16913:2017 standard. Furger et al. (2017) carried out a comparison of daily PM_10_ filters against the XRF method. It was found that for K and Ca there was excellent correlation to the daily average filters. Tremper et al. (2018) also investigated the performance of the XRF both in the laboratory and in the field. The study concluded that Ca and K compared well to filters in the field but there was a positive difference in the slopes when compared to AMS or AIM (for Ca^2+^, Cl, K^+^ and ${\text{SO}}_{4}^{2-}$), which was attributed to the differences in size, volatility, and water solubility of the PM measured. It is therefore recommended that any future work to demonstrate equivalence to EN16913:2017 should include XRF method, also to assess whether the combination of ACSM and XRF could provide equivalence for all compounds of interest.

### 4.6. Requirements for a future equivalence study

This study is the first systematic comparison between the EN16913:2017 reference method (or similar filter methods) and potential CMs using existing and available datasets to determine if equivalence is possible. It is noted that the studies assessed were in most cases not specifically set up to compare the methods with datasets being serendipitous. As a result, this is not a specifically designed equivalence study, rather a first step which demonstrates the clear need for one. None of the CMs presented here operated with a PM_2.5_ cut-off (the AMS for this size fraction being a recent innovation) making evaluating equivalence challenging, as size distribution varies between ions. Not all of the CMs have an internationally recognised standard operating procedure (SOP) except for the ACSM, for which one was developed under the European Aerosol, Cloud, and Trace Gases Research Infrastructures (ACTRIS, https://www.actris-ecac.eu/pmc-non-refractory-organics-and-inorganics.html). A future equivalence study should be designed to follow the Guide to Demonstrate Equivalence (GDE) (GDE, 2010).

A future equivalence study would have to ensure:

1. All set-ups are prescribed in order that datasets can be comparable, including operating with a PM_2.5_ cut-off.
2. All CMs have a user-community agreed SOP including quality control and quality assurance methodology.
3. Both laboratory and field studies will be required to be undertaken to assess the uncertainty compared to the RM.
4. Uncertainties will need to be quantified for sampling efficiency, analyte selectivity, blanks, calibration, repeatability and instrument drift both under laboratory conditions and in the field.
5. Comparability between RM and CM, as well as the ‘between sample’ uncertainty of the CM will need to be assessed under field conditions.
6. GDE recommends 4 minimum comparison field studies should be undertaken covering different chemical and meteorological regimes.

## 5. Conclusions

This desk study has provided initial evidence that the MARGA has the potential to demonstrate equivalence for all species included in the EN16913:2017 standard, whereas the ACSM/HR-TOF-AMS has the potential to demonstrate equivalence for ${\text{NH}}_{4}^{+}$, ${\text{NO}}_{3}^{-}$ and ${\text{SO}}_{4}^{2-}$. The AIM has demonstrated equivalence for ${\text{NH}}_{4}^{+}$, ${\text{NO}}_{3}^{-}$, ${\text{SO}}_{4}^{2-}$, Cl^−^, and Mg^2+^, however further investigations would be required to understand if under optimised conditions, the AIM was possible for Na^+^, K^+^ and Ca^2+^ to pass the equivalence criteria.

There are operational differences between MARGA instruments including cation eluents, pre-concentration columns, and inlet set-up; thus further investigations would be required to determine if this alters the potential for equivalence. This study also did not include XRF spectrometry instruments. It is recommended to include XRF in any future equivalence study, which could be a good complement to the ACSM, which returned promising results for ${\text{SO}}_{4}^{2-}$, ${\text{NH}}_{4}^{+}$ and ${\text{NO}}_{3}^{-}$, but cannot measure base cations or the full suite of chloride compounds.

None of the case studies presented operated with a PM_2.5_ cut-off for the candidate method and therefore further investigations are required to confirm the above conclusions. It is therefore recommended that the next stage to undertake consists of targeted laboratory and field studies of the CMs with the PM_2.5_ cut-offs compared to the EN16913:2017 standard to demonstrate equivalence.

## Supplementary Material

Supplement1

## Figures and Tables

**Table 1:** Description of method set up of paired data sets used to investigate the potential equivalence for the candidate methods for EN16913:2017 standard.

Case study	1	2	3	4	5	6	7	8
Geographical area	Revin, France	Barcelona, Spain	Mace Head, Ireland (O′Dowd et al., 2014)	Melpitz, Germany	Kumpula, Helsinki, Finland	Research Triangle Park, North Carolina, USA	San Pietro Capofiume, Italy	North Kensington, London, UK
Longitude	49° 54′ 60″ N,	41° 23′ 14.3″ N	53° 19′ 34″ N,	51°32′N	60°12′11.1″N	35.89 N	44° 39′ N	51.521050 N
Latitude	04° 38′ 29″ E	02° 06′ 56.6″ E	9° 54′ 14″ W	12°56′E	24°57′40.7″E	78.87 W	11° 37′ E	−0.213492
Classification	Rural background	Urban background	Rural background	Rural Background	Urban Background	Urban Background	Rural Background	Urban background
Metres above mean sea level (m)	395	80	5	86	26	92	11	5
CM inlet length (m)	2.5	2–3	10 m	3.6	1.7	4	Not available	1–2
Inlet material for CM	Stainless steel		Stainless steel sampling duct	Teflon-coated PM10 cyclone and 3.5 m long polyethylene tube	Teflon coated cut-off inlet with polyethylene tubing	Acrylic inertial separator with polyethylene tubing	Teflon coated cyclone with polyethylene tubing	PM_10_ head attached to an anodized aluminium tube
Network affiliations	EMEP, ACTRIS, GAW	ACTRIS	EMEP, ACTRIS, GAW	EMEP GAW ACTRIS	Intensive field study	Intensive field study	Intensive field study	UK PNC
Average temperature	4.3 °C	18.5 °C	–	10.0 °C	6.7 °C	16.2 °C	23.6 °C	–
Sampling periods (Maximum number of data points used^#^)	November 30, 2017–March 30, 2018 (*N* = *18*)	May 05, 2014–24/05/2015 and September 02, 2017–October 27, 2018 (*N* = *152*)	January 01, 2009–December 30, 2012 (*N* = *385*)	January 01, 2010–December 31, 2014 (*N* = *1488*)	February 05, 2010–May 05, 2010 (*N = 86*)	September 08, 2010–October 08, 2010 (*N* = *60*)	June 14, 2012–July 09, 2012 (*N* = *29*)	January 03, 2013–December 27, 2013 (*N* = *33*)
Reference Method (RM)	**PM**_**2.5**_ 150 mm diameter quartz filters, prefired at 500 degC during 24 h. Digitel DA80 equipped with a Digitel PM_2.5_ head at a flow rate of 30 m^3^ h^−1^	**PM**_**1**_ 150 mm-diameter quartz fibre filters using Digitel automatic high volume (30 m^3^ h^−1^) samplers.	**PM**_**2.5**_ PTFE filters using Partisol sampler (1 m^3^ h^−1^).	**PM**_**10**_ quartz filters sampled with a Digitel DHA-80 at 30 m^3^ h^−1^ during 24 h. Filters preheated at 105 °C.	**PM**_**10**_ Teflon filters sampled at 1 m^3^ h^−1^	**PM**_**2.5**_ Denuder – Teflon/Nylon filter pack sampled at 0.6 m^3^ h^−1^	**PM**_**1**_ filters quartz filters sampled with a Digitel DHA-80 at 30 m^3^ h^−1^ during 24 h. Filters preheated at 105 °C.	**PM**_**10**_ Quartz
Filter change time	09:00	00:00	08:00	00:00	00:00	07:00 19:00	09:00 21:00	00:00
Candidate method (CM)	**PM**_**1**_ ACSM	**PM**_**1**_ ACSM	**PM**_**1**_ HR-TOF-AMS	**PM**_**10**_ MARGA	**PM**_**10**_ MARGA	**PM**_~26_* MARGA	**PM**_**1**_ MARGA	**PM**_**10**_ URG AIM
Reference	Bourin et al., 2019, Bourin, 2020	Via et al., 2021	Ovadnevaite et al., 2012b, 2014	Stieger et al., 2018	Makkonen et al., 2012	Rumsey et al., 2014	Sandrini et al., 2016	Beccaceci et al., 2015

Note: The EN16913:2017 only permits quartz filters for sampling.

* It has been estimated that due to the inlet set-up that the PM cut-off was approximately 26 μm in aerodynamic diameter.

# For specific *N* for each ion, refer to Figs. S1–S45.

**Table 2:** Data capture (hourly resolution) and number of days with >90% data capture in 24 h achieved at North Kensington, London for the PM_1_ ACSM for the years 2016–2017. The directive target is 52 days per year. n/a: refractory species are not quantified by this method.

Species	Data capture (%)		# days	
	2016	2017	2016	2017
${\text{NH}}_{4}^{+}$	55	75	168	243
Na^+^	n/a	n/a	n/a	n/a
K^+^	n/a	n/a	n/a	n/a
Ca^2+^	n/a	n/a	n/a	n/a
Mg^2+^	n/a	n/a	n/a	n/a
Cl^−^	n/a	n/a	n/a	n/a
${\text{NO}}_{3}^{-}$	55	75	168	243
${\text{SO}}_{4}^{2-}$	55	75	168	243

**Table 3:** Data capture (hourly resolution) and number of days with >90% data capture in 24 h at North Kensington (NK) and Marylebone Road (MR), London sites for the PM_10_ AIM for the years 2016–2018 (data downloaded from UK-Air on the November 09, 2020). The directive target is 52 days per year.

Species	Data capture (%)						# days					
	2016		2017		2018*		2016		2017		2018*	
	NK	MR	NK	MR	NK	MR	NK	MR	NK	MR	NK	MR
${\text{NH}}_{4}^{+}$	70	63	77	44	53	67	224	188	239	122	133	207
Na^+^	69	80	78	46	52	72	221	242	246	128	126	223
K^+^	69	77	75	46	58	71	220	234	233	129	141	222
Ca^2+^	62	70	79	45	58	72	198	210	248	126	143	224
Mg^2+^	70	80	75	46	58	72	220	240	234	128	144	220
Cl^−^	68	79	75	48	57	48	218	256	237	142	139	148
${\text{NO}}_{3}^{-}$	69	80	76	48	61	48	218	258	239	142	150	149
${\text{SO}}_{4}^{2-}$	68	77	71	46	57	48	219	247	225	139	240	149

* Maximum number of days possible is 291 as instrument was only operational from 01 January, 2018 to 18 October, 2018 at North Kensington, London.

**Table 4:** Data capture (hourly resolution) and number of days with >90% data capture in 24 h at Auchencorth Moss (ACTH) and Chilbolton Observatory (CHBO) sites for the PM_2.5_ MARGA for the years 2016–2018 (data downloaded from UK-Air on the November 09, 2020). The directive target is 52 days per year. In bold are the times where the minimum number of days is not achieved in a year.

Species	Data capture (%)						# days					
	2016		2017		2018*		2016		2017		2018*	
	ACTH	CHBO	ACTH	CHBO	ACTH	CHBO	ACTH	CHBO	ACTH	CHBO	ACTH	CHBO
${\text{NH}}_{4}^{+}$	63	54	52	81	73	65	193	176	128	270	202	169
Na^+^	62	53	52	63	73	73	189	168	128	146	204	195
K^+^	63	54	53	24	73	10	193	175	131	**11**	209	**27**
Ca^2+^	61	54	55	81	74	8	184	176	137	271	211	**20**
Mg^2+^	63	54	55	81	74	10	193	176	137	272	211	**27**
Cl^−^	65	57	74	77	80	72	206	189	216	257	232	189
${\text{NO}}_{3}^{-}$	65	57	74	78	80	73	206	189	216	264	232	195
${\text{SO}}_{4}^{2-}$	65	57	74	77	80	73	206	188	216	258	232	197

* MARGA instrument replaced.

**Table 5:** Summary of equivalence for the ACSM and HR-TOF-AMS case studies. Highlighted in grey are where the expanded uncertainty (W_cm_), slope or intercept fail the equivalence criteria. ${\text{nssSO}}_{4}^{2-}$: non sea salt ${\text{SO}}_{4}^{2-}$. Corrected - 2nd orthogonal regression was carried out to calculate the expanded uncertainty, after data had been calibrated (corrected) for either the slope (S), the intercept (I) or both (SI), based on the criteria outlined in Section 2.3.

		Raw						Corrected	
Case study	Species	Slope	Intercept	W_cm_ (%)	R^2^	n	% >2 μg m^−3^	S,I or SI corrected	W_cm_ (%)
1	${\text{SO}}_{4}^{2-}$	0.544	0.04	90	0.605	18	11	S	37
	${\text{NO}}_{3}^{-}$	0.927	0.493	7	0.96	18	27.8	SI	16
	${\text{NH}}_{4}^{+}$	0.742	0.201	47	0.949	18	11	SI	4
	Cl^−^	−0.078	0.024	215.2	0.478	18	0	SI	132.5
2	${\text{SO}}_{4}^{2-}$	1.092	−0.069	19	0.84	147	25.9	S	0.5
	${\text{NO}}_{3}^{-}$	1.829	0.051	166	0.79	152	9.2	S	11.8
	${\text{NH}}_{4}^{+}$	1.7	−0.119	138	0.75	152	2.6	SI	13.6
3	${\text{nssSO}}_{4}^{2-}$	1.144	0.097	30.8	0.85	385	5.2	SI	2.3
	$\text{Total}\;{\text{SO}}_{4}^{2-}$	1.179	−0.045	34.9	0.84	384	5.5	SI	3.1
	${\text{NO}}_{3}^{-}$	0.754	−0.111	51.5	0.83	334	6	SI	5.2
	${\text{NH}}_{4}^{+}$	0.851	−0.002	30.0	0.85	348	2.9	S	2.7

**Table 6:** Summary of equivalence for the MARGA (case studies 4 to 7). Case study 6a (CM1) and 6 b (CM2), are collocated MARGAs are the same station, whereas 6c is the combined MARGA datasets from the same station (CM1 and CM2). Highlighted in grey are where the expanded uncertainty, slope or intercept fail the equivalence criteria. Corrected - 2nd orthogonal regression was carried out to calculate the expanded uncertainty, after data had been calibrated (corrected) for either the slope (S), the intercept (I) or both (SI), based on the criteria outlined in Section 2.3. N/A – not applicable.

		Raw						Corrected		
Case study	Species	Slope	Intercept	W_cm_ (%)	R^2^	n	% > 2 μg m^−3^	S,I or SI corrected	W_cm_ (%)	S, I, or SI still significant following correction
4	Cl^−^	0.648	0.079	69	0.852	710	2.5	SI	6.7	S
	${\text{SO}}_{4}^{2-}$	0.826	0.016	35	0.907	1475	45	S	6.6	No
	${\text{NO}}_{3}^{-}$	0.679	0.564	55	0.875	1488	55	SI	25	S
	${\text{NH}}_{4}^{+}$	0.822	−0.100	38	0.865	1453	33	SI	2.4	No
	Mg^2+^	0.731	0.061	53	0.587	109	0	SI	19	No
	Na^+^	0.411	0.070	116	0.567	333	0	SI	53	SI
	K^+^	0.563	0.024	87	0.414	151	0	SI	67	SI
	Ca^2+^	2.829	−0.210	362	0.128	343	0	SI	146	SI
5	Cl^−^	0.772	0.045	45	0.831	39	0	SI	4.9	No
	${\text{SO}}_{4}^{2-}$	0.846	0.232	26	0.982	86	44.2	SI	0.2	No
	${\text{NO}}_{3}^{-}$	0.930	0.413	5.7	0.935	84	25.0	SI	0.4	No
	${\text{NH}}_{4}^{+}$	0.991	−0.374	9.2	0.822	74	9.5	I	1.7	No
	Mg^2+^	3.957	−0.043	591	0.716	86	0	SI	49	SI
	Na^+^	0.736	−0.089	55	0.608	35	0	SI	20	No
	K^+^	−0.054	0.122	208	0.020	6	0	SI	8306	SI
	Ca^2+^	3.505	0.027	502	0.846	81	0	S	29	I
6a	${\text{SO}}_{4}^{2-}$	0.973	0.281	0.2	0.996	60	56.7	SI	0.2	No
	${\text{NH}}_{4}^{+}$	1.031	0.028	6.8	0.972	60	3.3	N/A	N/A	No
	${\text{NO}}_{3}^{-}$	2.890	−0.400	370	0.797	60	0	SI	52	No
6 b	${\text{SO}}_{4}^{2-}$	0.978	0.208	0.2	0.995	60	56.7	SI	0.2	No
	${\text{NH}}_{4}^{+}$	0.986	0.079	1.2	0.960	60	3.3	I	2.75	No
	${\text{NO}}_{3}^{-}$	2.041	−0.244	203	0.810	60	0	SI	27	No
6c	${\text{SO}}_{4}^{2-}$	0.975	0.246	0.1	0.996	60	56.7	SI	0.1	No
	${\text{NH}}_{4}^{+}$	1.007	0.054	2.6	0.969	60	3.3	I	1.5	No
	${\text{NO}}_{3}^{-}$	2.447	−0.316	283	0.809	60	0	SI	37	No
7	Cl^−^	3.903	0.14	584	0.492	29	0	SI	324	S
	${\text{SO}}_{4}^{2-}$	0.946	0.539	0.1	0.856	29	55.2	I	11.0	No
	${\text{NO}}_{3}^{-}$	1.214	0.489	52.6	0.95	29	34.5	SI	5.8	No
	${\text{NH}}_{4}^{+}$	1.249	−0.059	48.7	0.922	26	19.2	S	10.3	No
	Mg^2+^	21.680	−0.164	4134	0.003	23	0	SI	343	SI
	Na^+^	3.371	−0.026	474	0.141	21	0	SI	310	SI
	K^+^	1.032	−0.023	6.0	0.161	12	0	N/A	N/A	No
	Ca^2+^	0.978	0.050	3.4	0.088	26	0	I	4.45	No

**Table 7:** Summary of equivalence for the AIM (Case Study 8). Highlighted in grey are where the expanded uncertainty, slope or intercept fail the equivalence criteria. Corrected - 2nd orthogonal regression was carried out to calculate the expanded uncertainty, after data had been calibrated (corrected) for either the slope (S), the intercept (I) or both (SI), based on the criteria outlined in Section 2.3.

	Raw						Corrected		
Ion	Slope	Intercept	W_cm_ (%)	R (EC, 2015)	n	% >2 μg m- (Tørseth et al., 2012)	S,I or SI corrected	W_cm_ (%)	S, I, or SI still significant following correction
Cl^−^	0.585	0.439	74	0.901	32	25	SI	5	No
${\text{SO}}_{4}^{2-}$	0.896	−0.226	26	0.931	33	27	S	4	No
${\text{NO}}_{3}^{-}$	0.895	0.26	28	0.895	33	42	S	30	No
${\text{NH}}_{4}^{+}$	1.493	0.439	105	0.890	33	18	SI	17	No
Mg^2+^	0.934	0.001	13	0.963	33	0	S	0.3	No
Na^+^	1.773	0.580	167	0.441	33	9.1	SI	58	S
K^+^	52.347	−1.202	10246	0.003	33	0	SI	230	SI
Ca^2+^	0.556	0.142	86	0.446	33	0	SI	61	No

**Table 8:** W_cm_ of PM_10_ ${\text{NO}}_{3}^{-}$ at San Pietro Capofiume reported by the CM (MARGA) for the whole period and separated into to day and night. Highlighted in grey are where the expanded uncertainty, slope or intercept fail the equivalence criteria. Corrected - 2nd orthogonal regression was carried out to calculated the expanded uncertainty, after data had been calibrated (corrected) for either the intercept (I) or both (SI), based on the criteria outlined in Section 2.3

	Raw						Corrected	
Ion	Slope	Intercept	W_cm_ (%)	R (EC, 2015)	n	% >2 μg m^−3^	S,I or SI corrected	W_cm_ (%)
all	1.214	0.489	52.64	0.95	29	34.5	SI	5.8
day	20.591	2.383	3881.19	0.154	12	0	SI	11362
night	1.011	0.123	16.39	0.882	10	30	I	2.26

## Data Availability

Data will be made available on request.

## HIGHLIGHTS

- Comparison of EN16913:2017 filter method for speciated inorganic PM_2.5_ against automatic methods.
- Automatic methods have the potential to demonstrate equivalence for some or all species.
- Further field and laboratory studies are required to demonstrate equivalence for speciated PM_2.5_
