# Supplementary material for: The potential of high temporal resolution automatic measurements of PM2.5 composition as an alternative to the filter-based manual method used in routine monitoring
Source: Atmos Environ (1994). Author manuscript; Available in PMC 2024 Dec 15. (PMC11534065; doi:10.1016/j.atmosenv.2023.120148)
Supplement: Supplement1 [file NIHMS2022350-supplement-Supplement1.docx]

**Supplementary Information:**

**The potential of high temporal resolution automatic measurements of PM_2.5_ composition as an alternative to the filter-based manual method used in routine monitoring**

**Marsailidh M. Twigg^1^, Chiara F. Di Marco^1^, Elizabeth A. McGhee^2^, Christine F. Braban^1^, Eiko Nemitz^1^, Richard J.C Brown^2^, Kevin C. Blakley^2^, Sarah R Leeson^1^, Agnieszka Sanocka^3^, David C. Green^4^, Max Priestman^4^, Veronique Riffault^5^, Aude Bourin^5^, Maria Cruz Minguillón^6^, Marta Via^6^, Jurgita Ovadnevaite^7^, Darius Ceburnis^7^, Colin O’Dowd^7^, Laurent Poulain^8^, Bastian Stieger^8^, Ulla Makkonen^9^, Ian C. Rumsey^10^_,_ Gregory Beachley^11^, John T. Walker^10^ and David M. Butterfield^2^.**

^1^ UK Centre for Ecology & Hydrology, Bush Estate, Penicuik, UK, EH26 0QB

^2^ National Physical Laboratory, Hampton Road, Teddington, London, UK, TW11 0LW

^3^ Ricardo Energy & Environment, Wantage, England, UK

^4^ MRC Centre for Environment and Health, Environmental Research Group, Imperial College London, UK

^5^ IMT Nord Europe, Institut Mines-Télécom, Univ. Lille, Centre for Energy and Environment, F-59000 Lille, France

^6^ Institute of Environmental Assessment and Water Research (IDAEA-CSIC), Barcelona, 08034, Spain

^7^ The Ryan Institute’s Centre for Climate and Air Pollution Studies, School of Natural Sciences, University of Galway, Galway, H91 CF50, Ireland

^8^ Atmospheric Chemistry Department (ACD), Leibniz Institute for Tropospheric Research (TROPOS), Permoserstr. 15, 04318 Leipzig, Germany

^9^ Finnish Meteorological Institute, 00560, Helsinki, Finland

^10^ Office of Research and Development, US Environmental Protection Agency, Research Triangle Park, NC 27711, USA

^11^ Office of Atmospheric Programs, US Environmental Protection Agency, Washington, DC 20460, USA

Corresponding author: Marsailidh Twigg ([sail@ceh.ac.uk](mailto:sail@ceh.ac.uk), UK Centre for Ecology & Hydrology, Bush Estate, Penicuik, EH26 0QB)

Four phases to demonstrate equivalence for ambient air quality

To demonstrate equivalence there are four phases (Table S1). This study has provided the first phase to determine if any of the automatic instruments proposed could be recommended as a potential candidate method (CMs) for the EN16913:2017 standard.

**Table S1** Summary of the four phases of the demonstrating equivalence. Text is taken from the Guidance Demonstration of Equivalence (January 2010)*^1^*. *ENV 13005 has now superseded by ISO/IEC Guide 98-3:2008 Guide to the Expression of Uncertainty in Measurement (GUM).

| **Phase** | **Description of activity** |
| --- | --- |
| 1 | Initial non experimental pre-assessment to check whether the candidate method has the potential for fulfilling the data quality objectives in the directives on data capture and measurement uncertainty. |
| 2 | Assessment of the uncertainty of the candidate method using an approach based on the principles of ENV 13005 (phase 8)^2^* in a series of laboratory tests. |
| 3 | The performance of a series of field tests for confirmation of the findings of the laboratory tests in which the candidate method is tested side-by-side to the reference method; the ‘lack of-comparability’ is tested on the basis of the performance of linear regression with symmetric treatment of both variables, i.e., with uncertainties attributed to both variables. |
| 4 | The evaluation of the resulting uncertainties by comparison of:   - laboratory uncertainty and the uncertainty data quality objective - field uncertainty and laboratory uncertainty - field uncertainty and the uncertainty data quality objective. |

Summary of the methodology used to calculate the expanded uncertainty

Equivalence is determined by comparing paired datasets of the candidate method (CM) to the reference method (RM) by studying the correlation using orthogonal regression plots, as it is assumed the relationship between the CM and RM is linear where:

$$y_{i}=a+bx_{i}$$

The expanded uncertainty calculated “…is a function of the sum of the relative residuals from the orthogonal regression, the concentration from the original regression equation at the Limit Value and the between sampler uncertainty of the … reference method.”^3^. The uncertainty of the sum of the relative residuals from the orthogonal regression is calculated by:

$$u_{CR}^{2}\left( y_{i} \right)= \frac{RSS}{\left( n-2 \right)}-u^{2}\left( x_{i} \right)+\left[ a+\left( b-1 \right)x_{i} \right]^{2}$$

where *RSS* is the sum of residuals resulting from the orthogonal regression and *u(x_i_)* is the uncertainty of the standard method. In this case a value of 0.67 µg m^-3^ was used for the uncertainty of the standard method. The combined uncertainty of the candidate method ($w_{CM}^{2}\left( y_{i} \right))$ is calculated using the concentration limit value ($y_{i}^{2})$. In this case the limit value was set to 10 µg m^-3^ where:

$$w_{CM}^{2}\left( y_{i} \right)=\frac{u_{CR}^{2}\left( y_{i} \right)}{y_{i}^{2}}$$

The expanded relative uncertainty is then calculated by multiplying $w_{CM}^{2}\left( y_{i} \right)$ by a coverage factor *k*, reflecting the number degrees of freedom resulting from the determination of $w_{CM}^{2}\left( y_{i} \right)$:

$$W_{cm}=k{\cdot w}_{CM}^{2}\left( y_{i} \right)$$

Full details of all the equations used by the equivalence template (<https://ec.europa.eu/environment/air/quality/assessment.htm>) to calculate the slope, intercept and expanded uncertainty are outlined in the Guide to demonstrating equivalence (2010)^1^.

Limits of detection of the candidate methods.

Table S2 to S4 below summarises the detection limits of the candidate methods used in this study. The MARGA, ACSM and HR-TOF-AMS present limits from the manufacturer, whereas the AIM limits were calculated during a field deployment.

**Table S2** Detection limits (hourly sampling) of the MARGA instrument (provided by Metrohm NL).

| Species | Fixed loop^1^ (µg m^-3^) | Pre-concentration ^2^ (µg m^-3^) |
| --- | --- | --- |
| NH_4_^+^ | 0.05 | 0.005 |
| Na^+^ | 0.05 | 0.005 |
| K^+^ | 0.09 | 0.009 |
| Ca^2+^ | 0.09 | 0.009 |
| Mg^2+^ | 0.06 | 0.006 |
| Cl^-^ | 0.01 | 0.001 |
| NO_3_^-^ | 0.05 | 0.005 |
| SO_4_^2-^ | 0.04 | 0.004 |

^1^The used loop volumes are 250 µL and 500 µL for anions and cations, respectively; ^2^ The used volumes are increased ten times compared to the fixed loop.

**Table S3** Detection limits of the URG-AIM from field tests at North Kensington, London, UK. Manufacturer limit is 0.1 µg m^-3^ for all species.

| Species | Detection limit (µg m^-3^) |
| --- | --- |
| Cl^-^ | 0.06 |
| NO_3_^-^ | 0.04 |
| SO_4_^2-^ | 0.02 |
| Na^+^ | 0.05 |
| NH_4_^+^ | 0.02 |
| K^+^ | 0.02 |
| Mg^2+^ | 0.04 |
| Ca^2+^ | 0.08 |

**Table S4** Detection limits of the Aerosol Chemical Speciation Monitor (ACSM) and the High-Resolution Time-of-Flight Mass Spectrometer (HR-ToF-AMS) instruments (provided by the manufacturer).

| Species | Detection limit (µg m^-3^) | |
| --- | --- | --- |
|  | ACSM ^1^ | HR- ToF-ACSM ^2^ |
| NH_4_^+^ | 0.5 | 0.06 |
| Cl^-^ | 0.02 | 0.003 |
| NO_3_^-^ | 0.02 | 0.007 |
| SO_4_^2-^ | 0.04 | 0.006 |

^1^ Calculated over 30 minutes and taking 3σ; ^2^calculated over 10 minutes

Equivalence calculations for species.

Below are the final figures of the orthogonal regression used to calculate the expanded uncertainty for each species in each case study. It is stated with each figure if the CM has been corrected. Refer to Tables 5 to 7 in the main text for further details.

1. NH_4_^+^


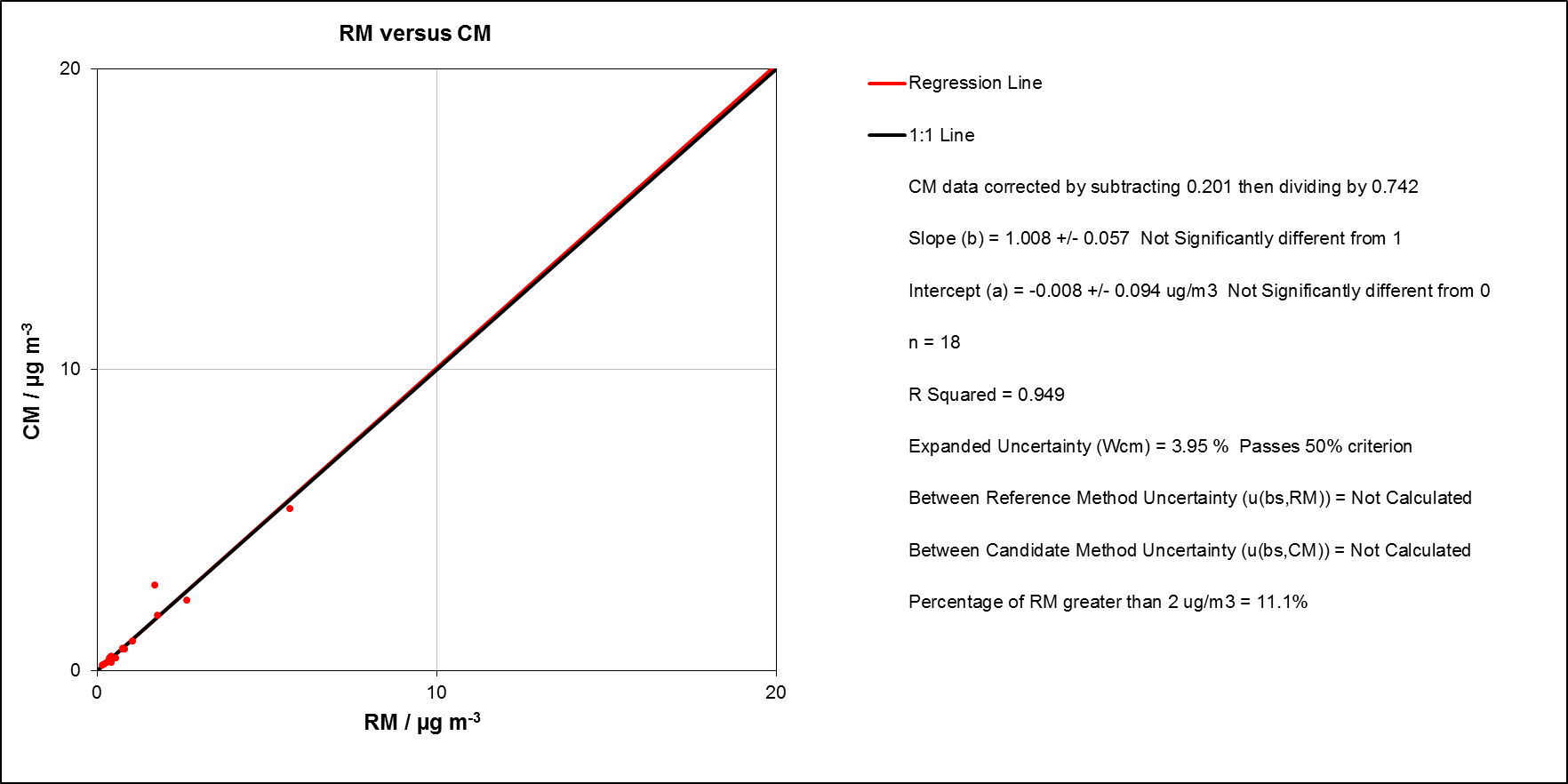


Figure S1 NH_4_^+^, ACSM (PM_1_) vs RM (PM_2.5_), Revin, France


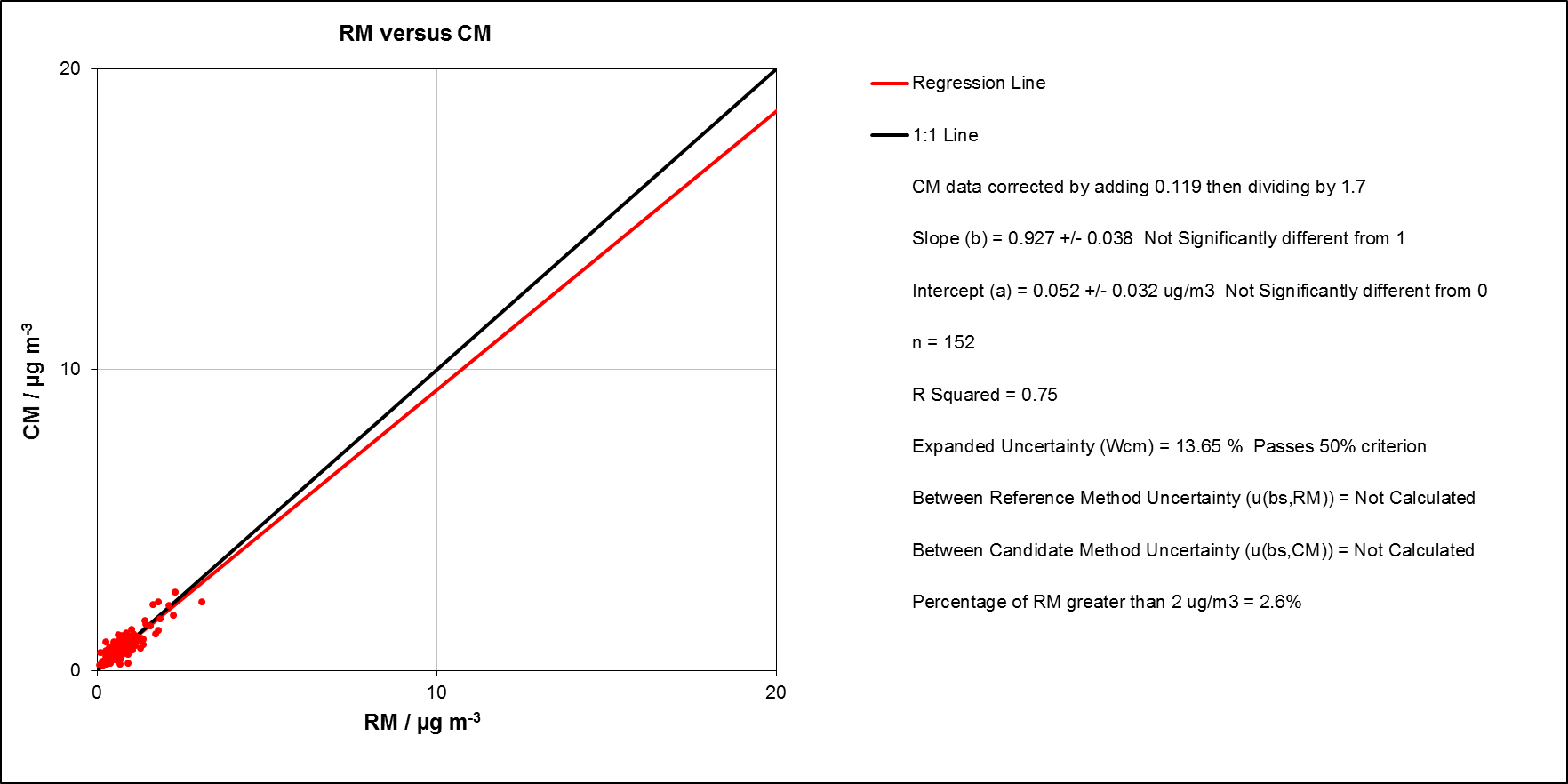


Figure S2, NH_4_^+^, ACSM (PM_1_) vs RM (PM_1_), Barcelona, Spain


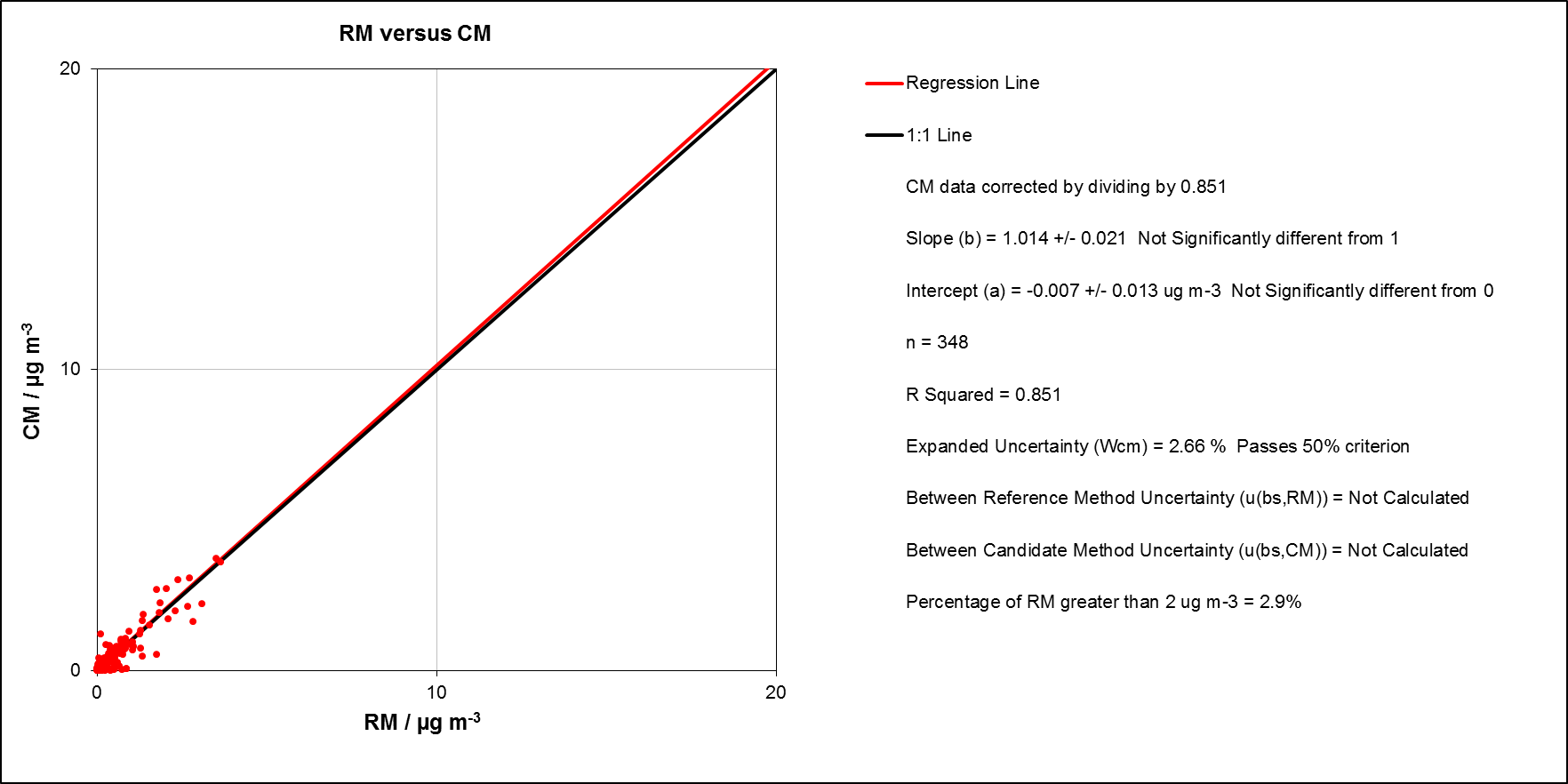


Figure S3, NH_4_^+^, HR-TOF-AMS (PM_1_) vs RM (PM_2.5_), Mace Head, Ireland.


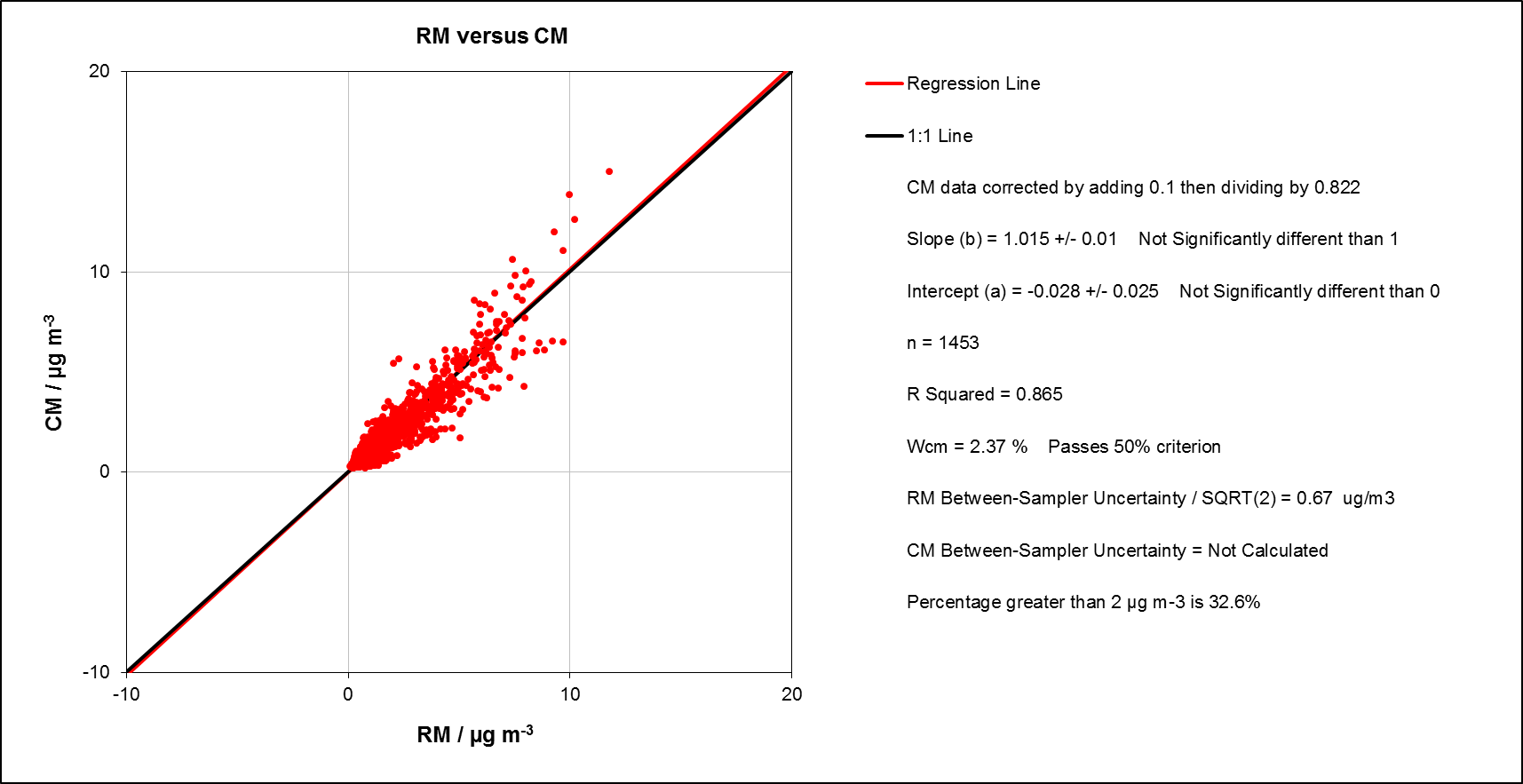


Figure S4, NH_4_^+^, MARGA (PM_10_) vs RM (PM_10_), Melpitz, Germany.


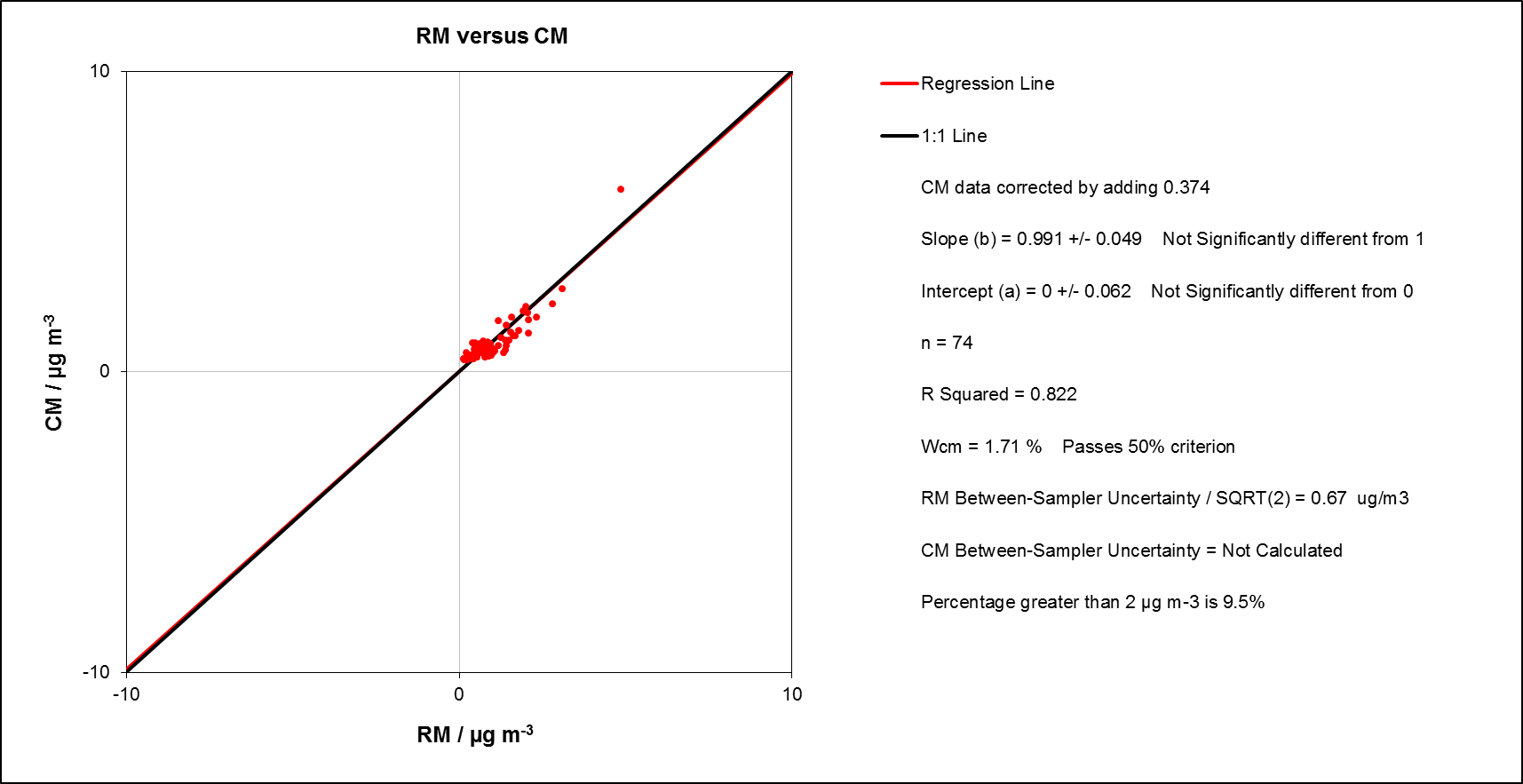


Figure S5, NH_4_^+^, MARGA (PM_10_) vs RM (PM_10_), Kumpula, Finland.


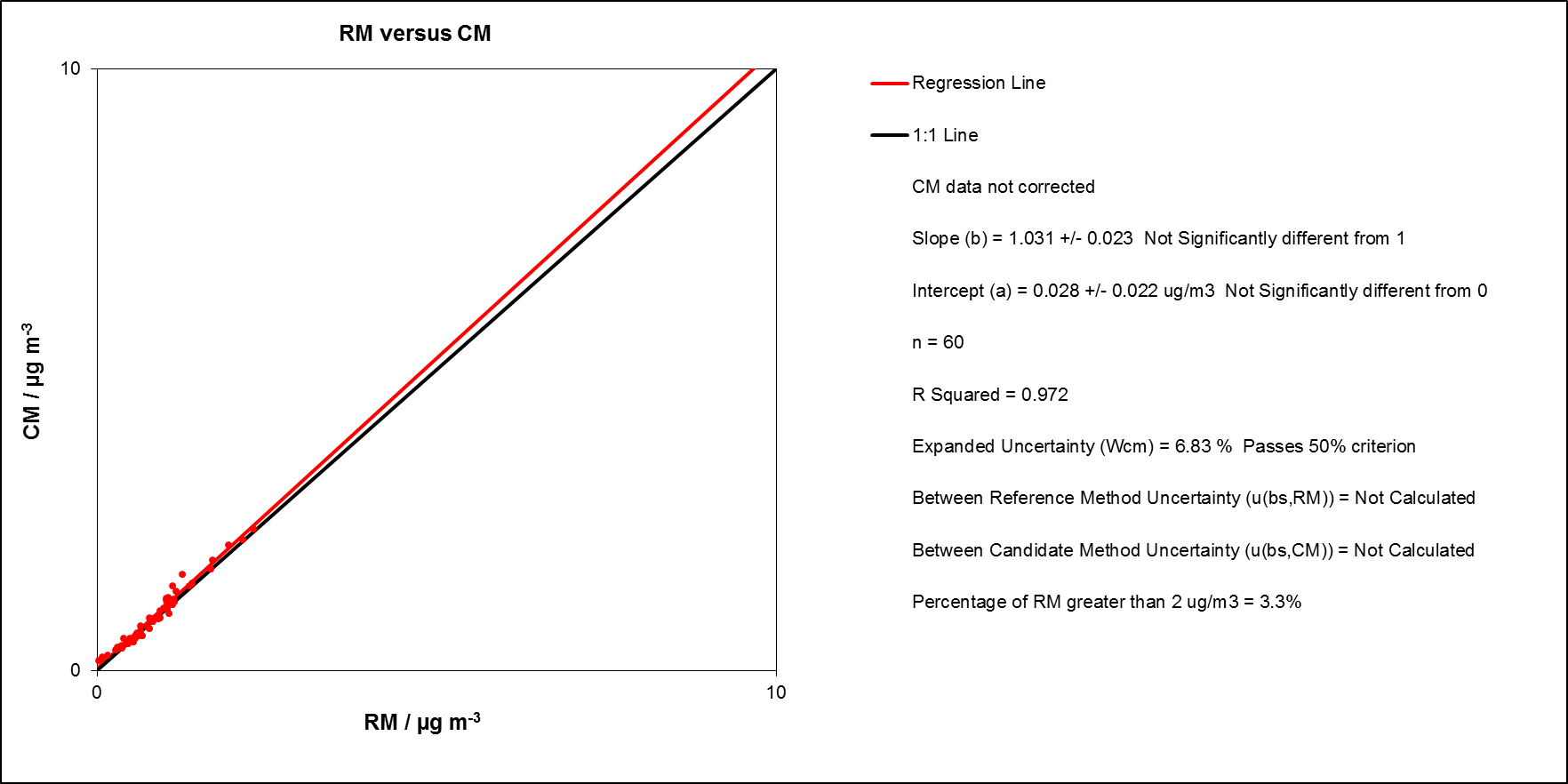


Figure S6a, NH_4_^+^, MARGA CM1 (~PM_26_) vs RM (PM_2.5_), Research Triangle Park, United States.


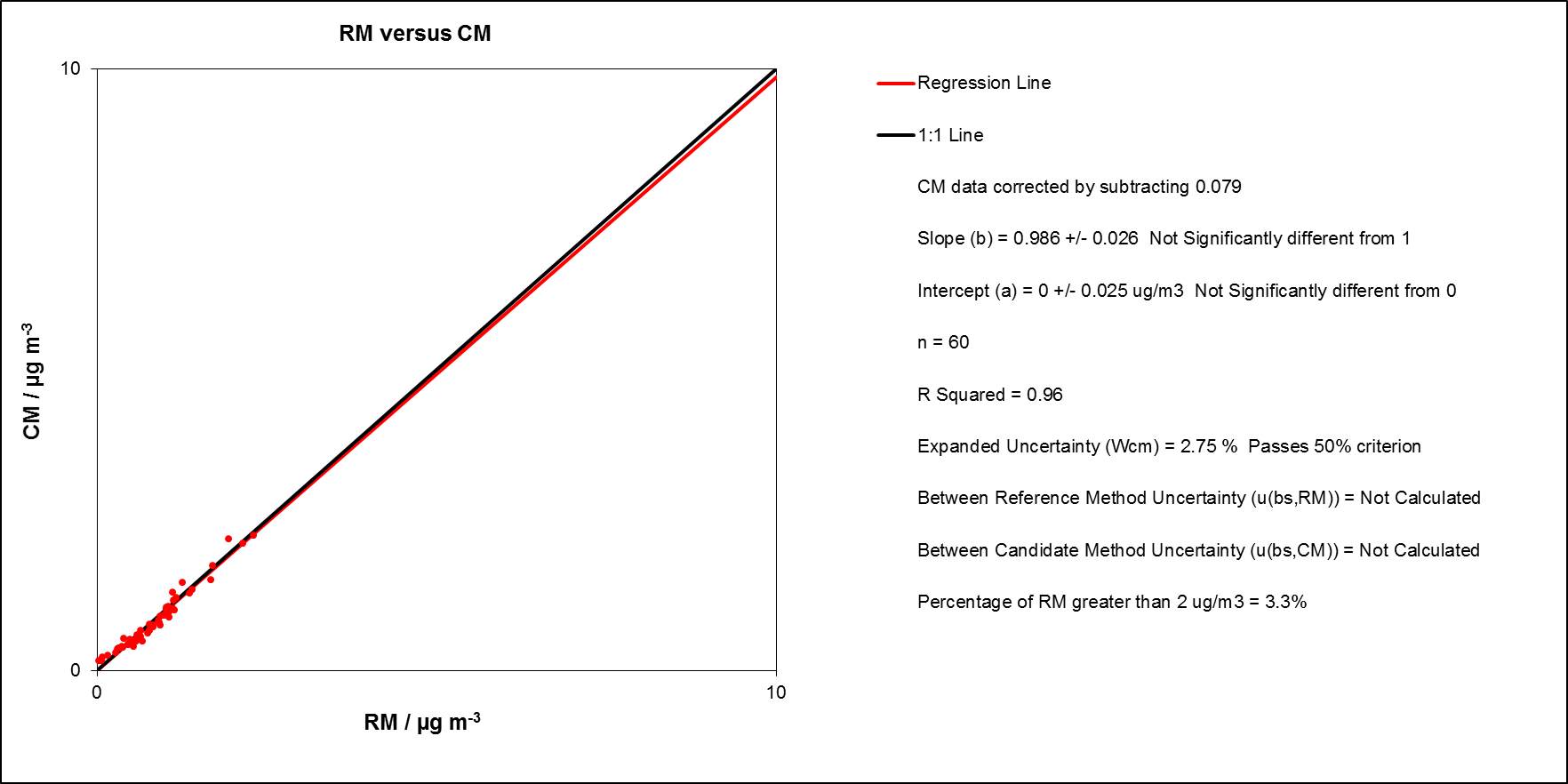


Figure S6b, NH_4_^+^, MARGA CM2 (~PM_26_) vs RM (PM_2.5_), Research Triangle Park, United States.
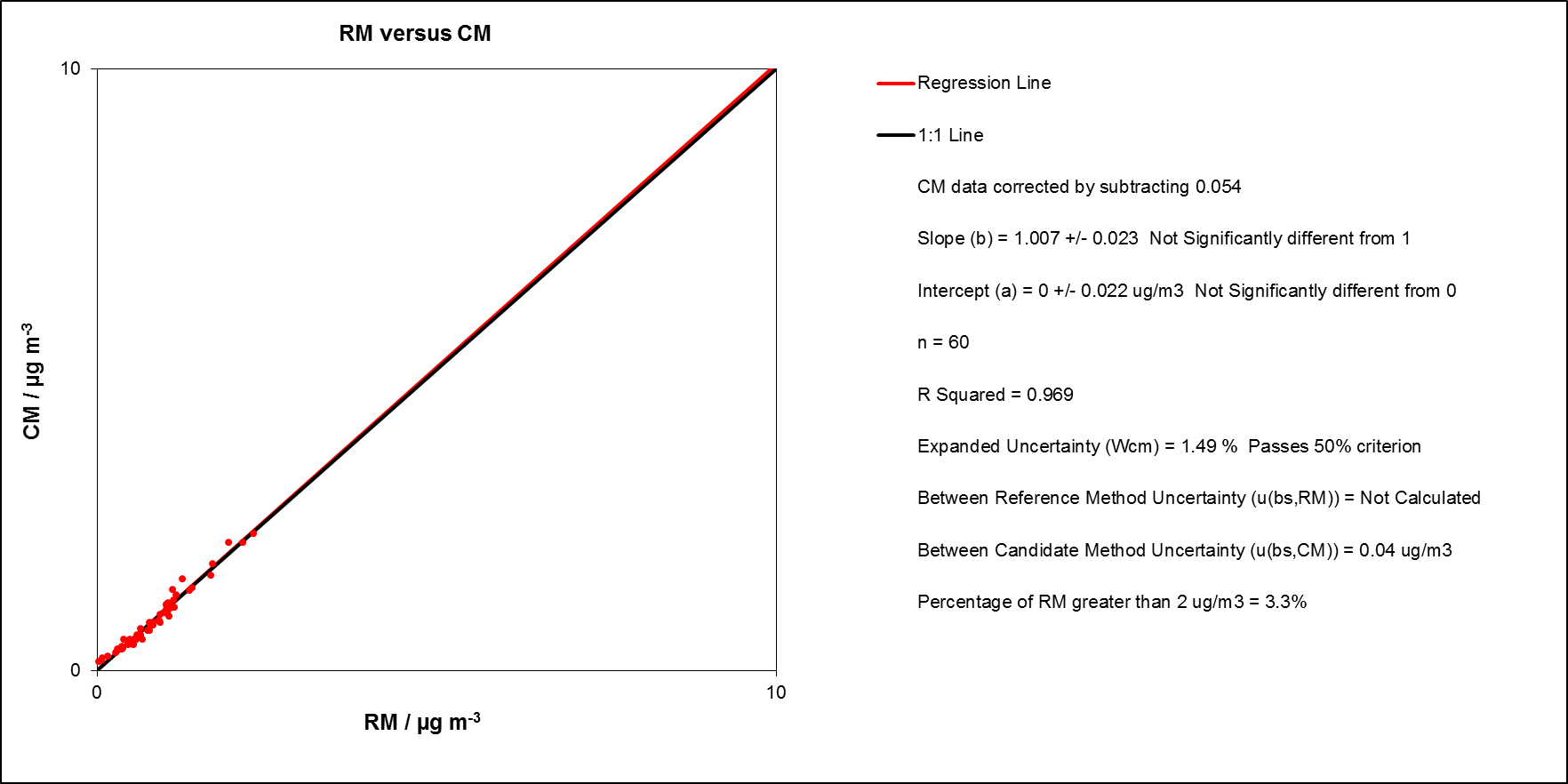


Figure S6c, NH_4_^+^, averaged MARGA (~PM_26_) vs RM (PM_2.5_), Research Triangle Park, United States.


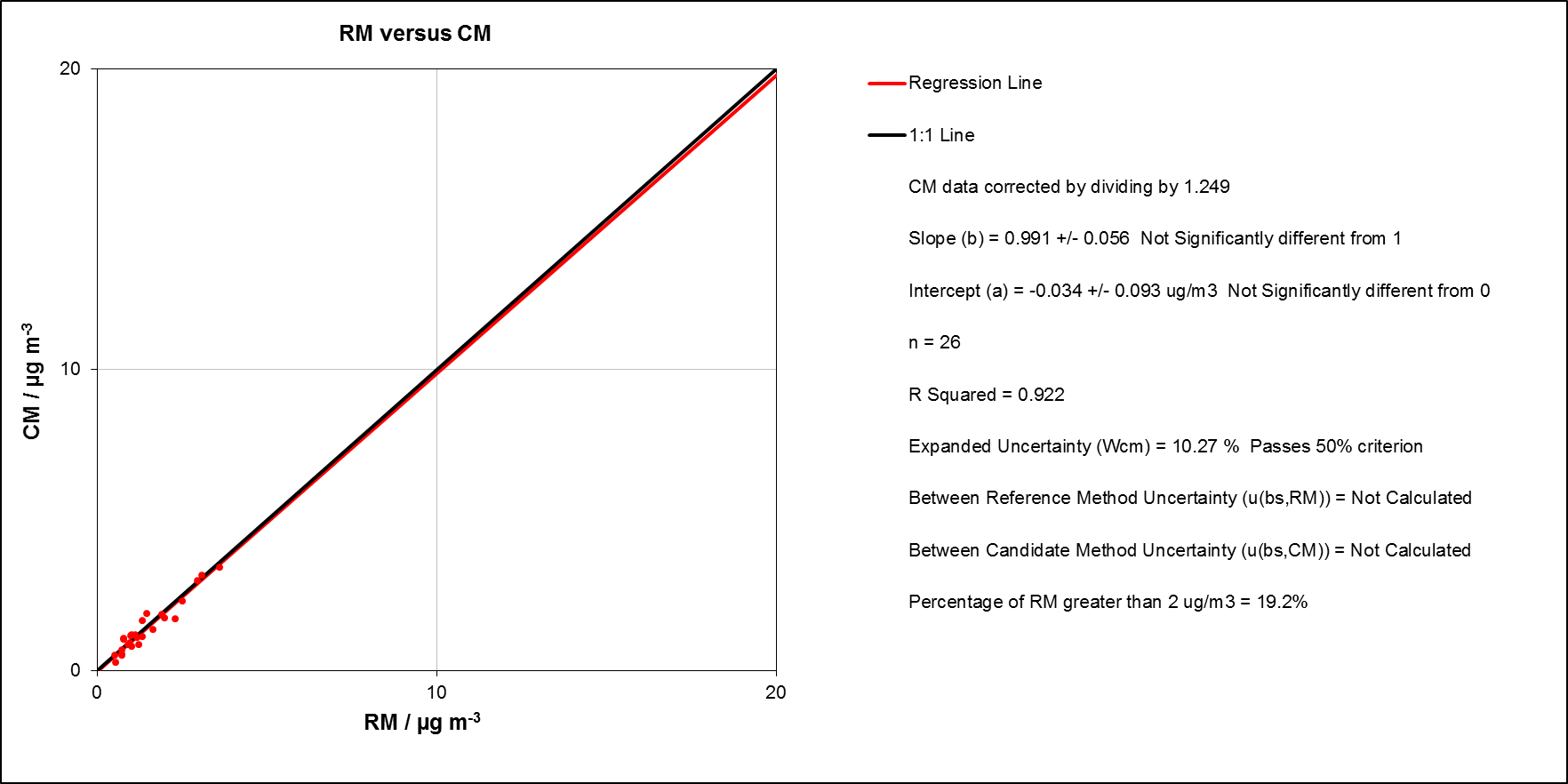


Figure S7, NH_4_^+^, MARGA (PM_1_) vs RM (PM_1_), San Pietro Capofiume, Italy


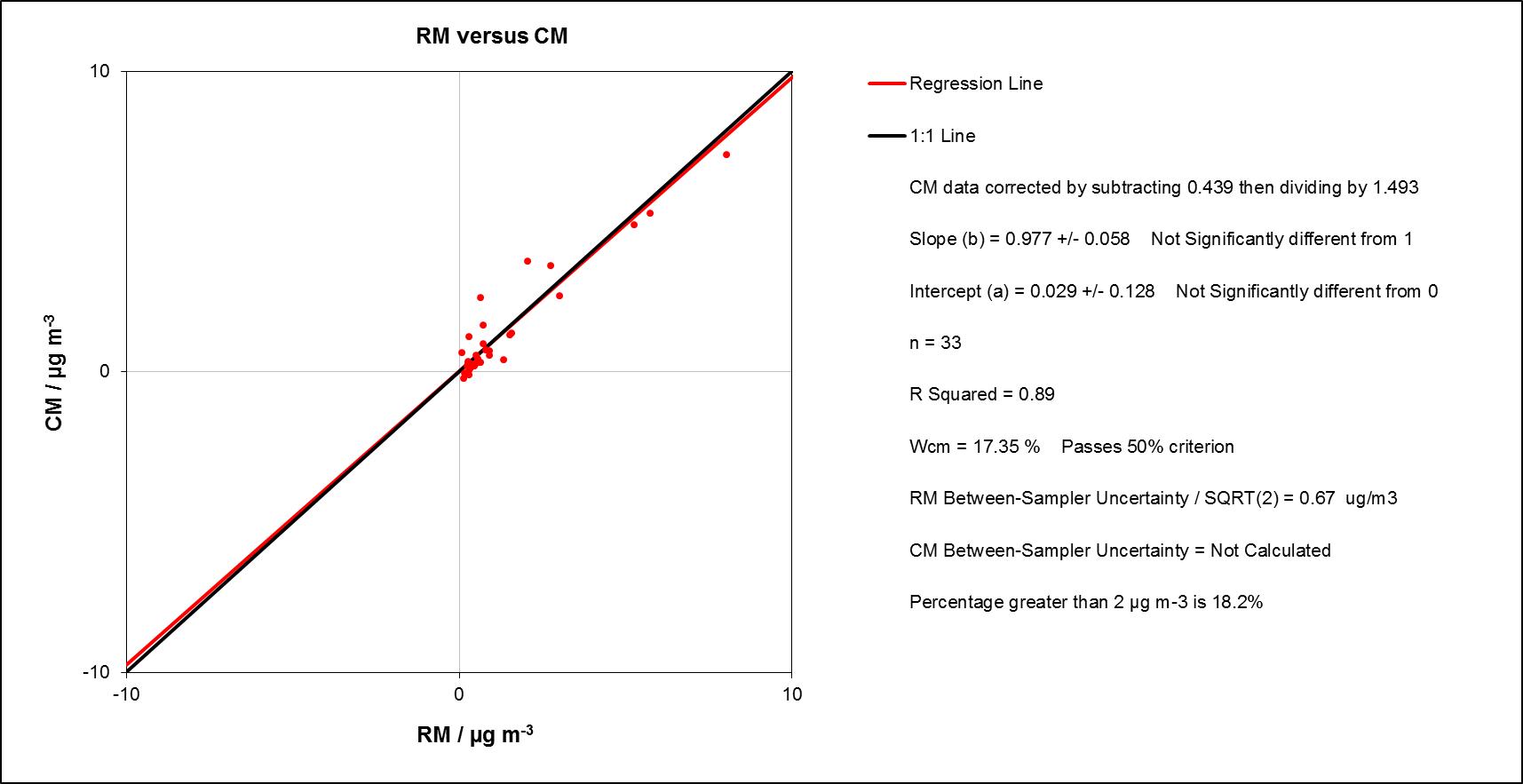


Figure S8, NH_4_^+^, AIM (PM_10_) vs RM (PM_10_), North Kensington, UK.

1. NO_3_^-^


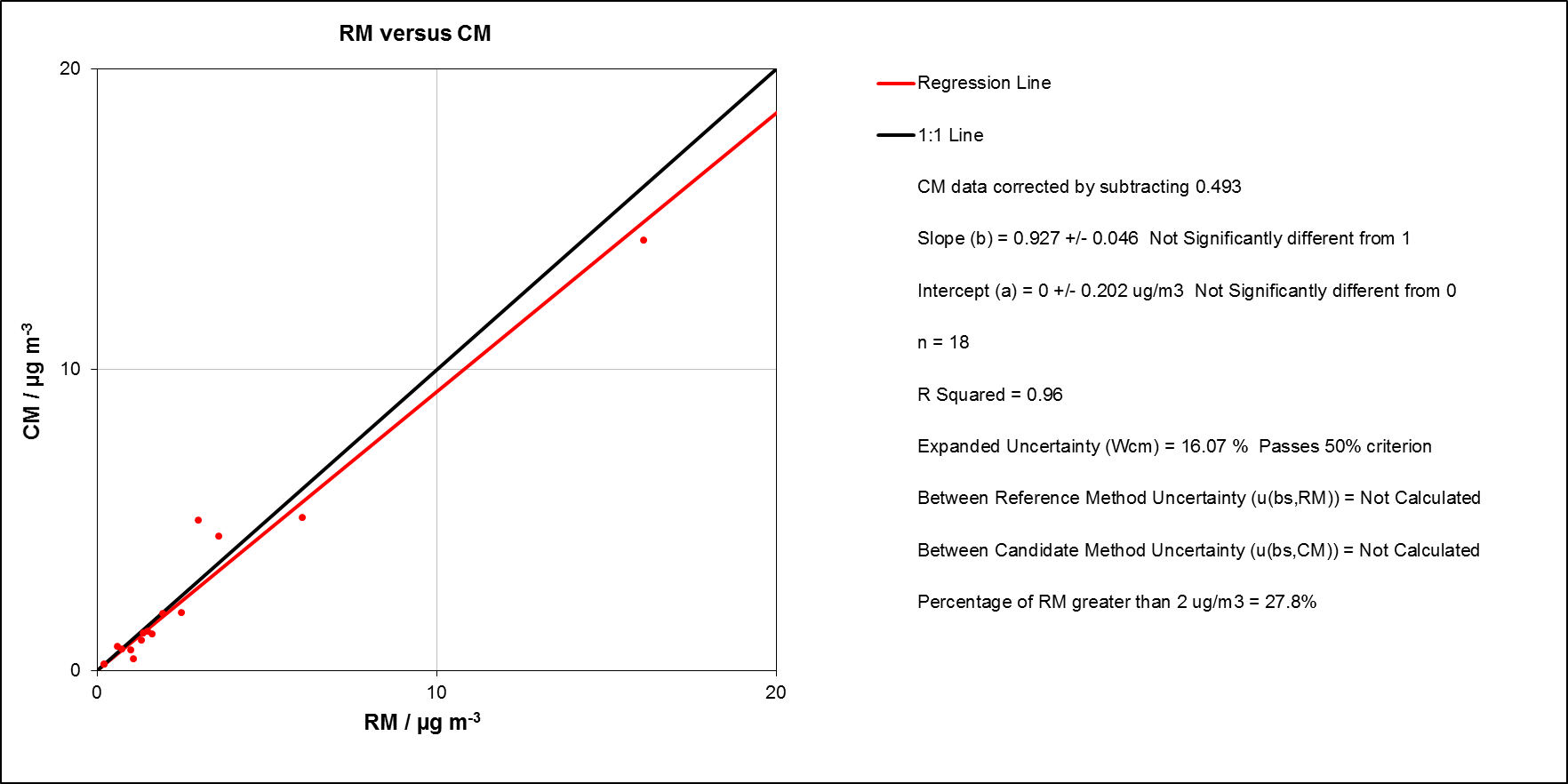


Figure S9, NO_3_^-^, ACSM (PM_1_) vs RM (PM_2.5_), Revin, France


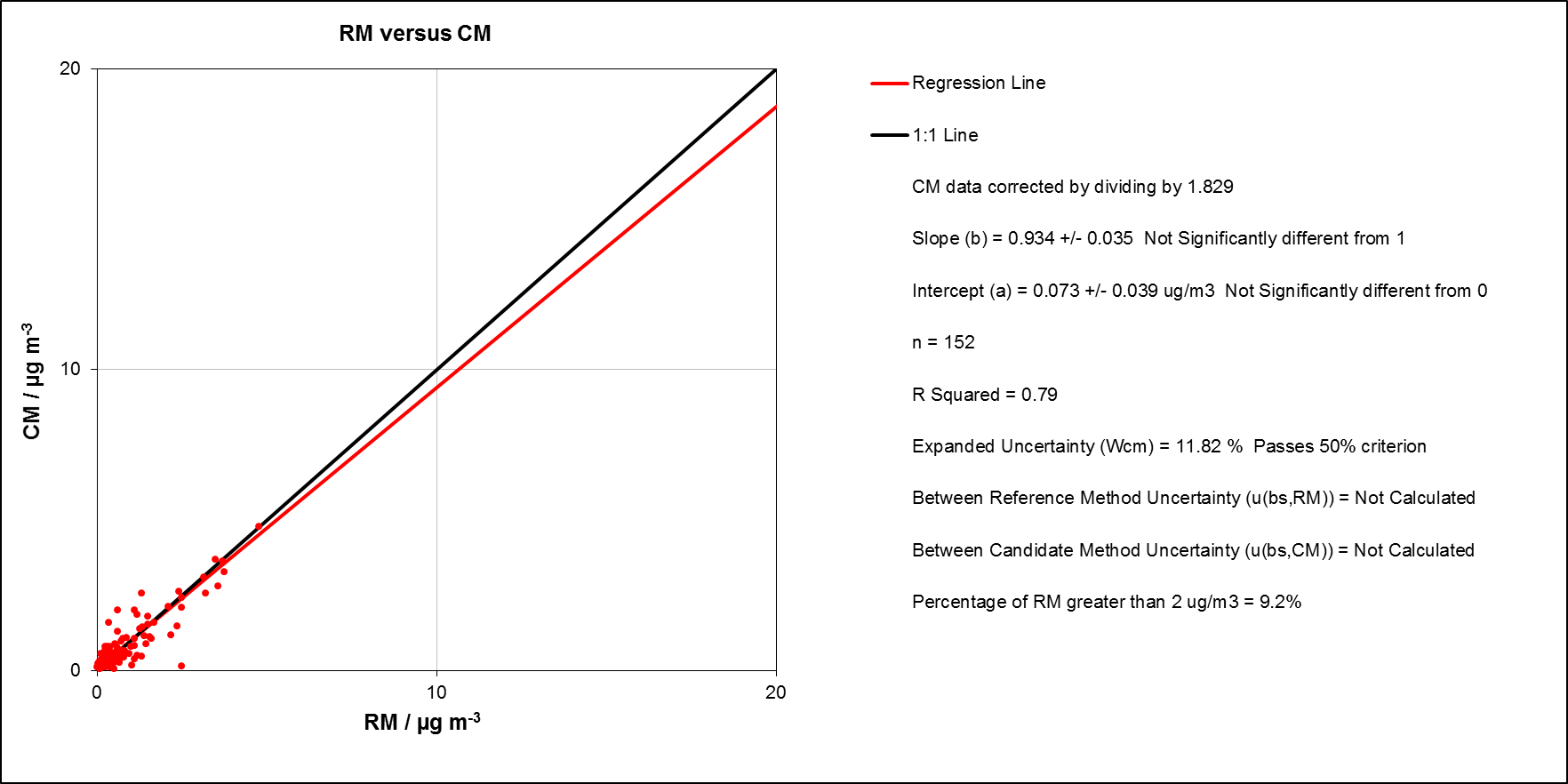


Figure S10, NO_3_^-^, ACSM (PM_1_) vs RM (PM_1_), Barcelona, Spain


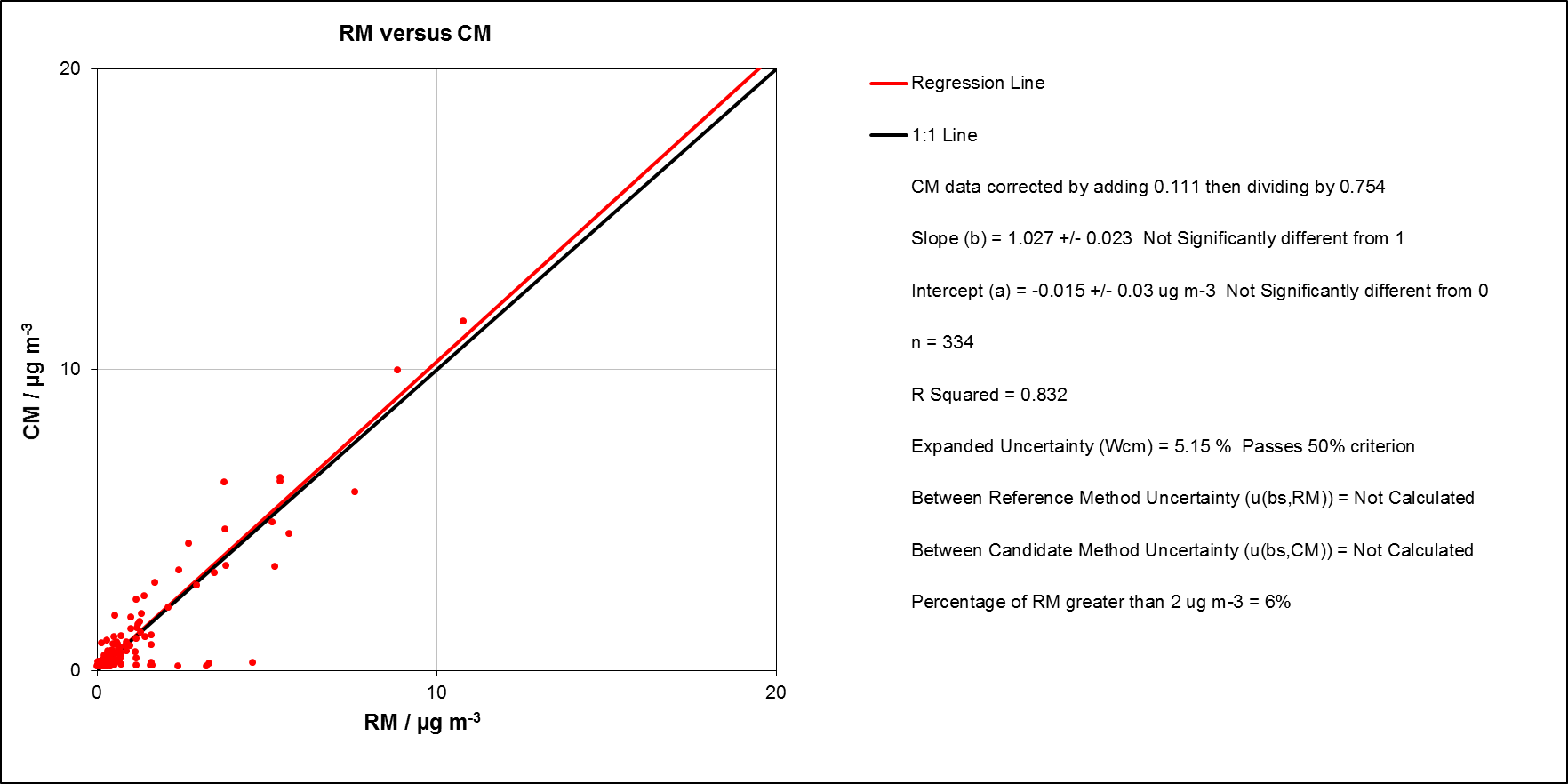


Figure S11, NO_3_^-^, HR-TOF-AMS (PM_1_) vs RM (PM_2.5_), Mace Head, Ireland.


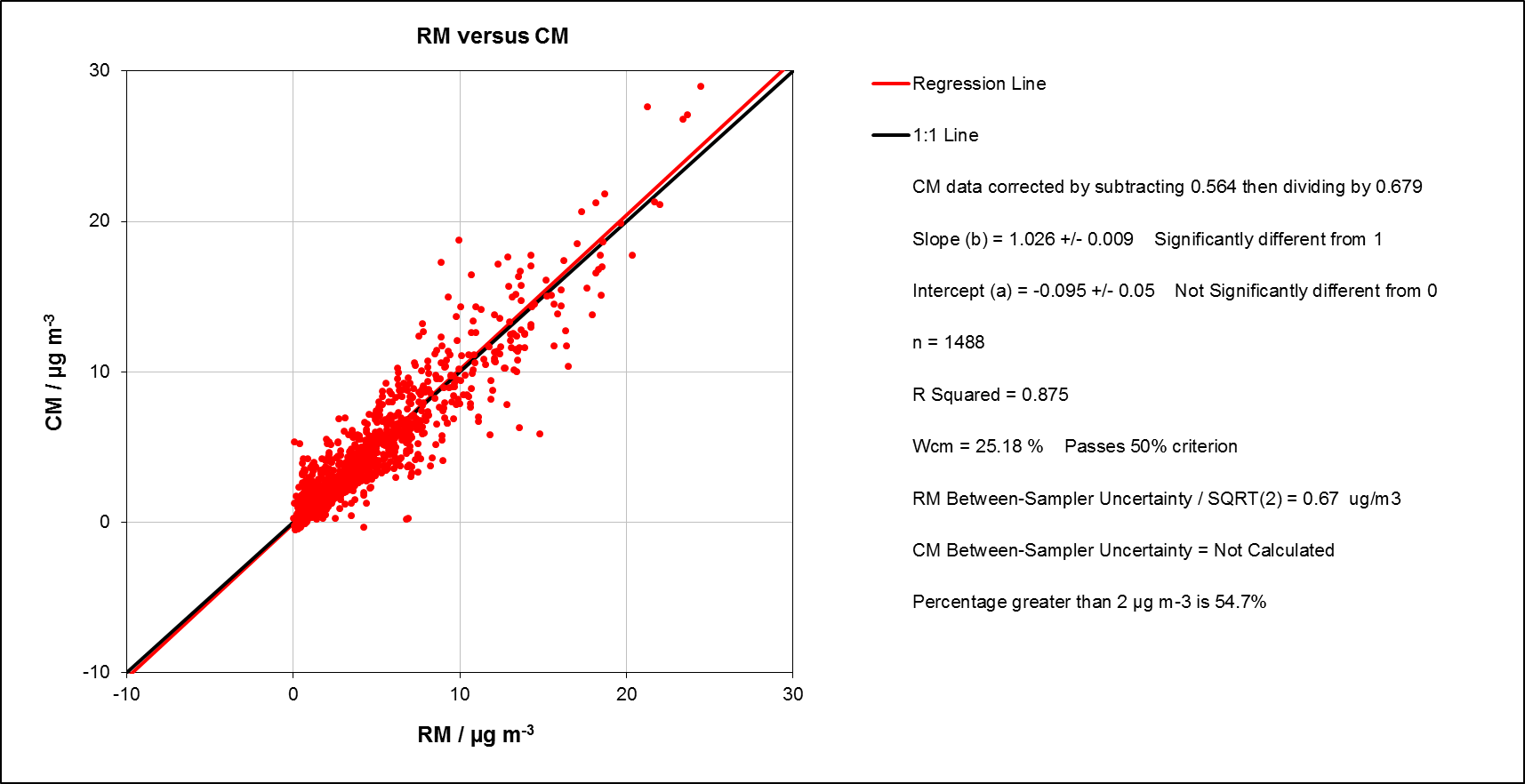


Figure S12, NO_3_^-^, MARGA (PM_10_) vs RM (PM_10_), Melpitz, Germany.


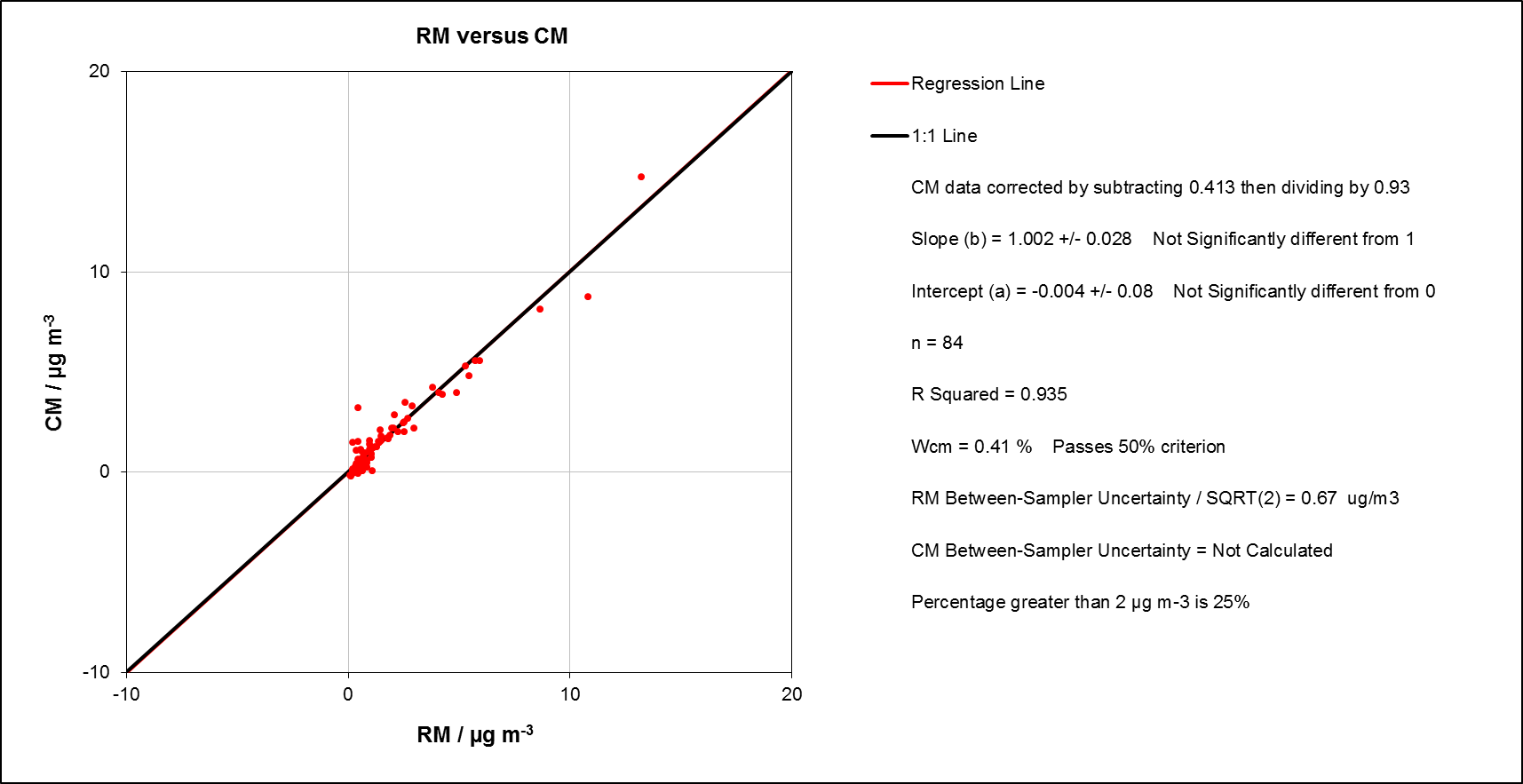


Figure S13, NO_3_^-^, MARGA (PM_10_) vs RM (PM_10_), Kumpula, Finland.


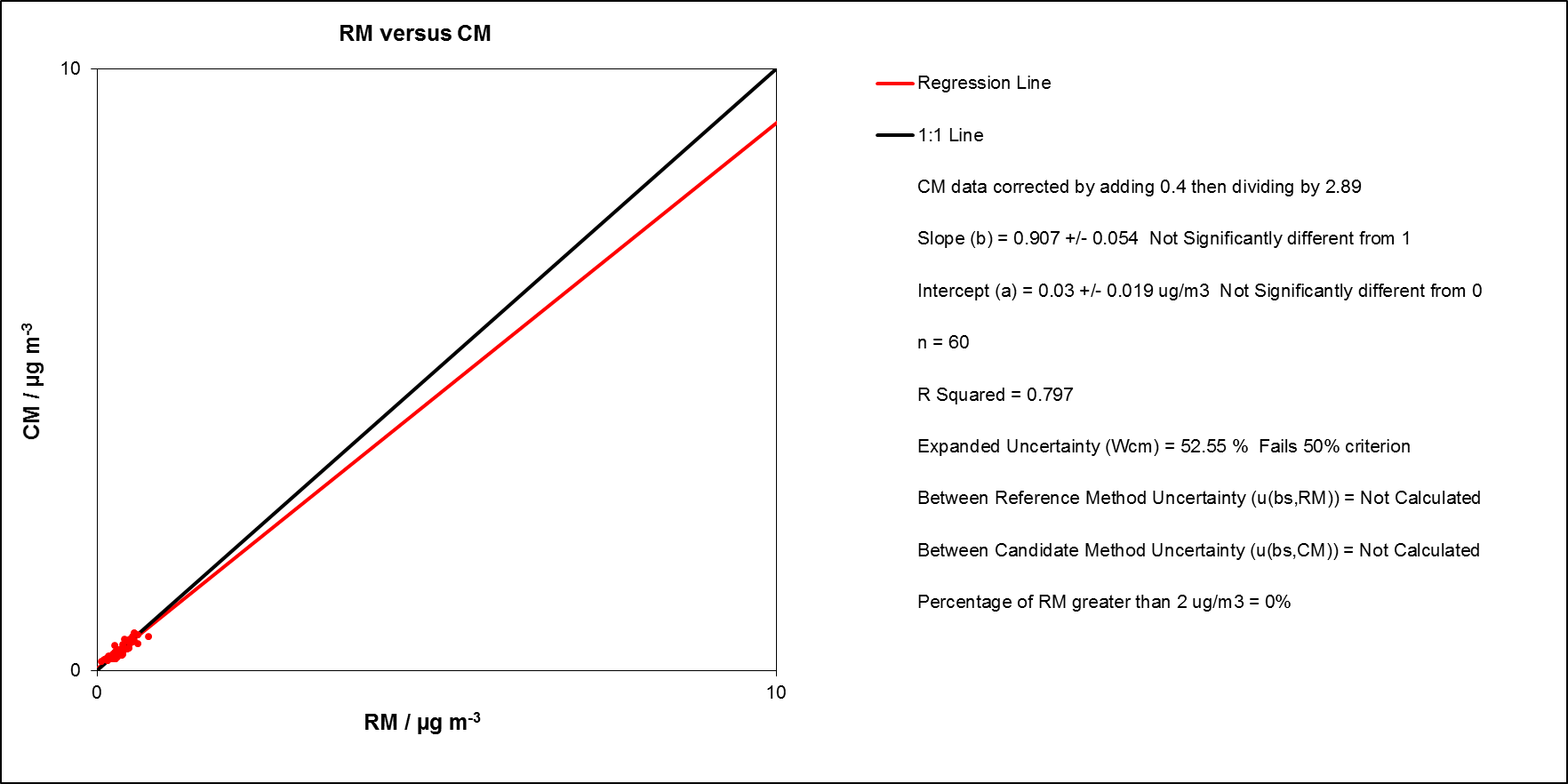


Figure S14a, NO_3_^-^, MARGA CM1 (~PM_26_) vs RM (PM_2.5_), Research Triangle Park, United States.


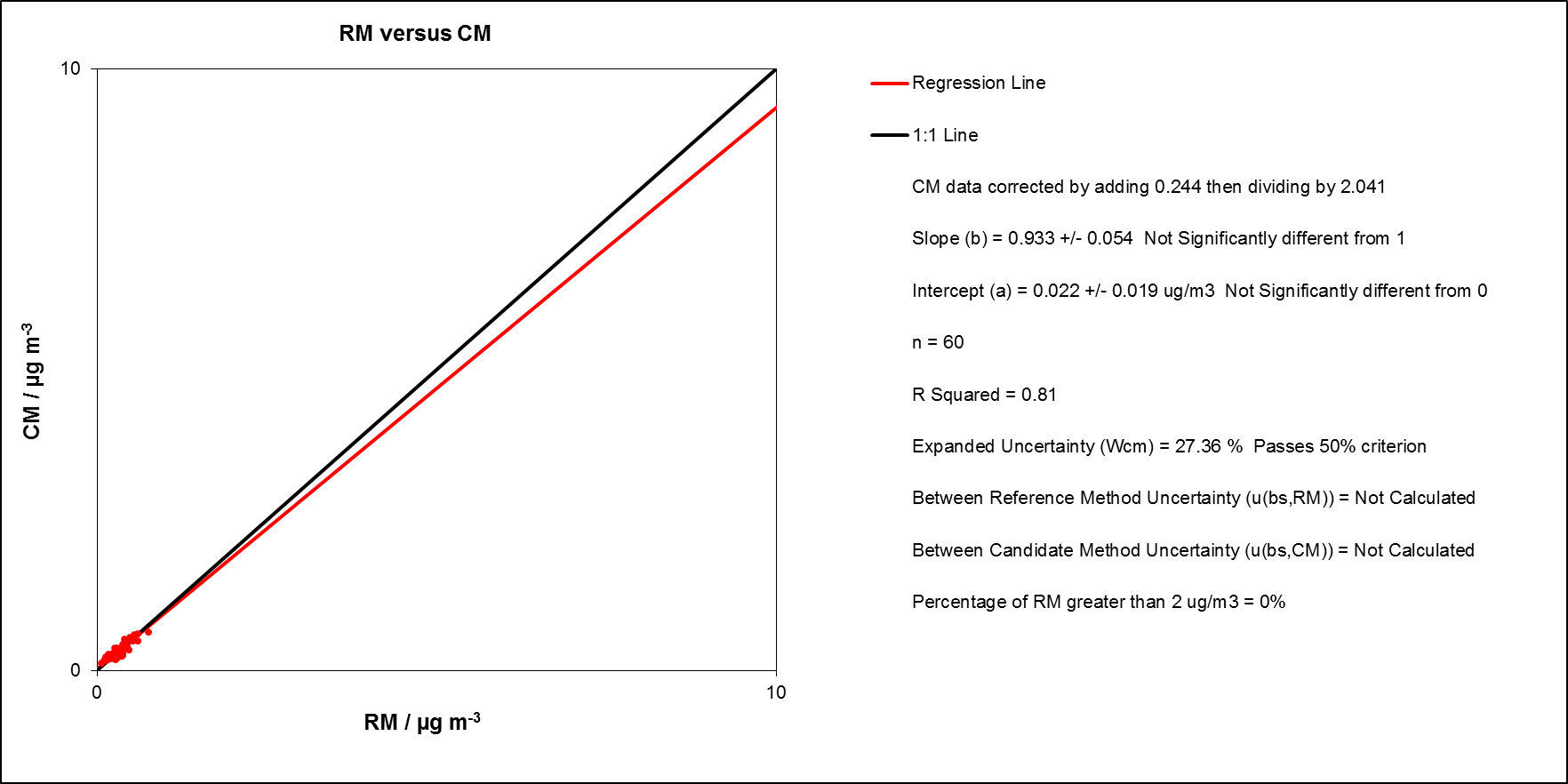


Figure S14b, NO_3_^-^, MARGA CM2 (~PM_26_) vs RM (PM_2.5_), Research Triangle Park, United States.


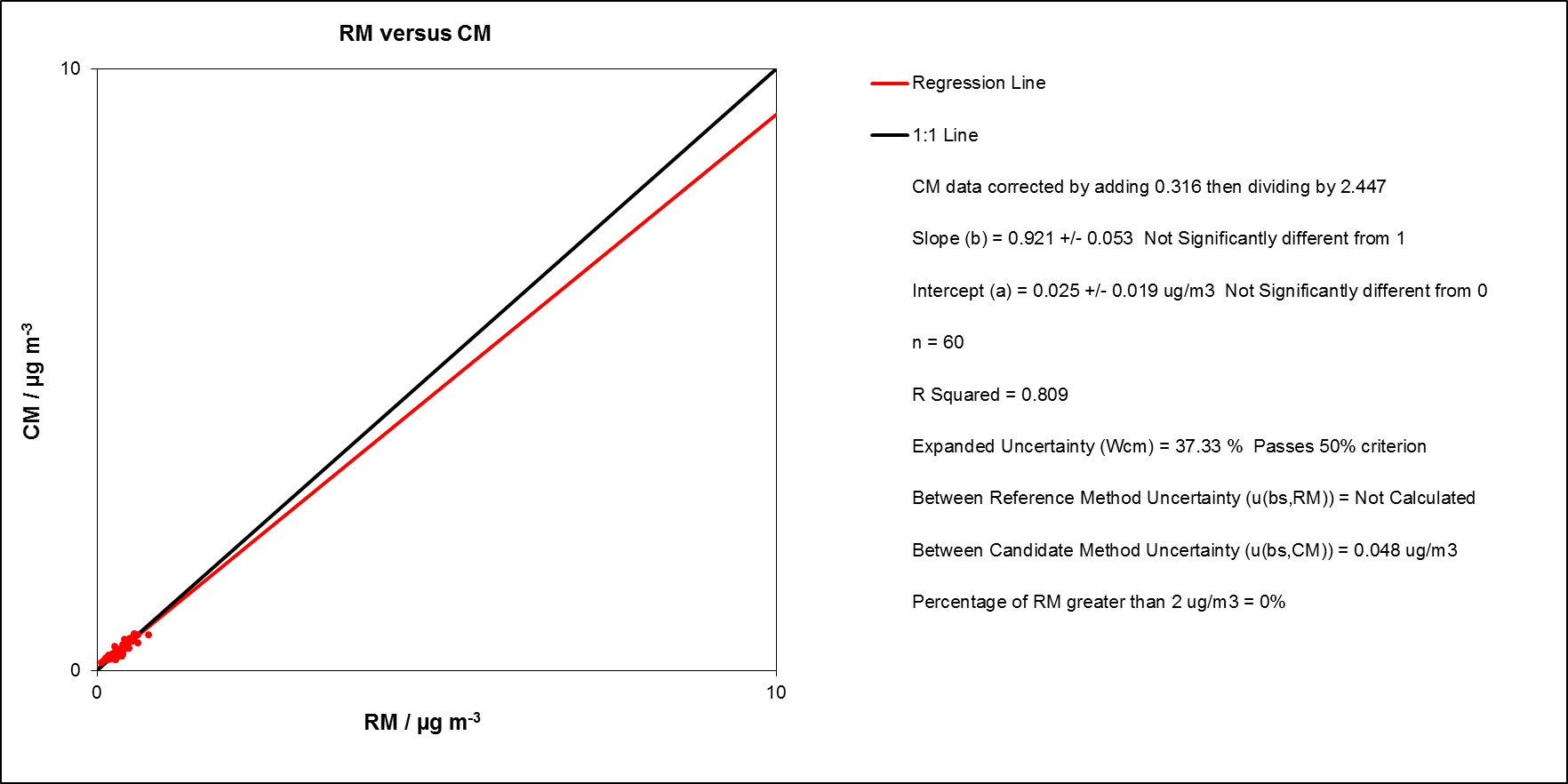


Figure S14c, NO_3_^-^, averaged MARGA (~PM_26_) vs RM (PM_2.5_), Research Triangle Park, United States.


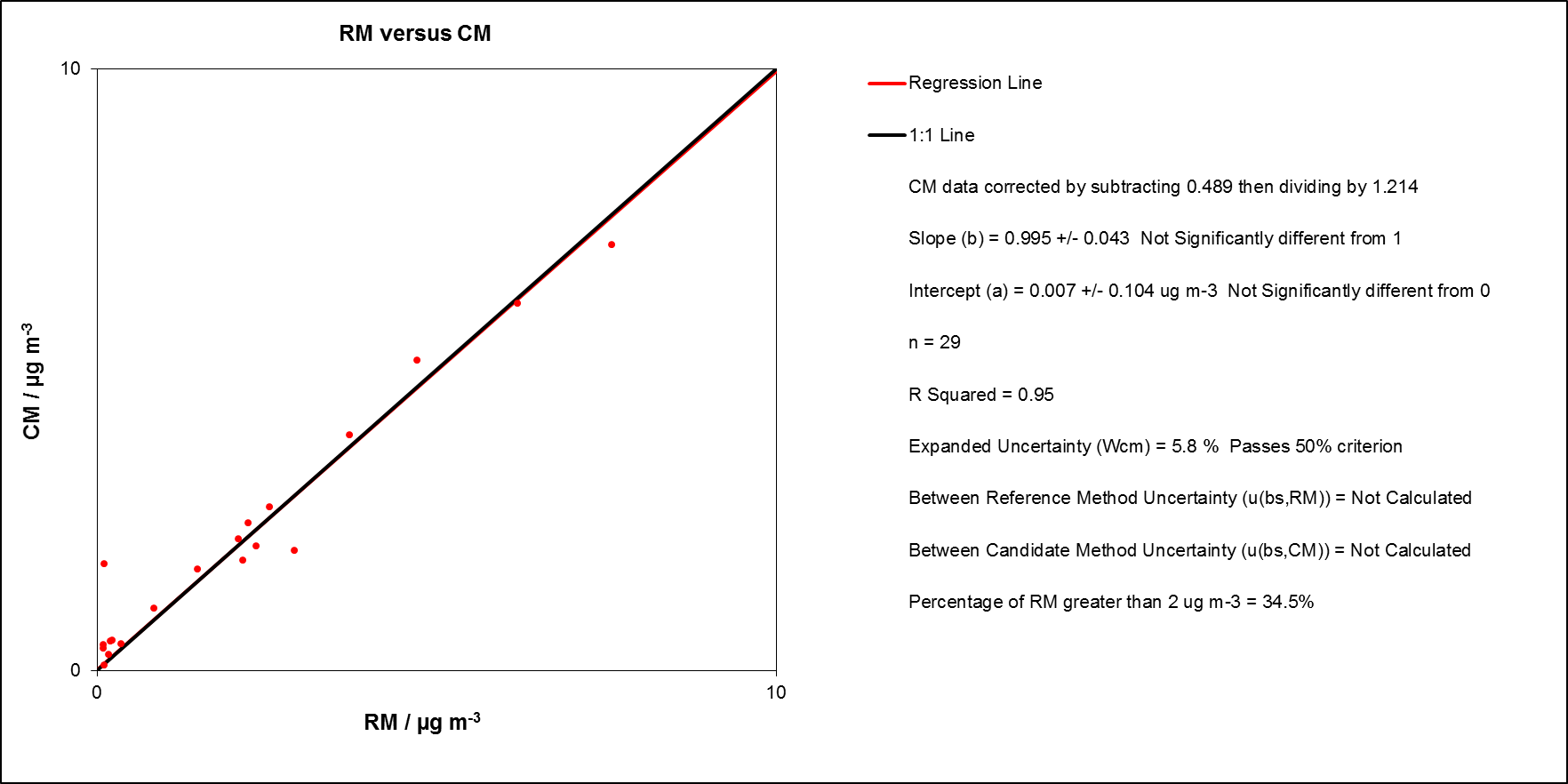


Figure S15, NO_3_^-^, MARGA (PM_1_) vs RM (PM_1_), San Pietro Capofiume, Italy


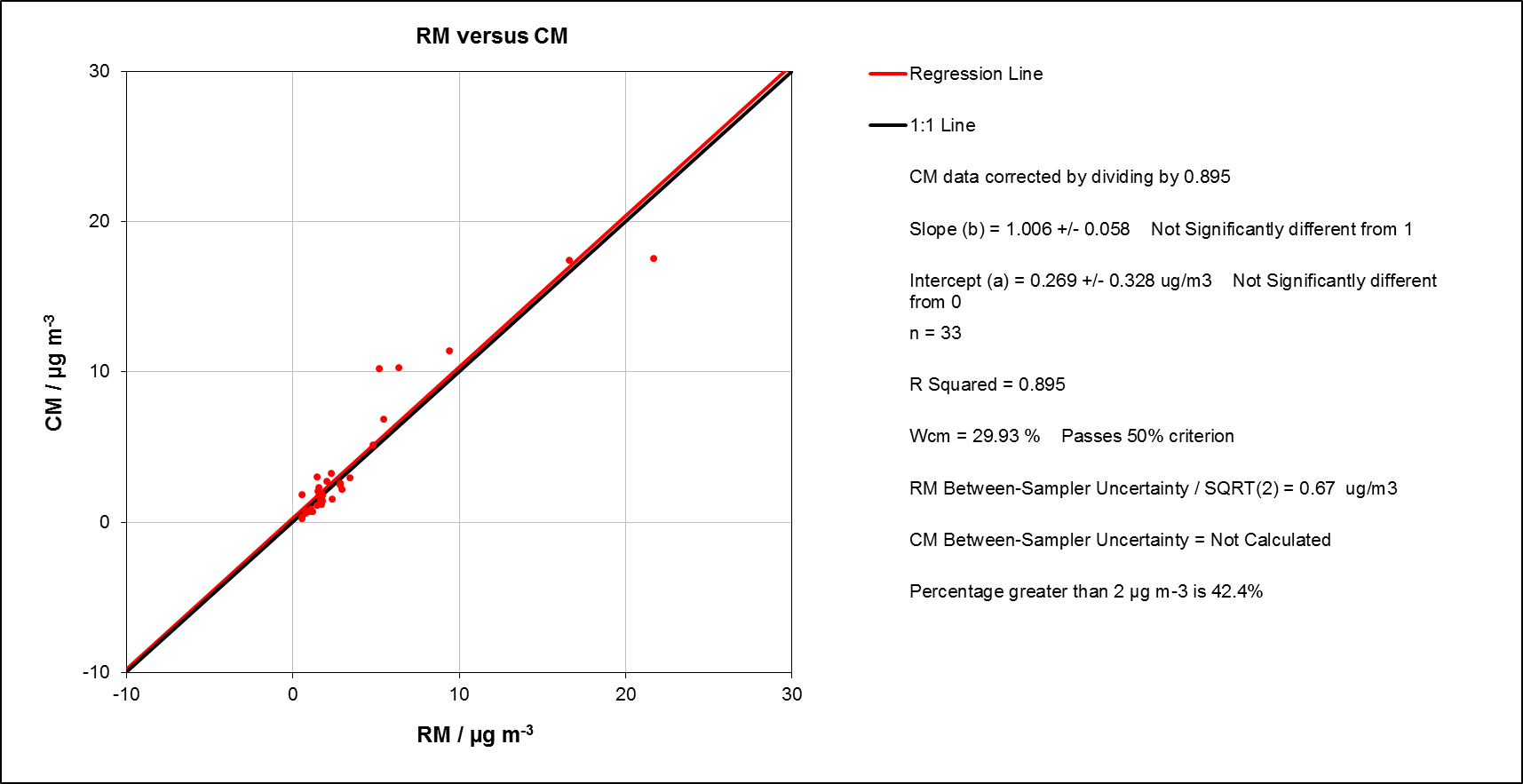
Figure S16, NO_3_^-^, AIM (PM_10_) vs RM (PM_10_), North Kensington, UK.

1. SO_4_^2-^


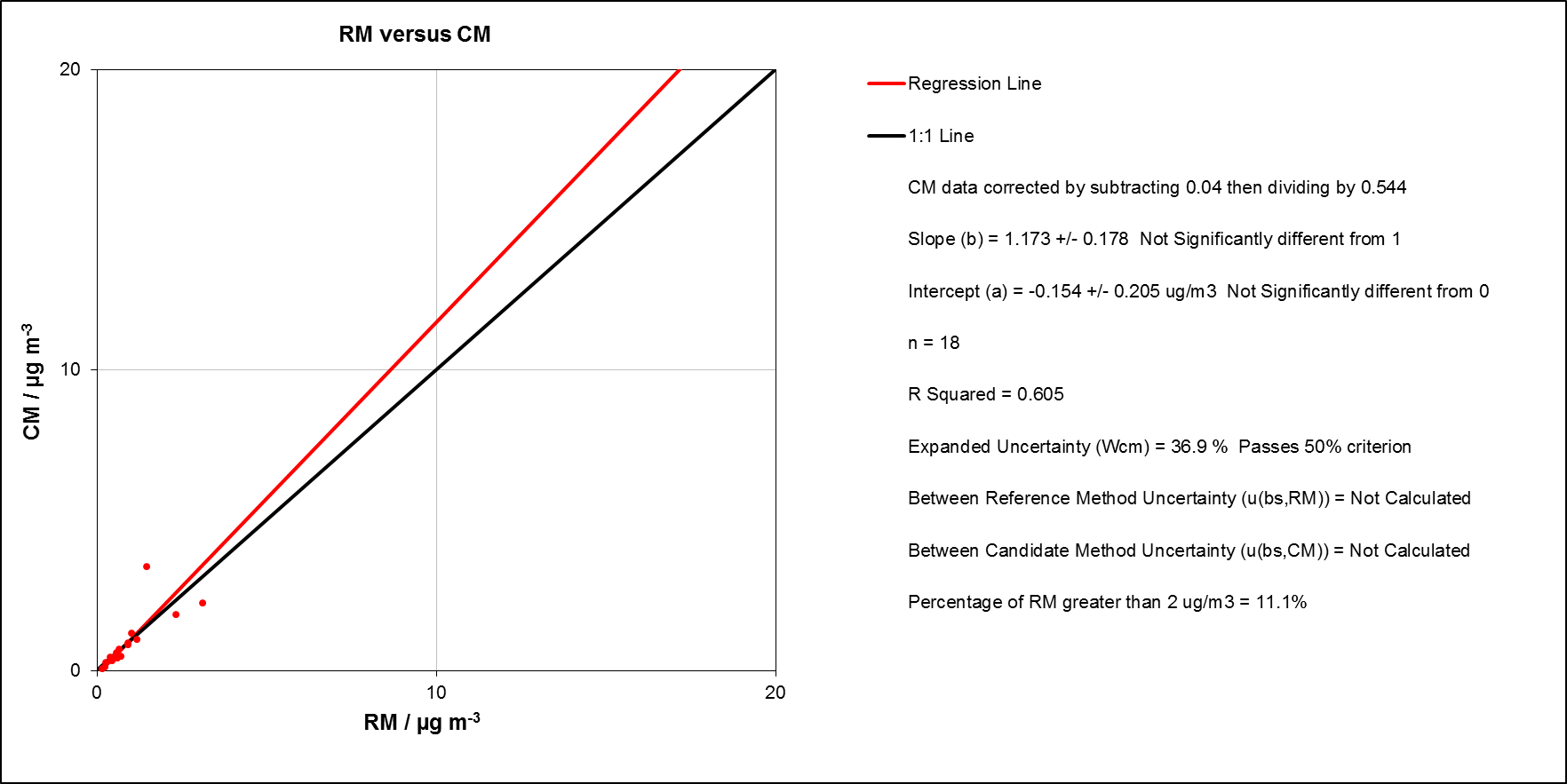


Figure S17, SO_4_^2-^, ACSM (PM_1_) vs RM (PM_2.5_), Revin, France


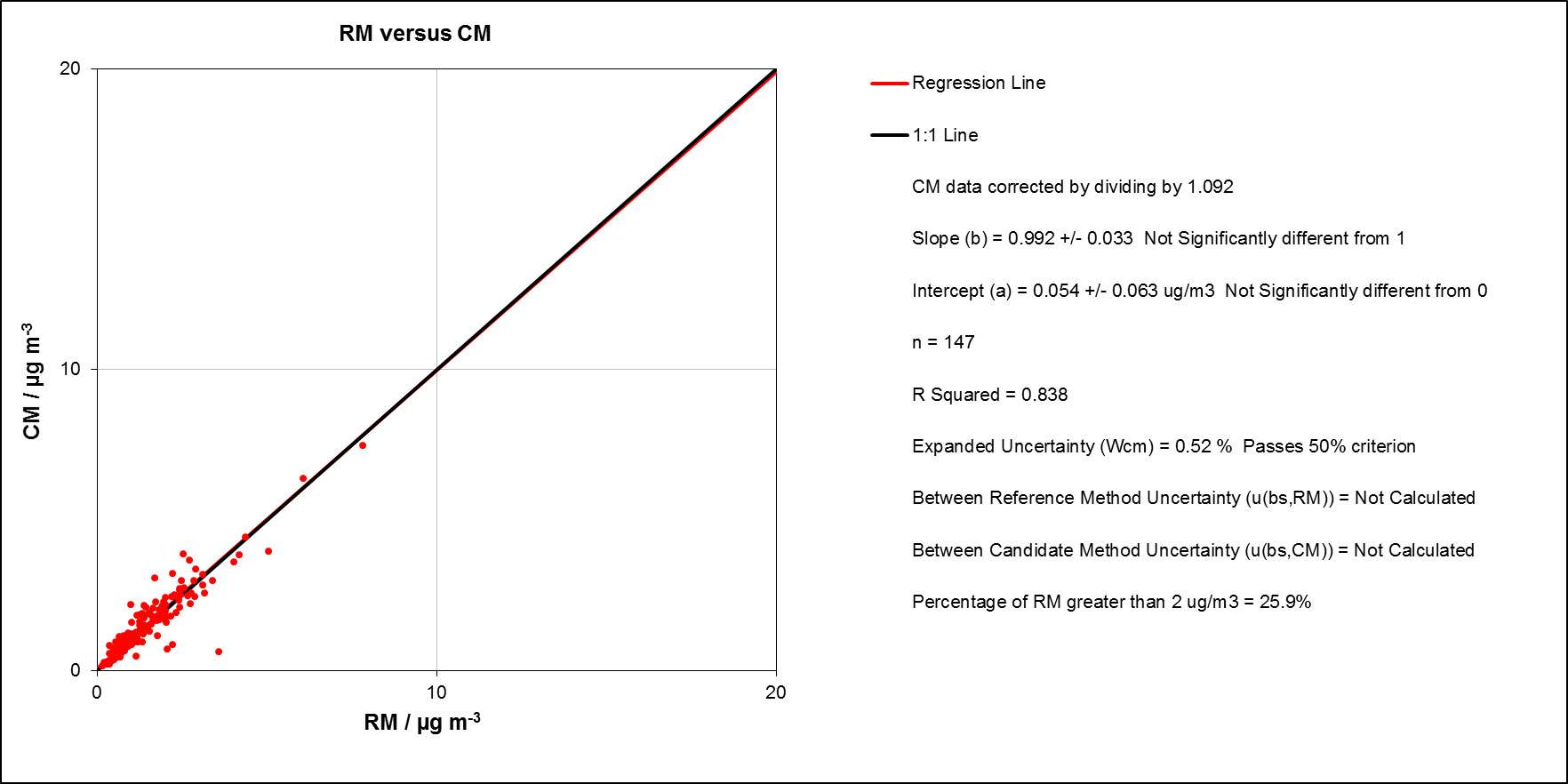


Figure S18, SO_4_^2-^, ACSM (PM_1_) vs RM (PM_1_), Barcelona, Spain


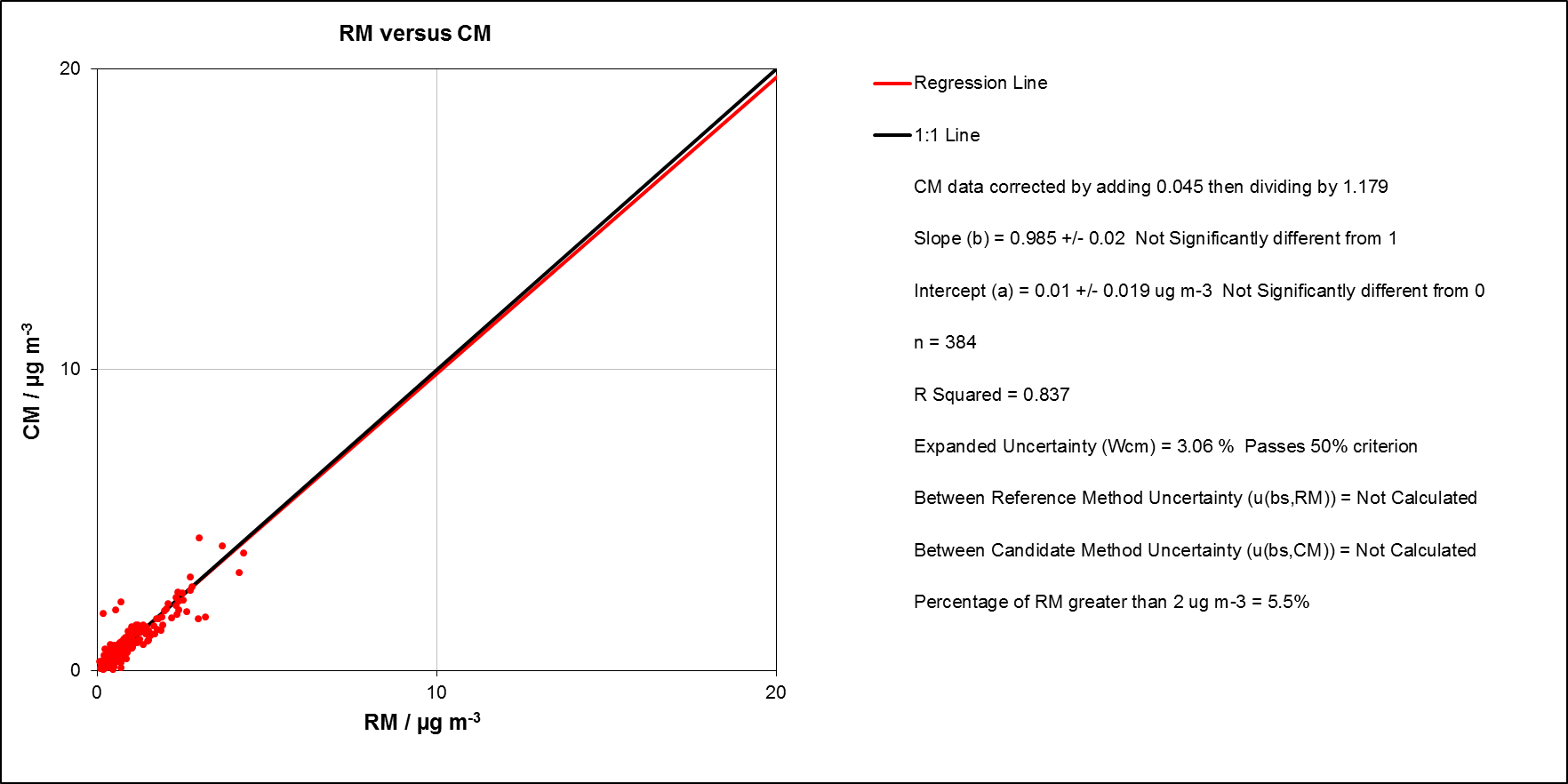


Figure S19, SO_4_^2-^, HR-TOF-AMS (PM_1_) vs RM (PM_2.5_), Mace Head, Ireland.


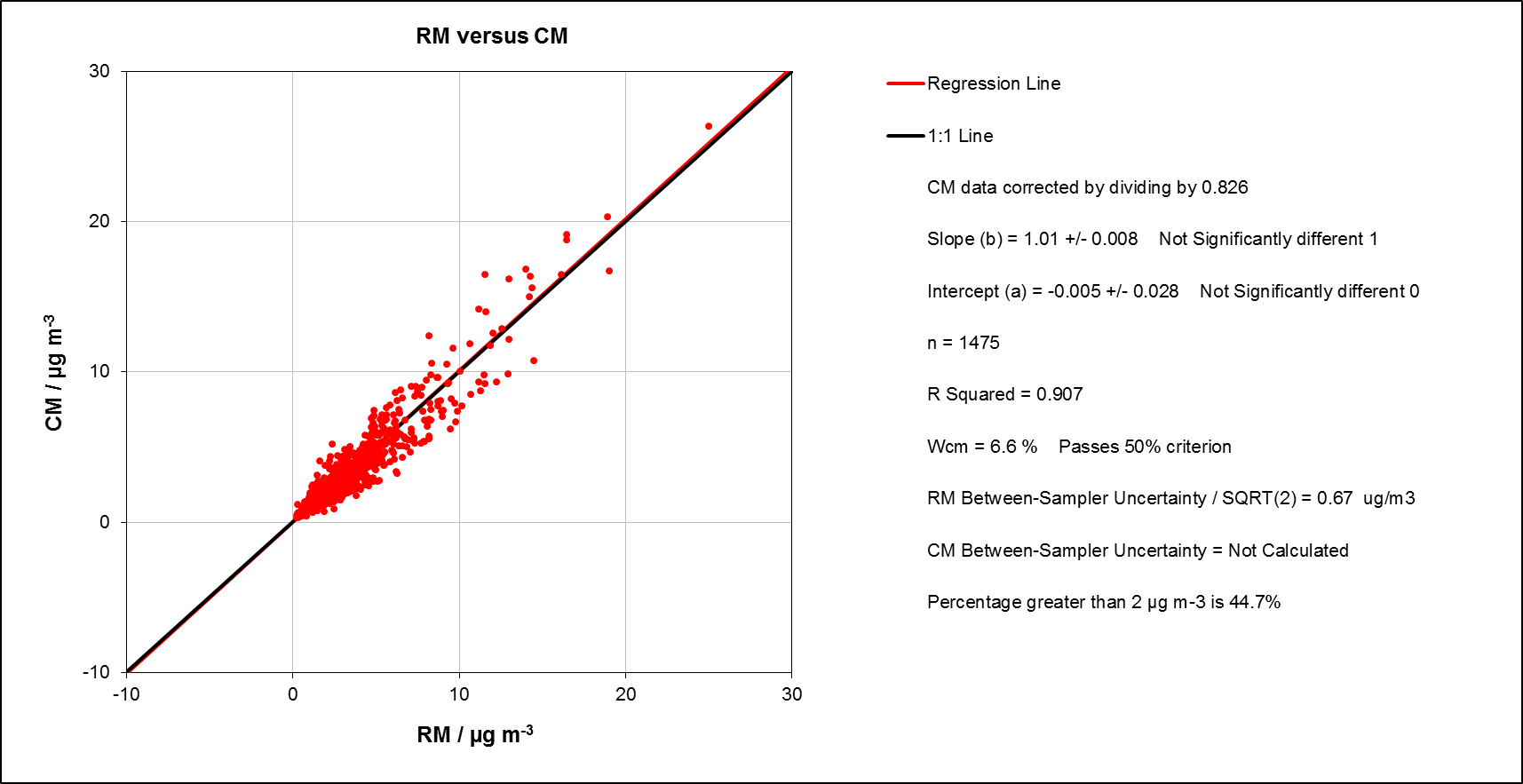


Figure S20, SO_4_^2-^, MARGA (PM_10_) vs RM (PM_10_), Melpitz, Germany.


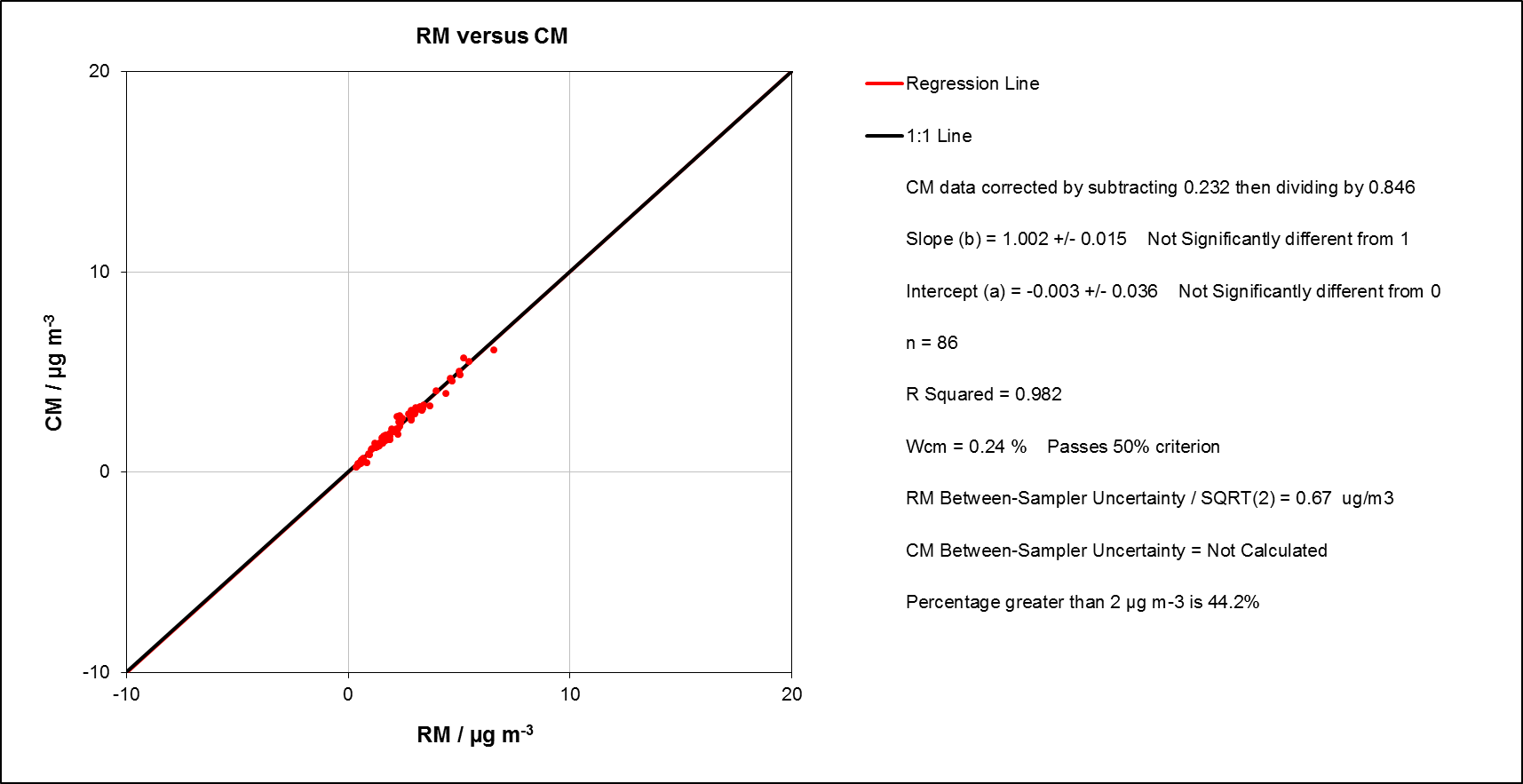


Figure S21, SO_4_^2-^, MARGA (PM_10_) vs RM (PM_10_), Kumpula, Finland.


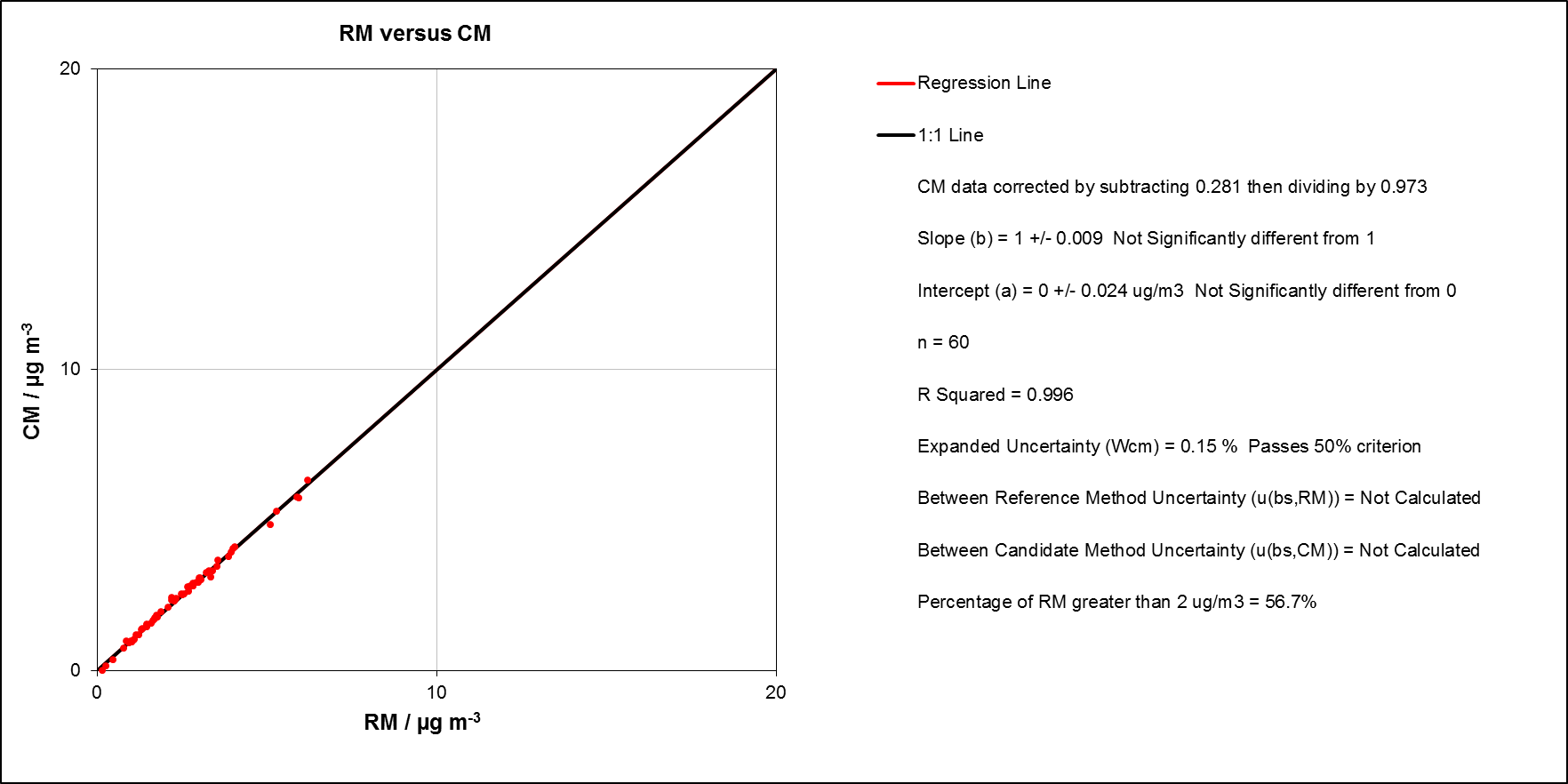


Figure S22a), SO_4_^2-^, MARGA CM1 (~PM_26_) vs RM (PM_2.5_), Research Triangle Park, United States.


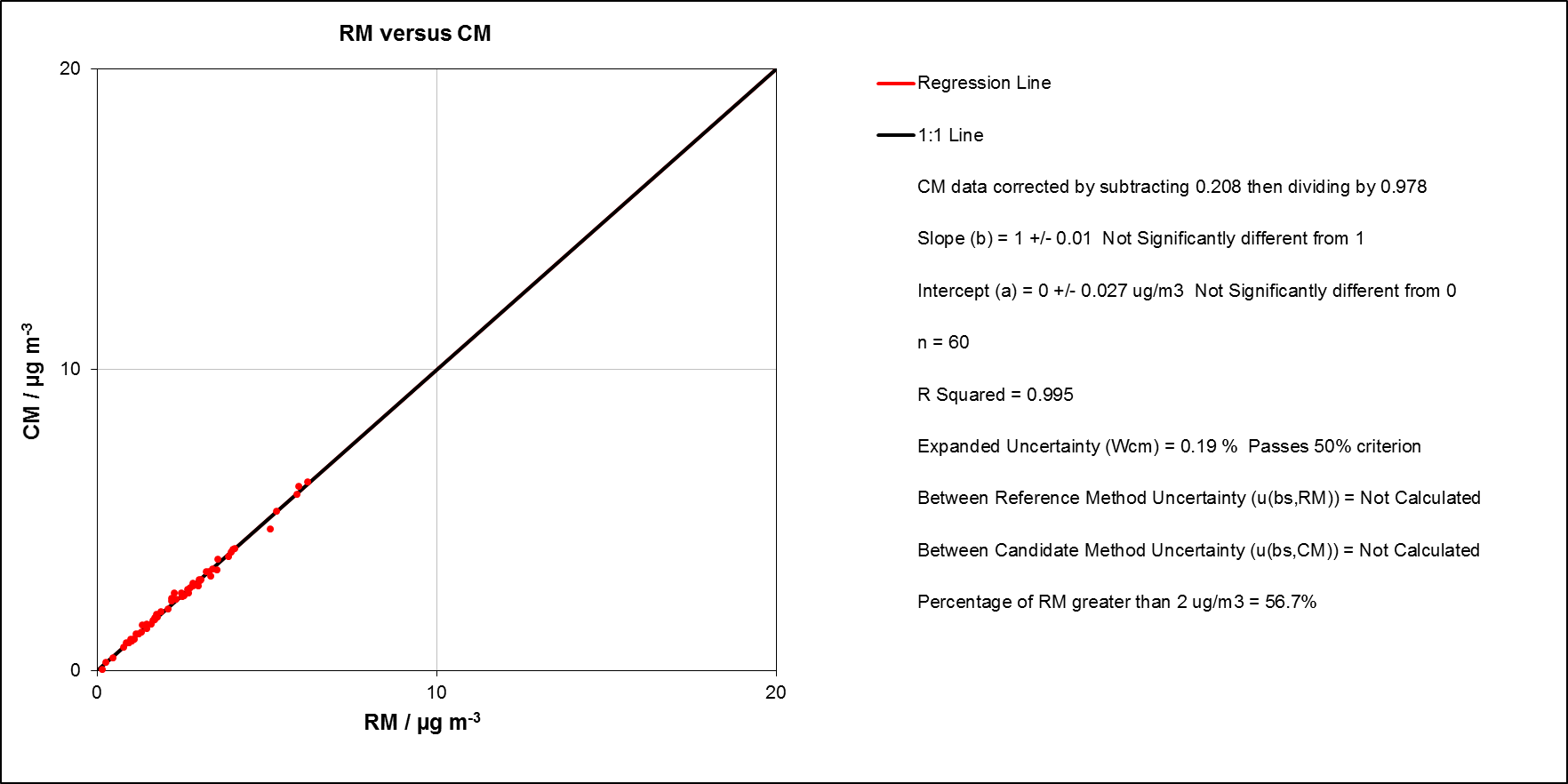


Figure S22b), SO_4_^2-^, MARGA CM2 (~PM_26_) vs RM (PM_2.5_), Research Triangle Park, United States.


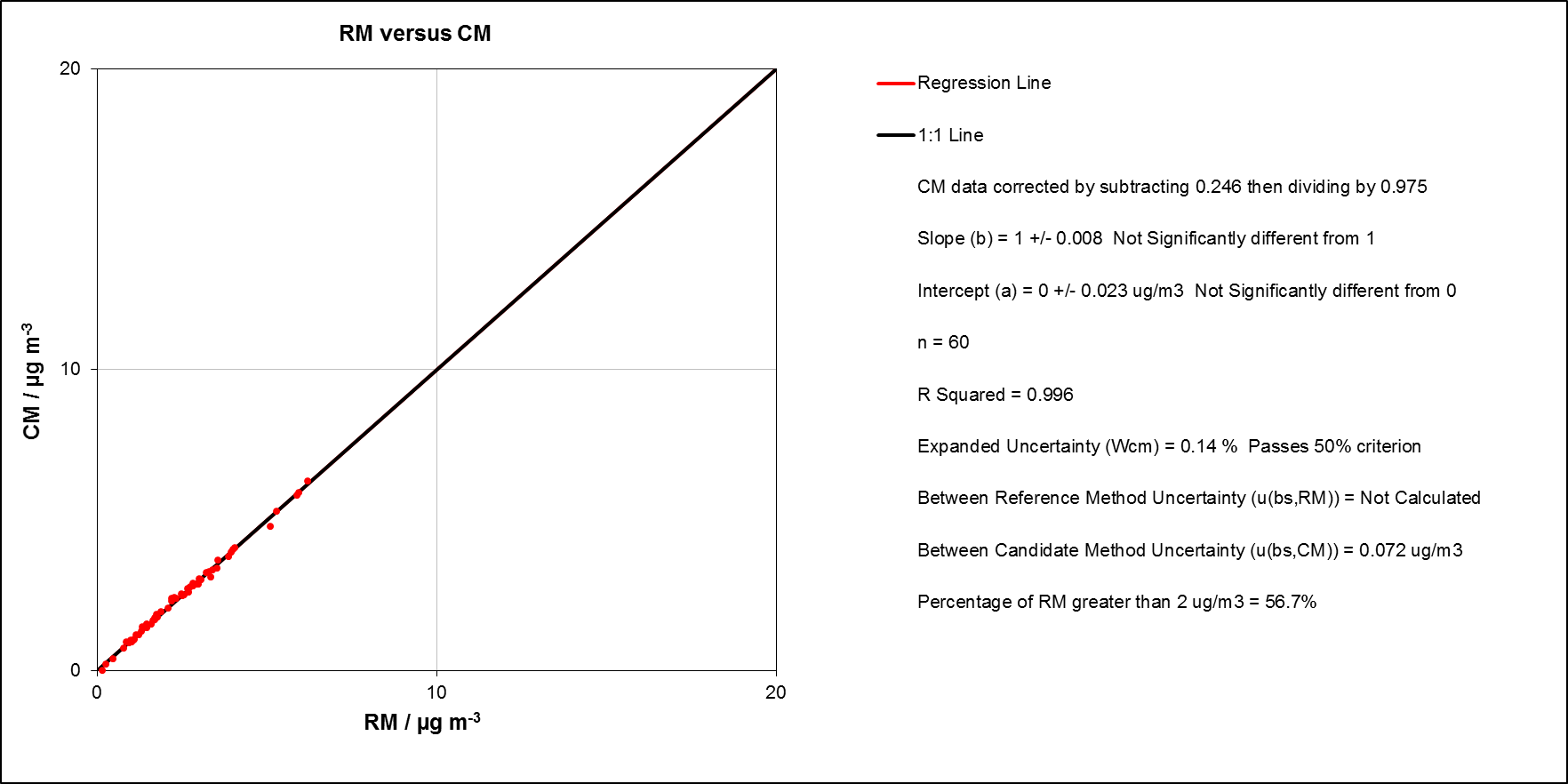


Figure S22c), SO_4_^2-^, averaged MARGA (~PM_26_) vs RM (PM_2.5_), Research Triangle Park, United States.


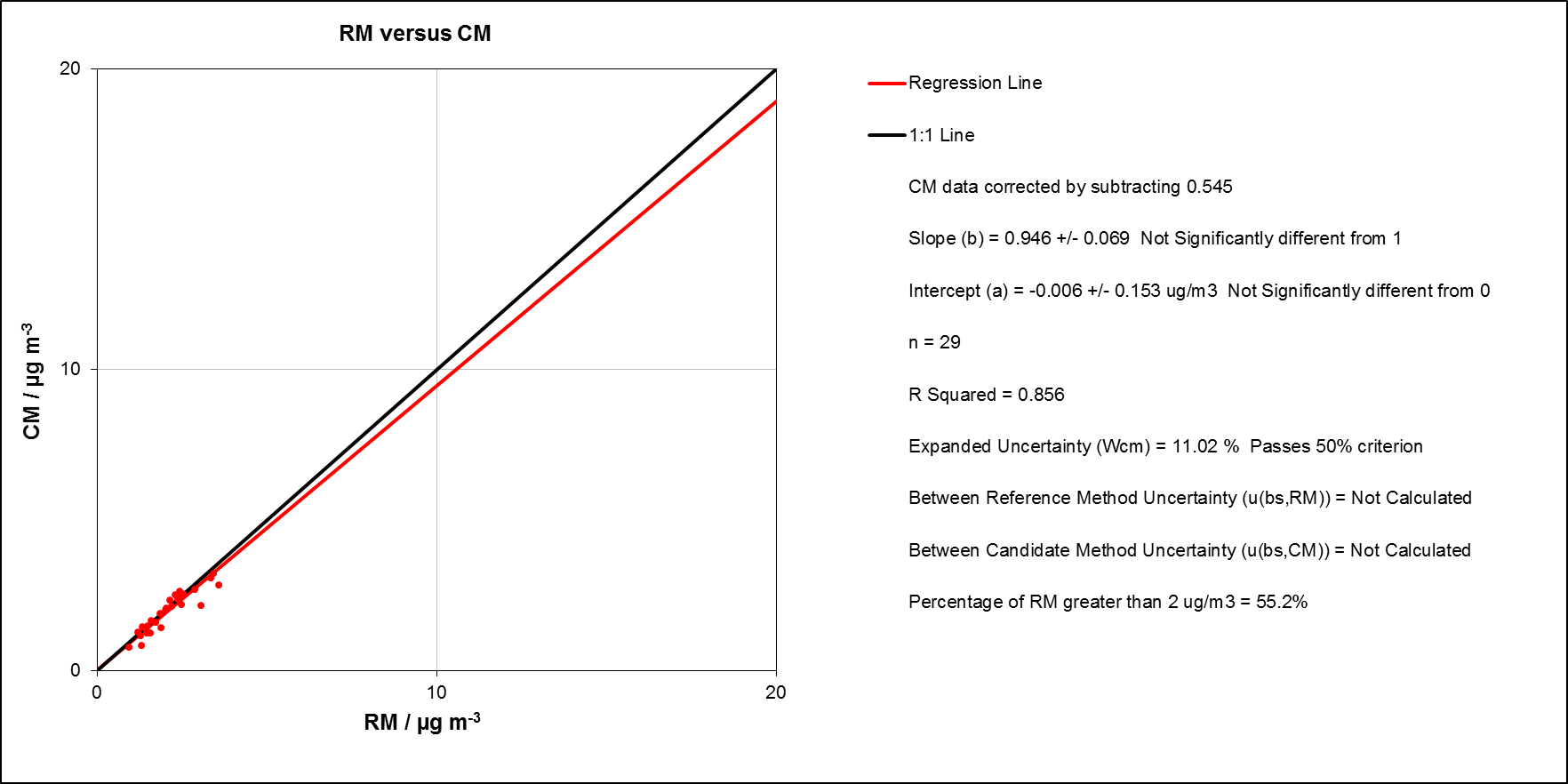


Figure S23, SO_4_^2-^, MARGA (PM_1_) vs RM (PM_1_), San Pietro Capofiume, Italy


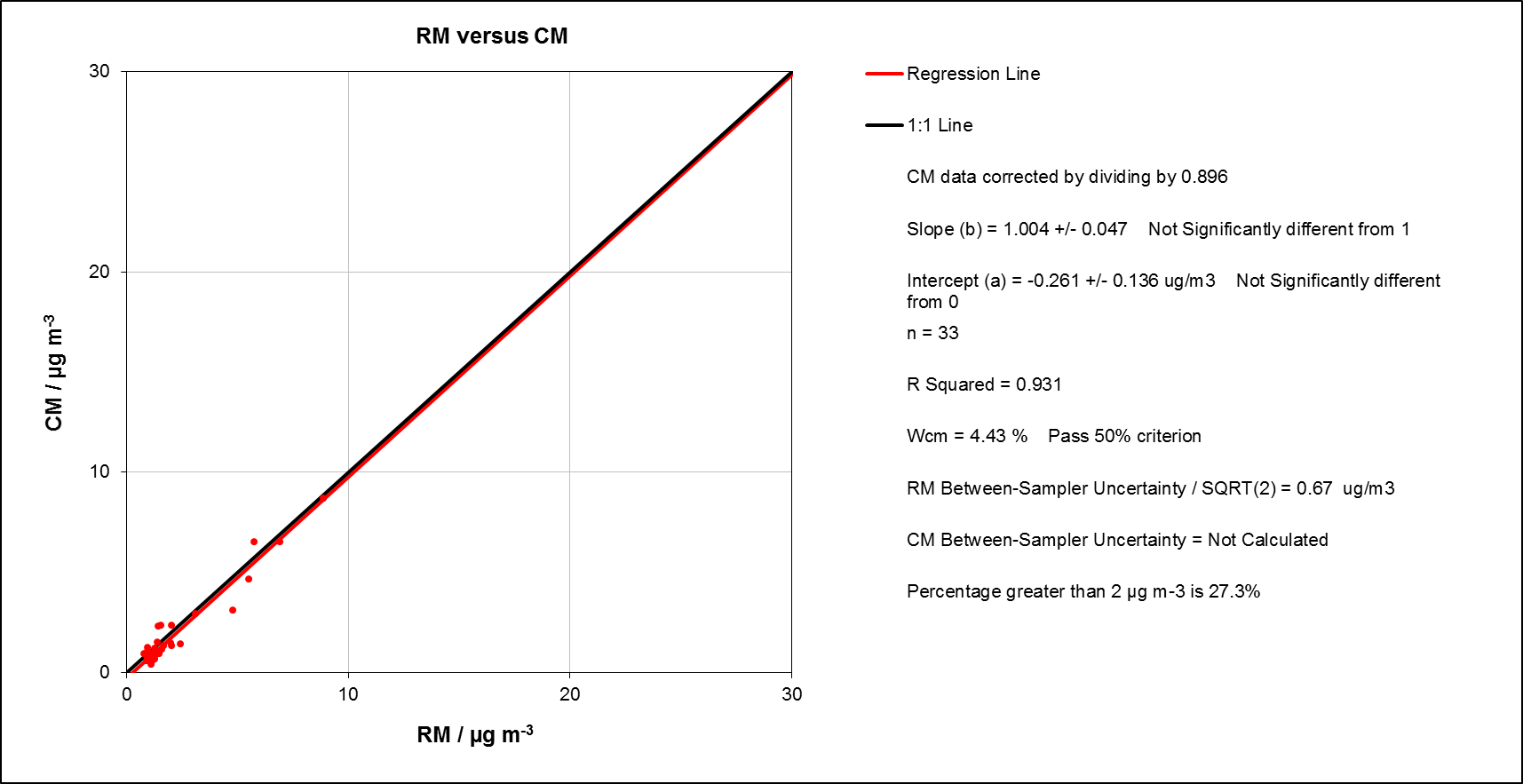


Figure S24, SO_4_^2-^, AIM (PM_10_) vs RM (PM_10_), North Kensington, UK.

1. Na^+^


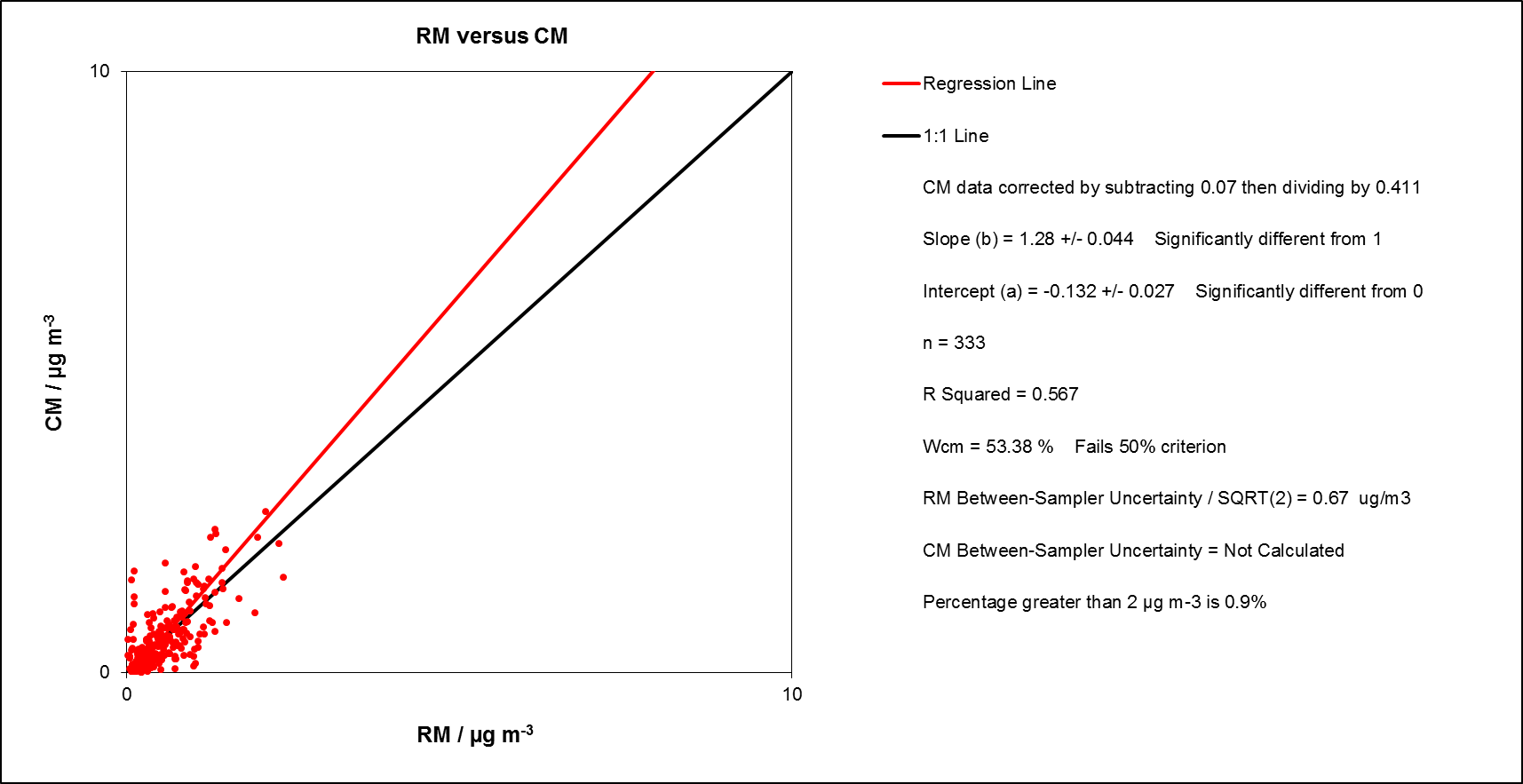


Figure S25, Na^+^, MARGA (PM_10_) vs RM (PM_10_), Melpitz, Germany.


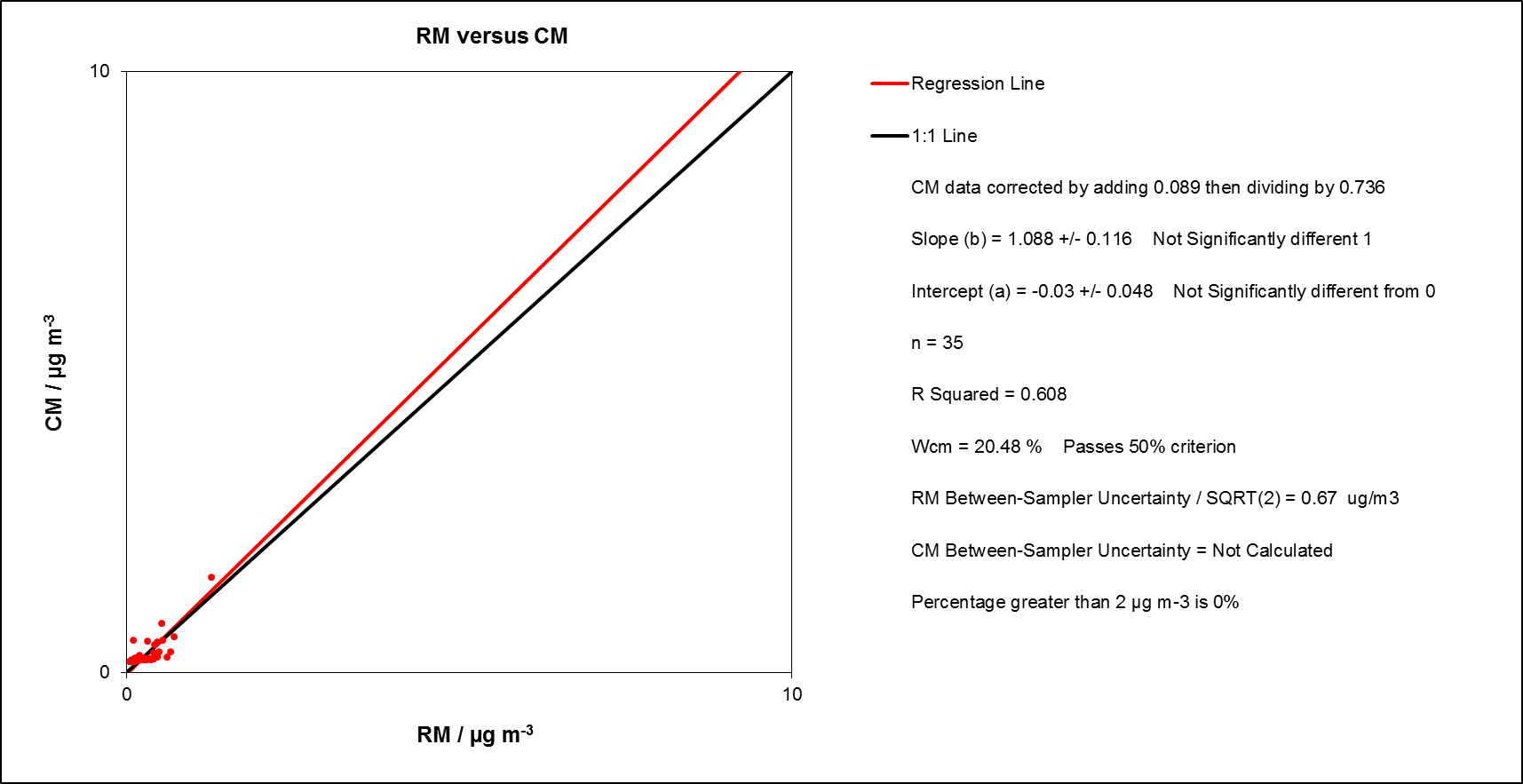


Figure S26, Na^+^, MARGA (PM_10_) vs RM (PM_10_), Kumpula, Finland.


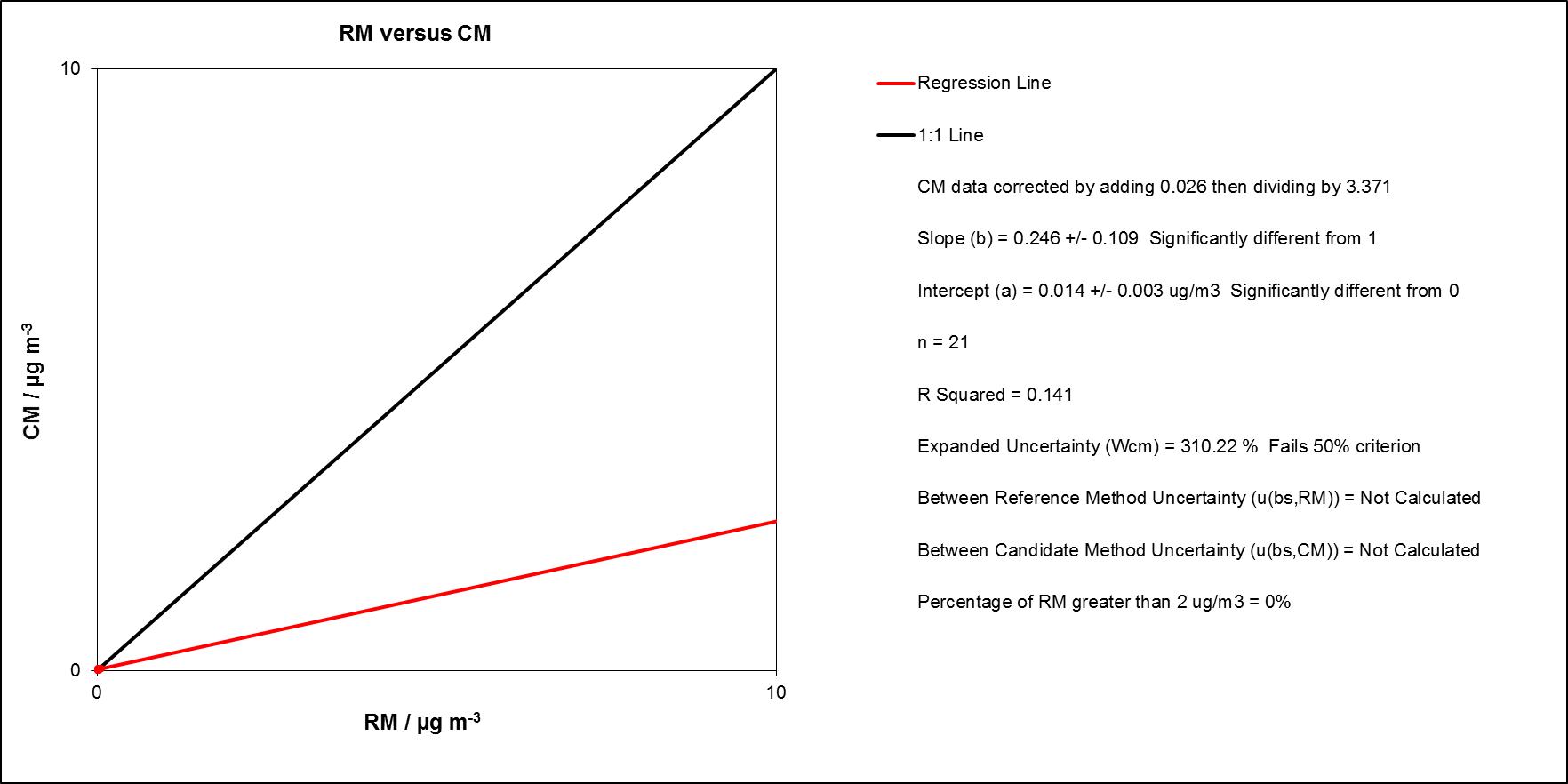


Figure S27, Na^+^, MARGA (PM_1_) vs RM (PM_1_), San Pietro Capofiume, Italy


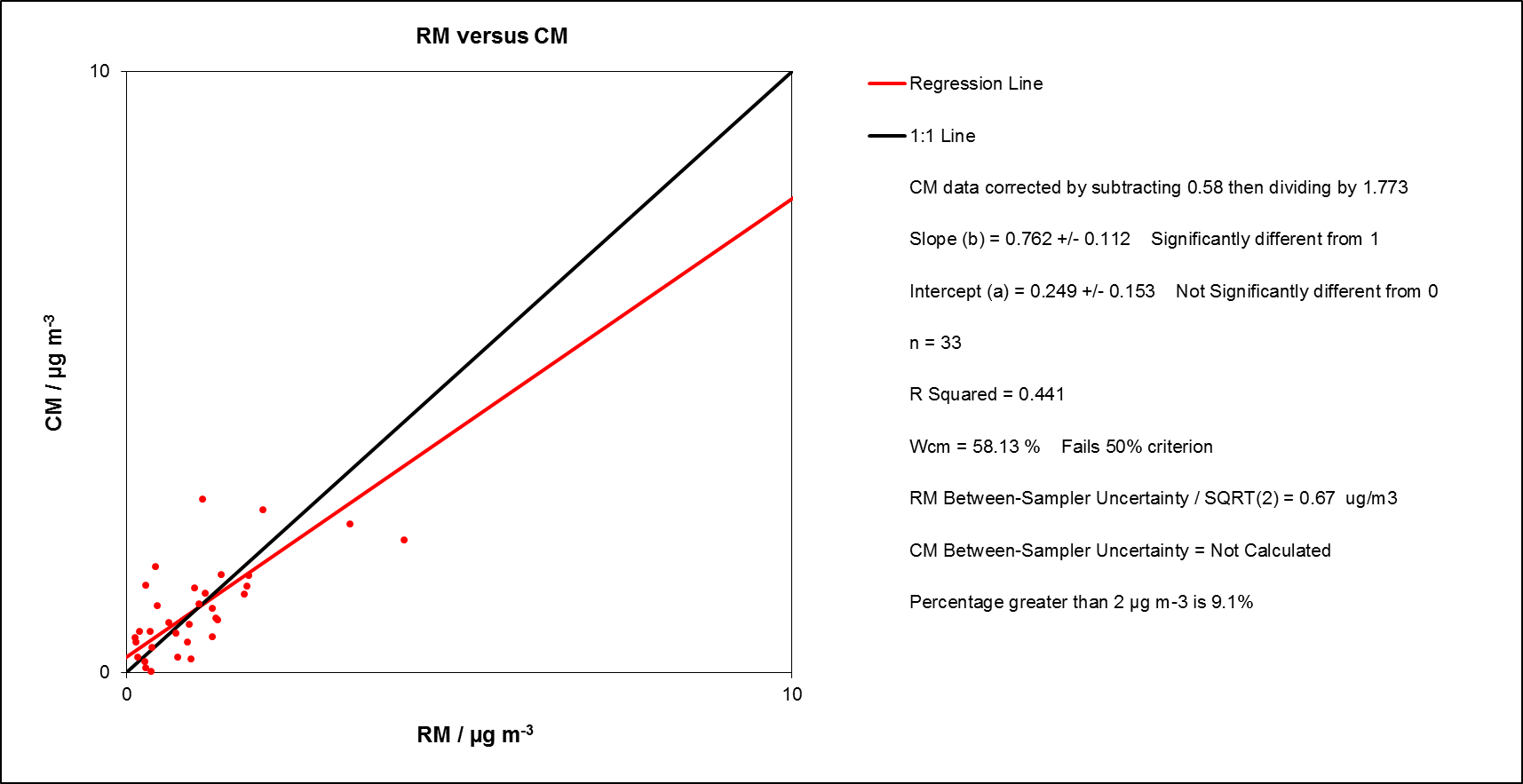


Figure S28, Na^+^, AIM (PM_10_) vs RM (PM_10_), North Kensington, UK.

1. Cl^-^


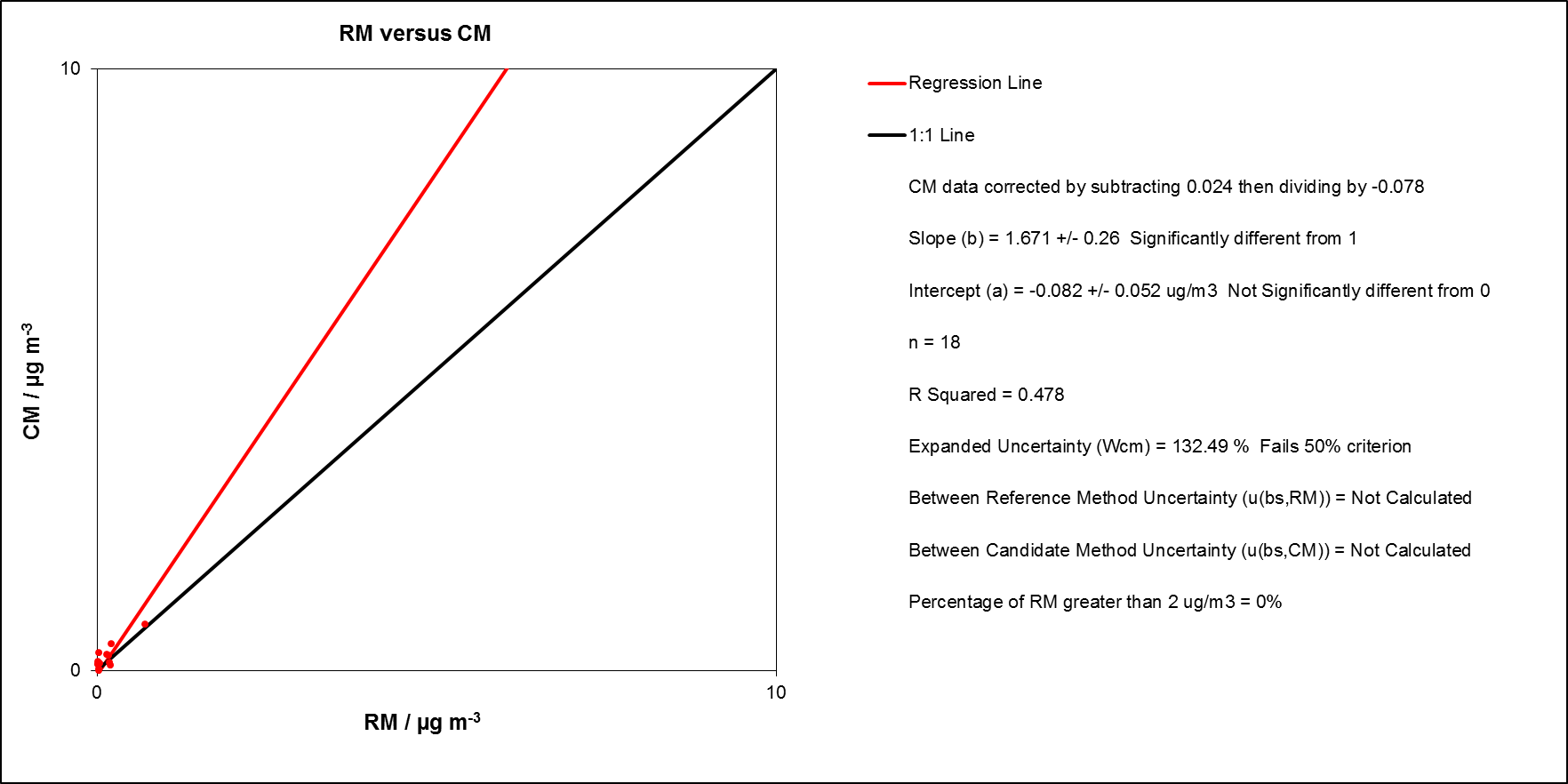


Figure S29, Cl^-^, ACSM (PM_1_) vs RM (PM_2.5_), Revin, France


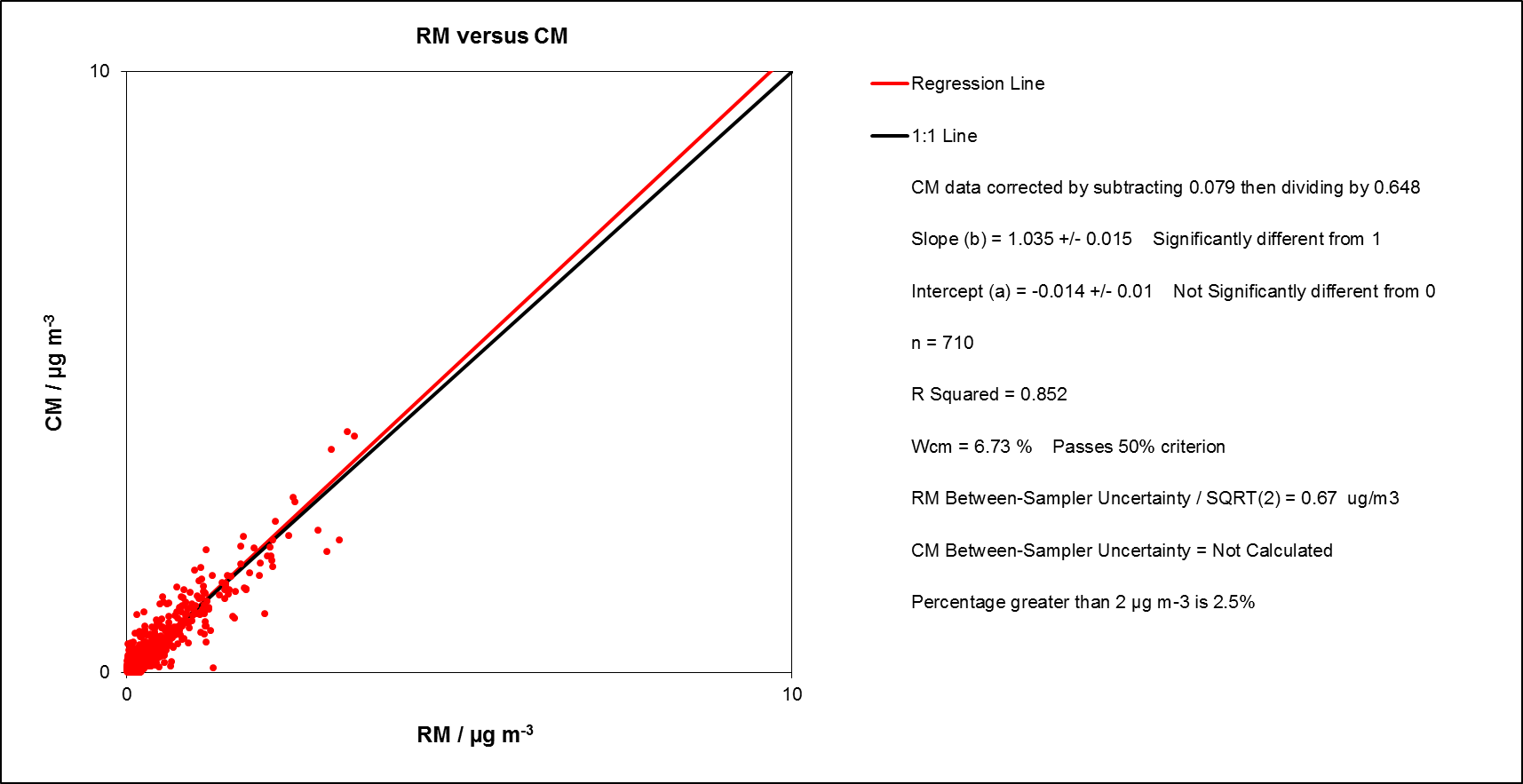


Figure S30, Cl^-^, MARGA (PM_10_) vs RM (PM_10_), Melpitz, Germany.


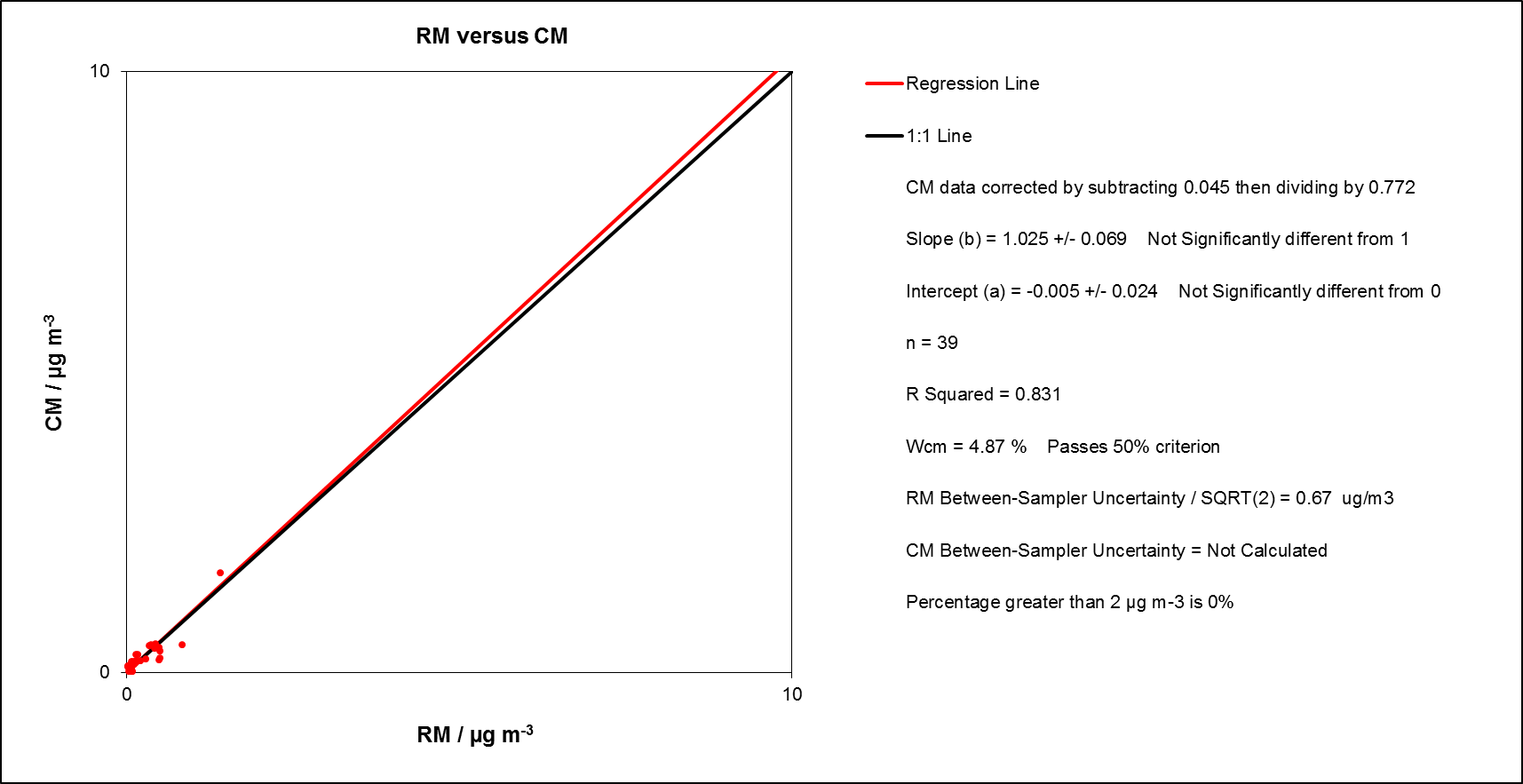


Figure S31, Cl^-^, MARGA (PM_10_) vs RM (PM_10_), Kumpula, Finland.


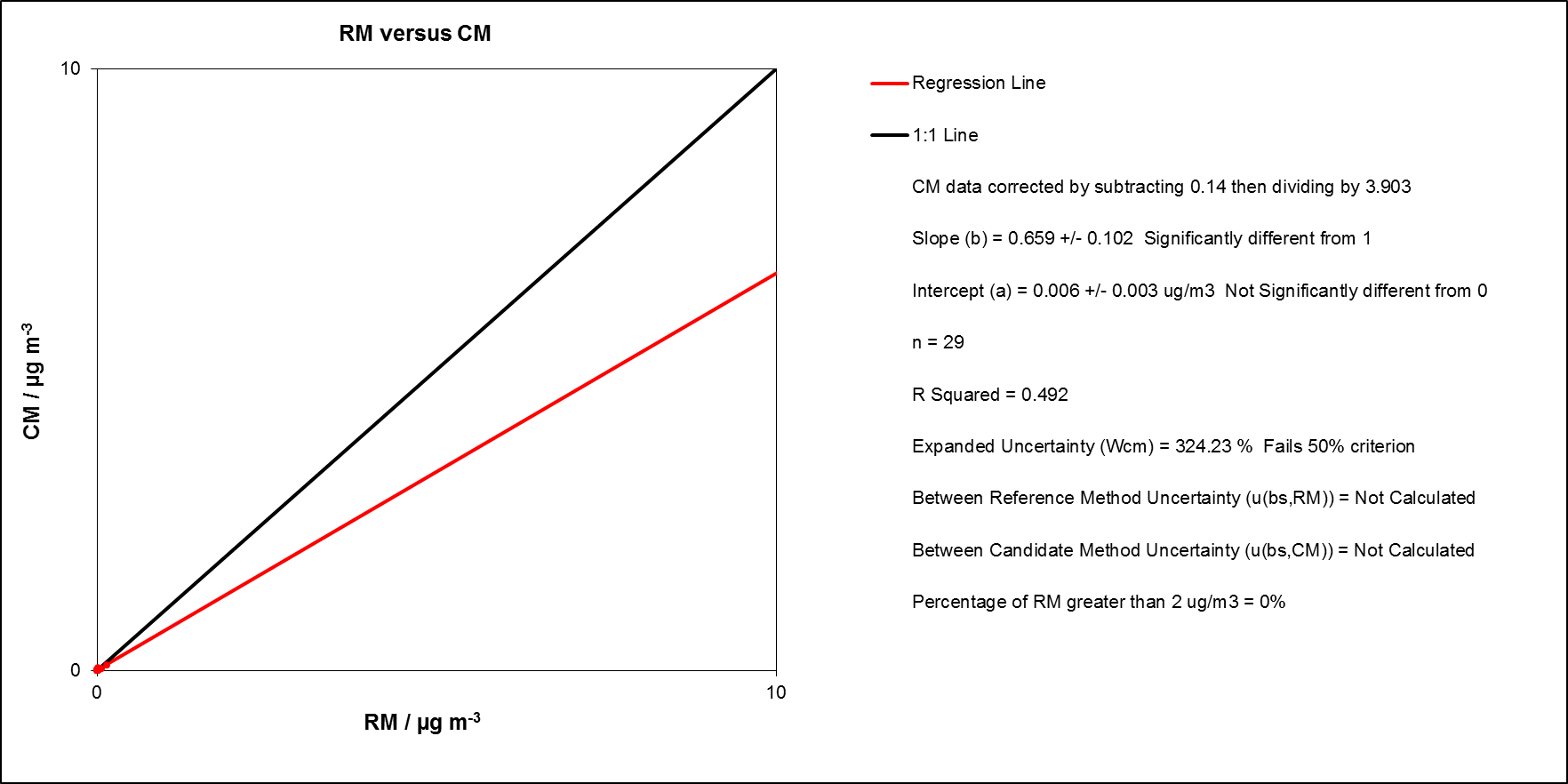


Figure S32, Cl^-^, MARGA (PM_1_) vs RM (PM_1_), San Pietro Capofiume, Italy


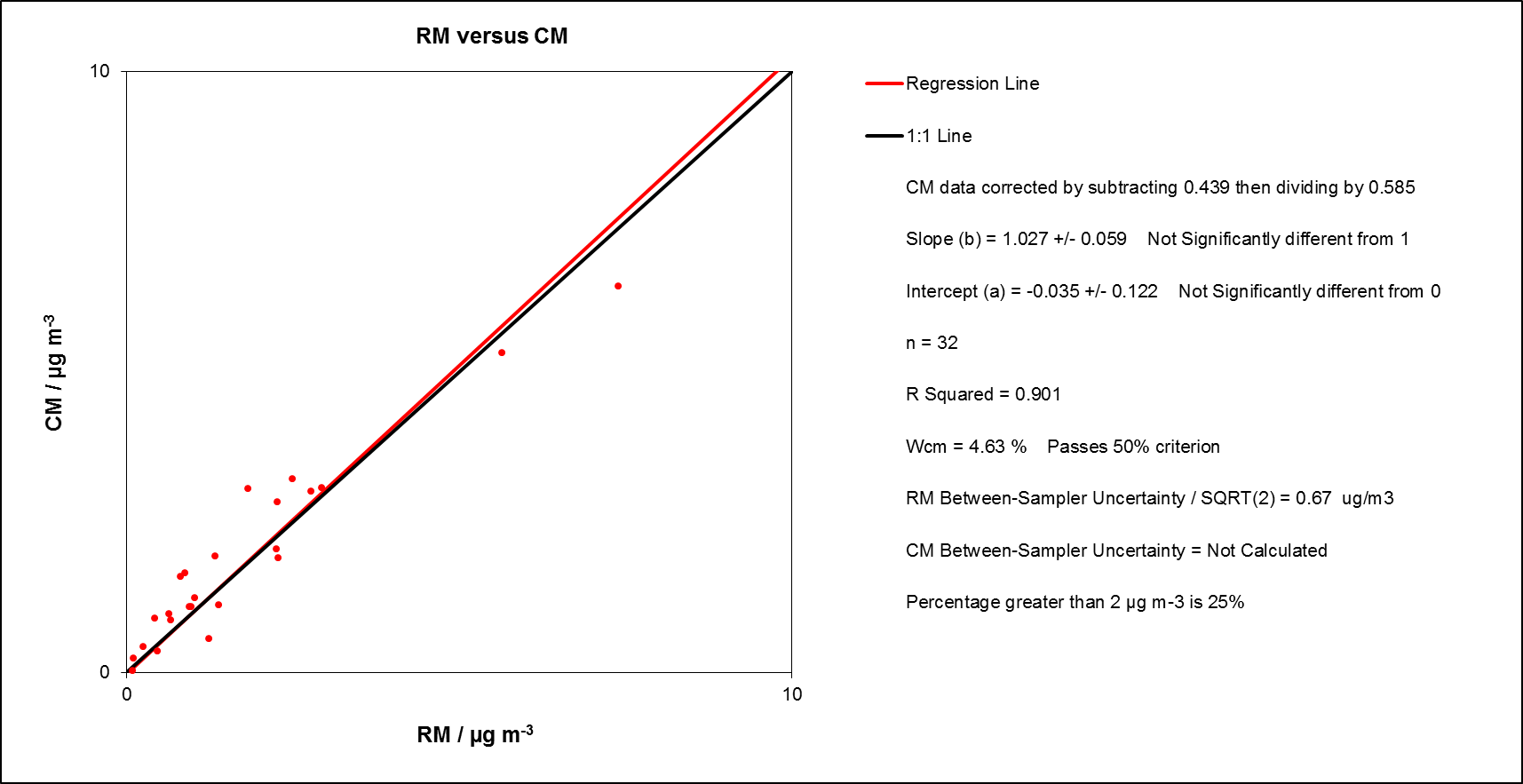


Figure S33, Cl^-^, AIM (PM_10_) vs RM (PM_10_), North Kensington, UK.

1. K^+^


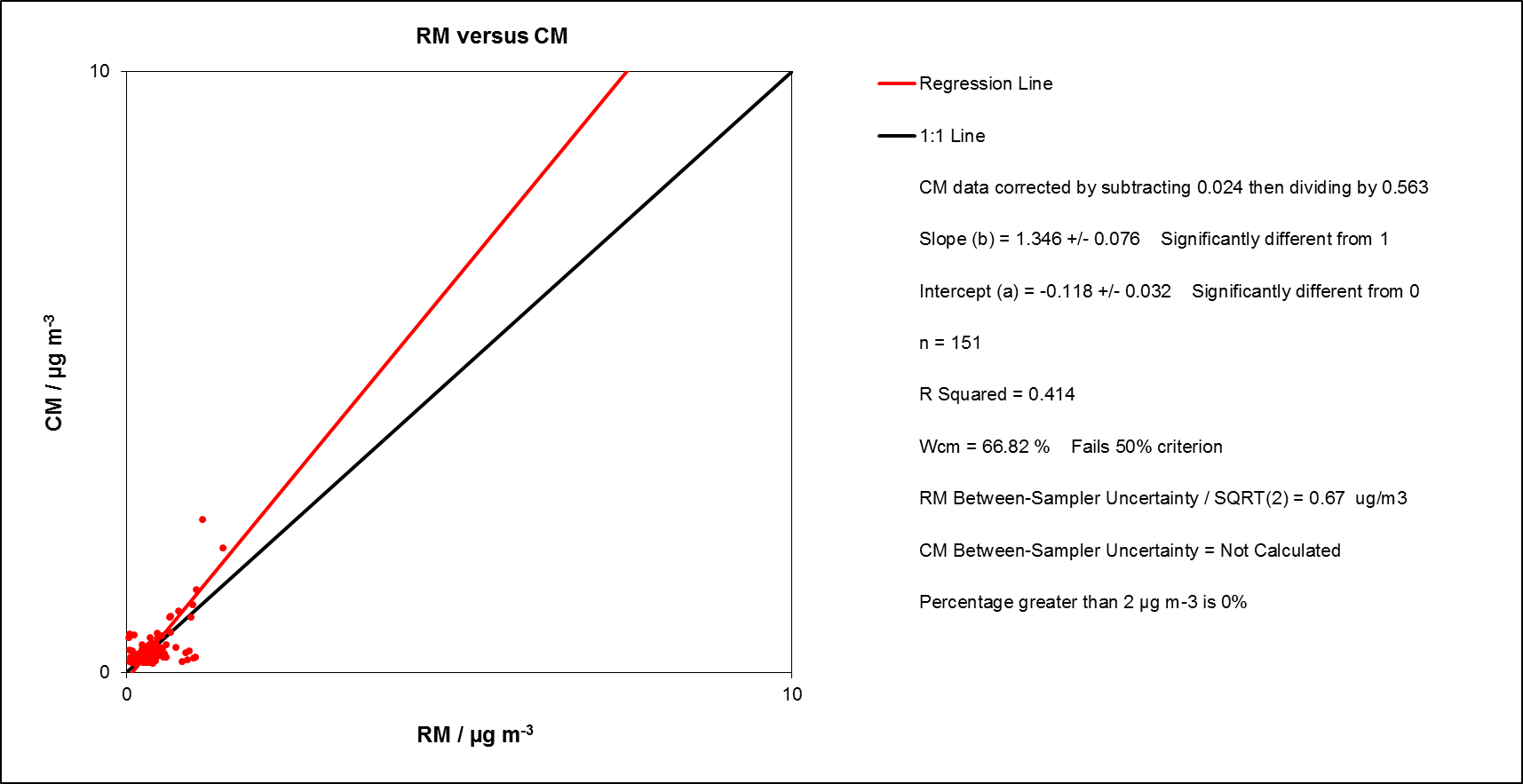


Figure S34, K^+^, MARGA (PM_10_) vs RM (PM_10_), Melpitz, Germany.


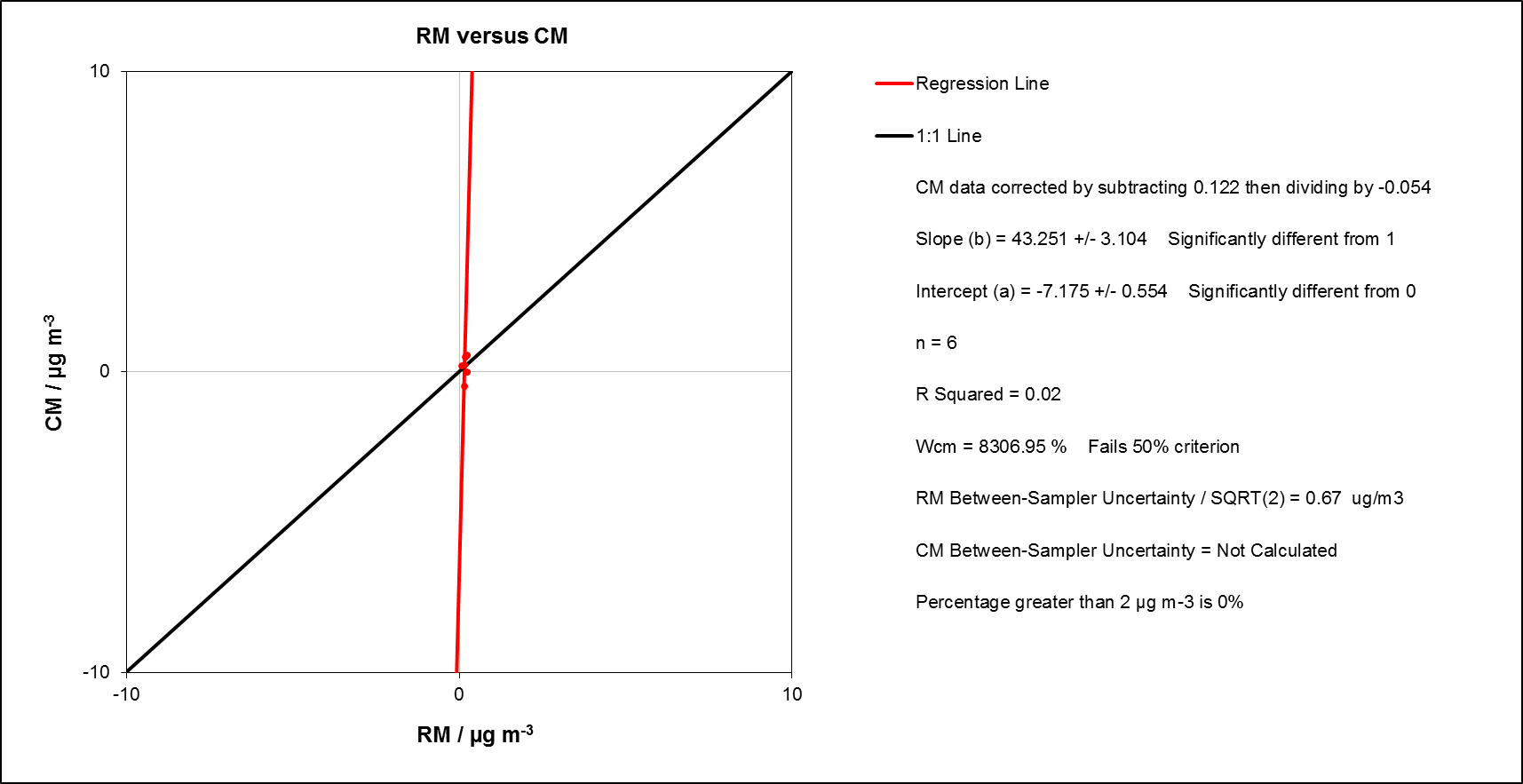


Figure S35, K^+^, MARGA (PM_10_) vs RM (PM_10_), Kumpula, Finland.


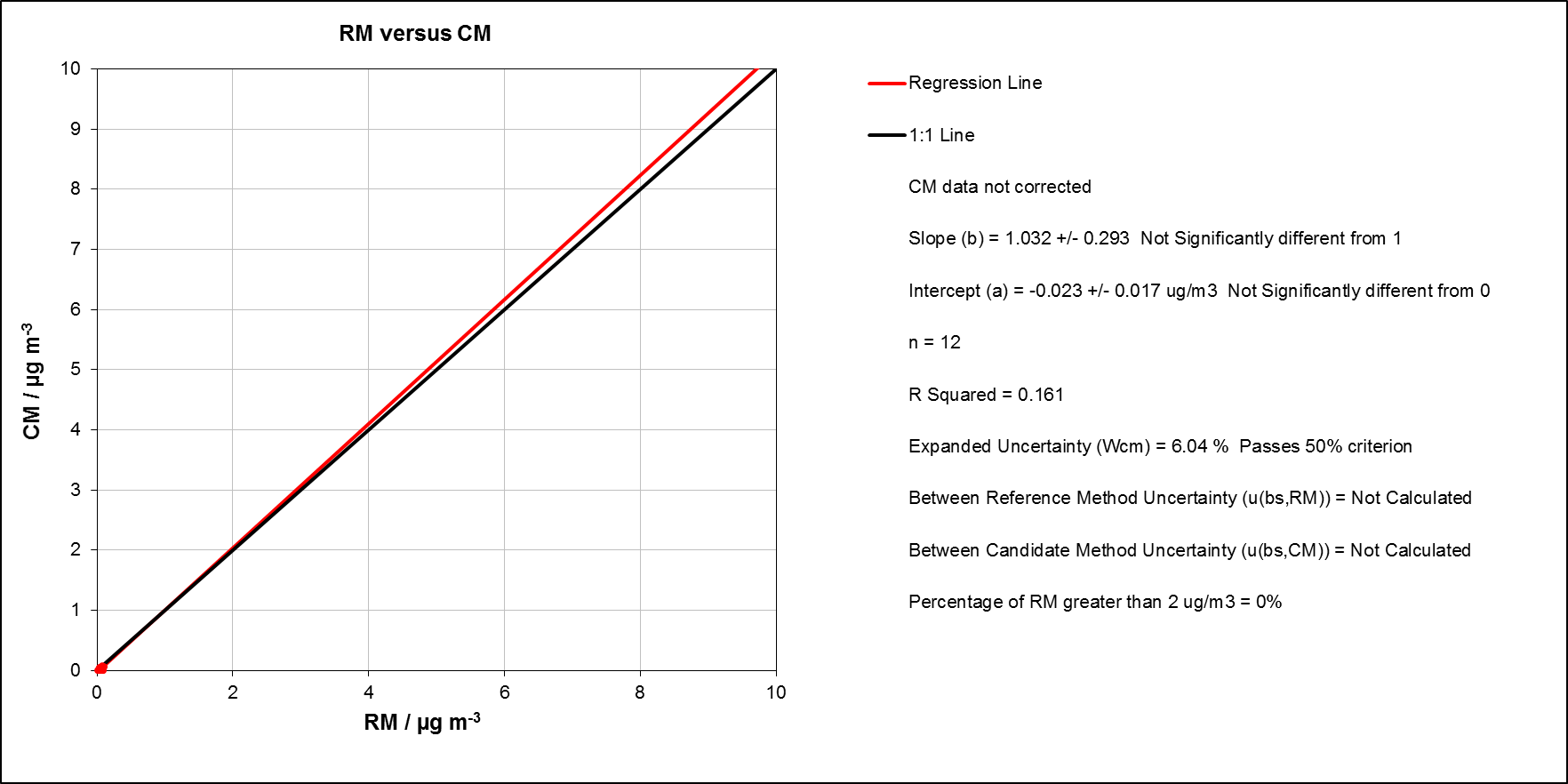


Figure S36, K^+^, MARGA (PM_1_) vs RM (PM_1_), San Pietro Capofiume, Italy


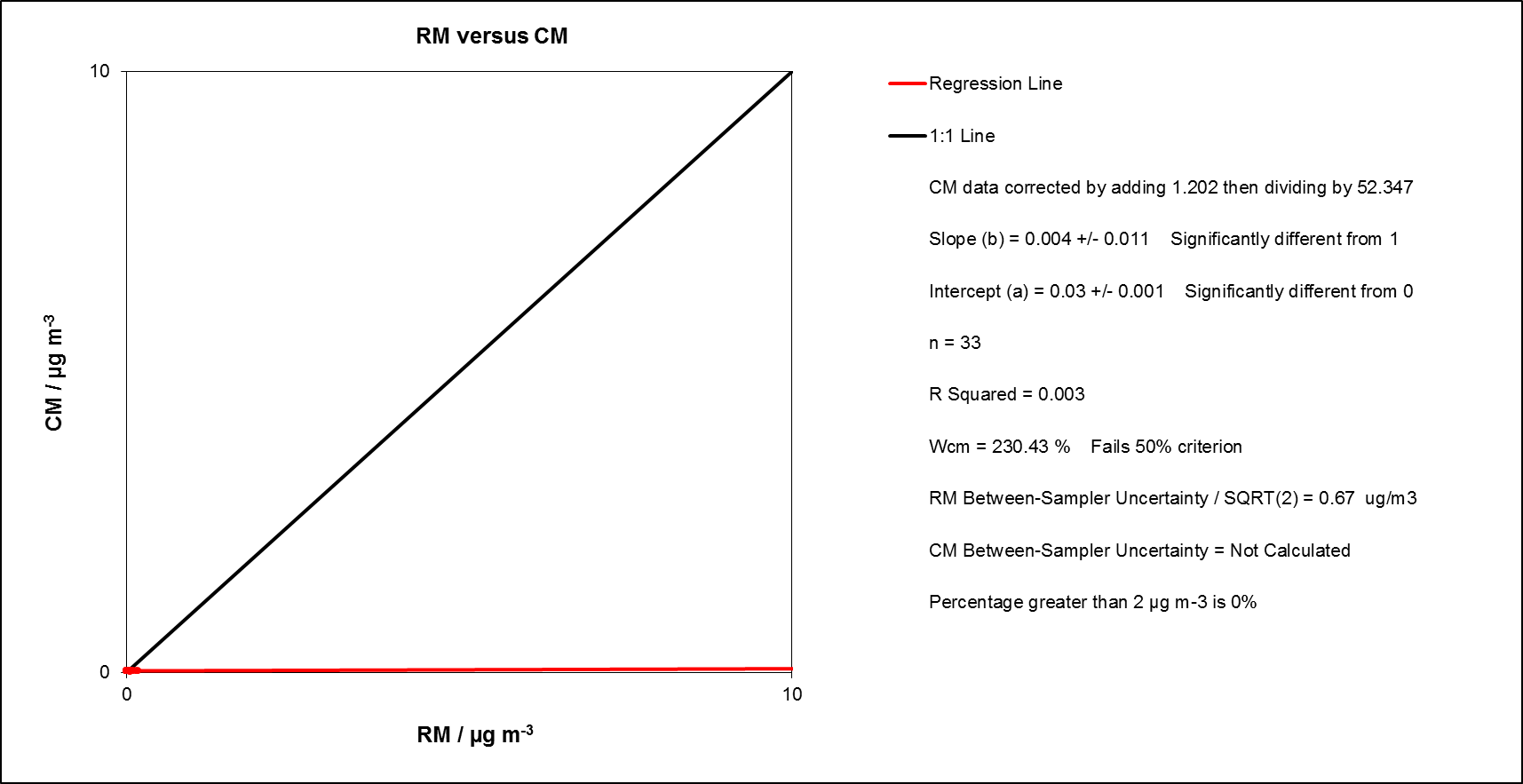


Figure S37, K^+^, AIM (PM_10_) vs RM (PM_10_), North Kensington, UK.

1. Ca^2+^


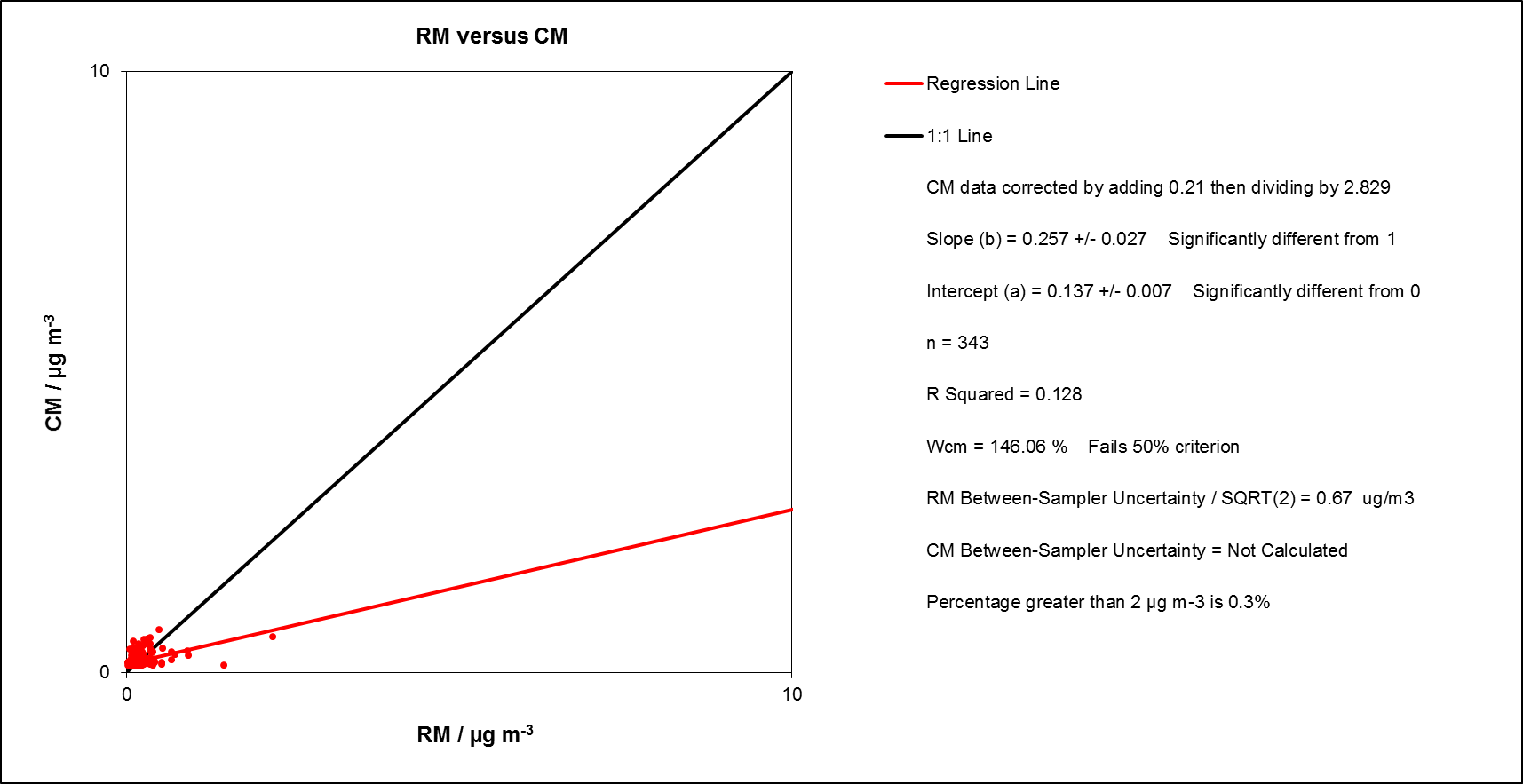


Figure S38, Ca^2+^, MARGA (PM_10_) vs RM (PM_10_), Melpitz, Germany.


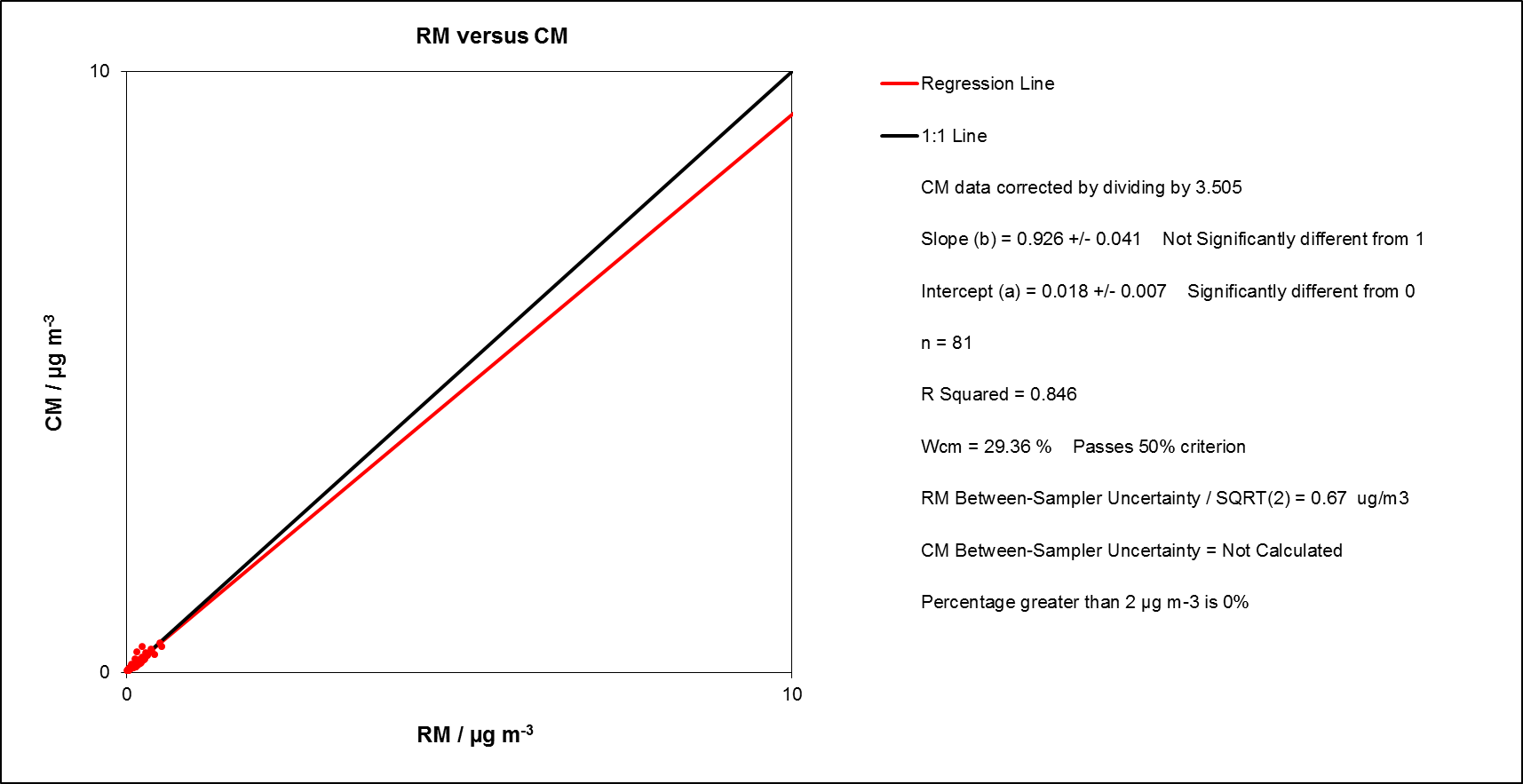


Figure S39, Ca^2+^, MARGA (PM_10_) vs RM (PM_10_), Kumpula, Finland.


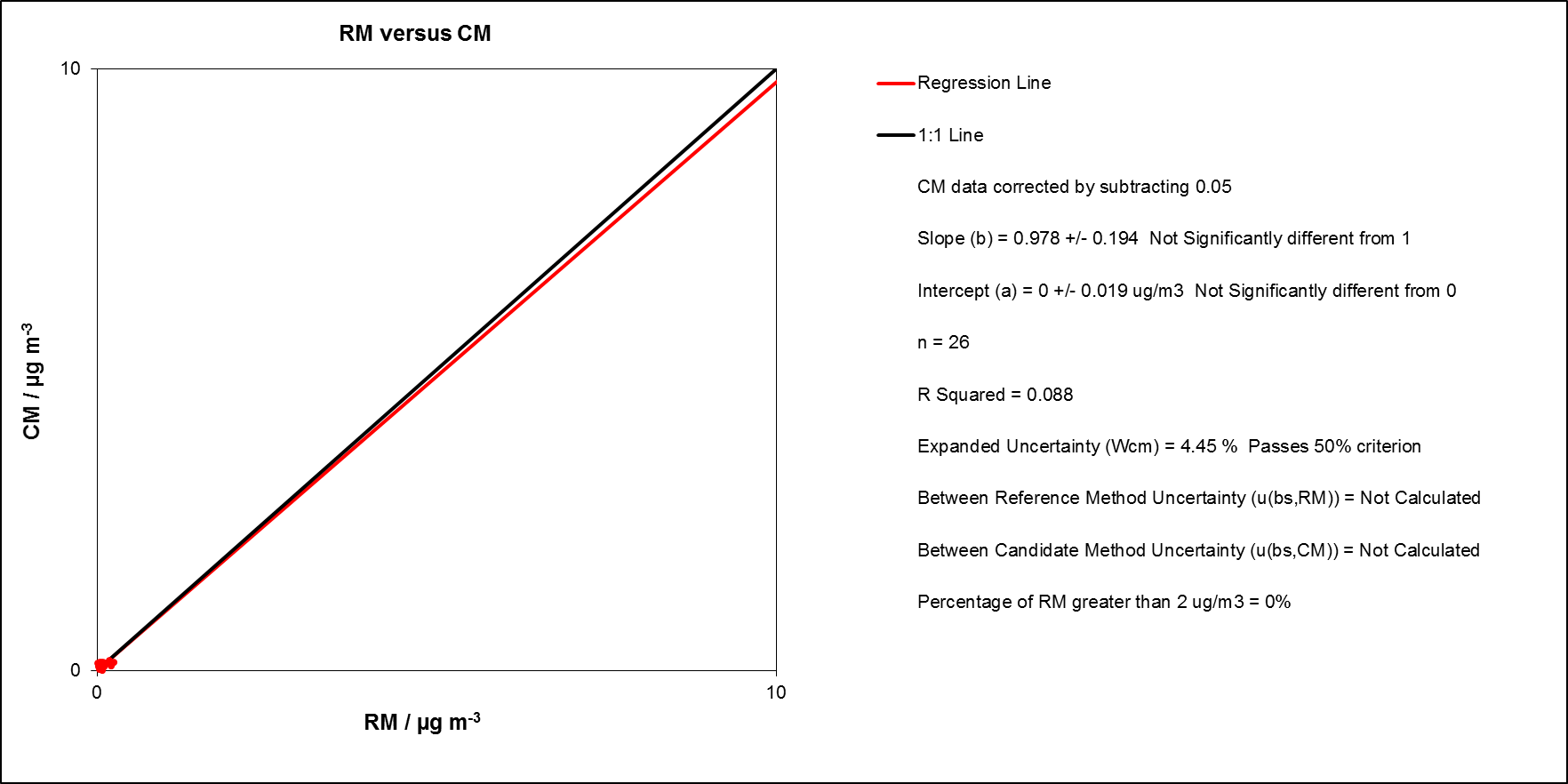


Figure S40, Ca^2+^, MARGA (PM_1_) vs RM (PM_1_), San Pietro Capofiume, Italy


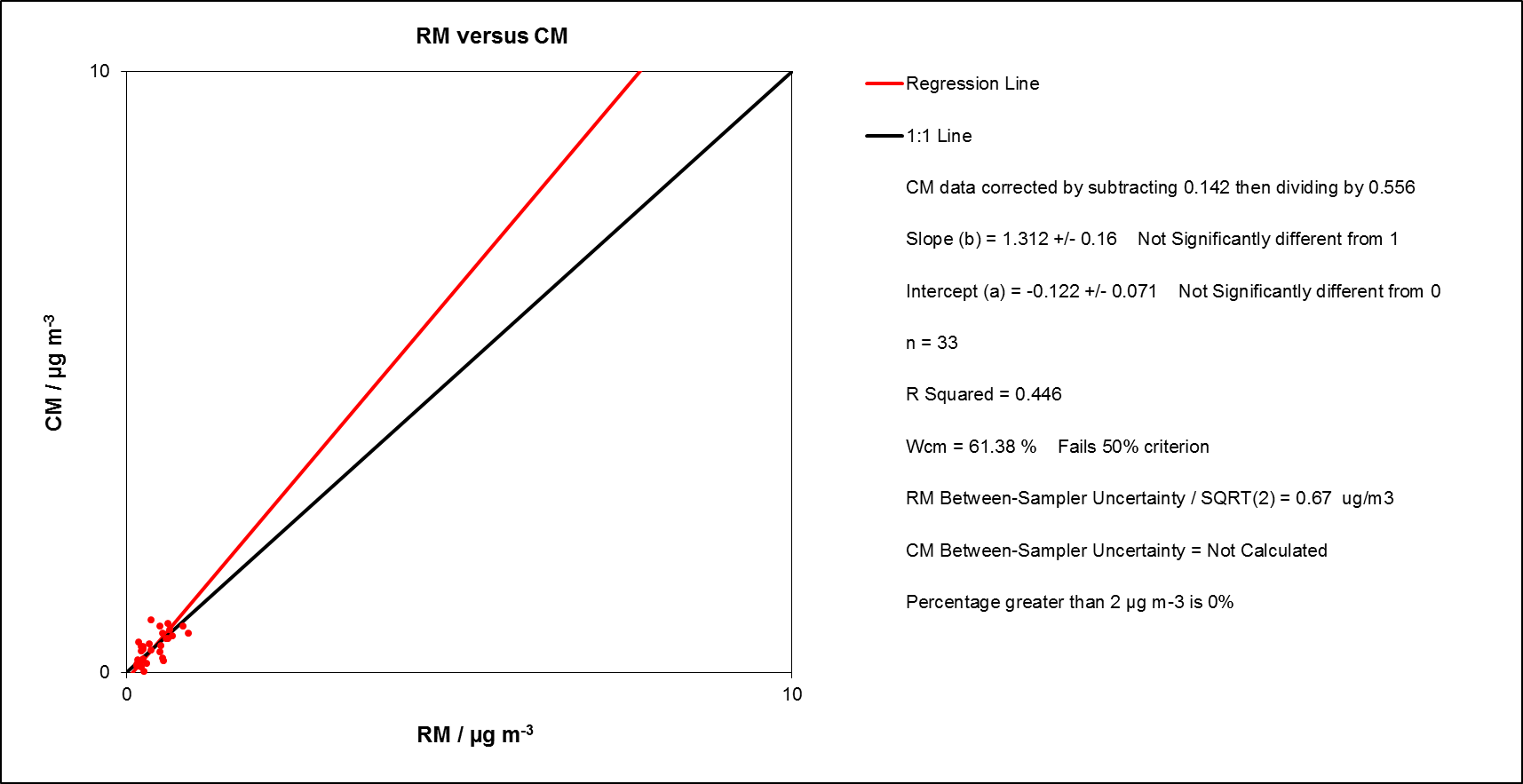


Figure S41, Ca^2+^, AIM (PM_10_) vs RM (PM_10_), North Kensington, UK.

1. Mg^2+^


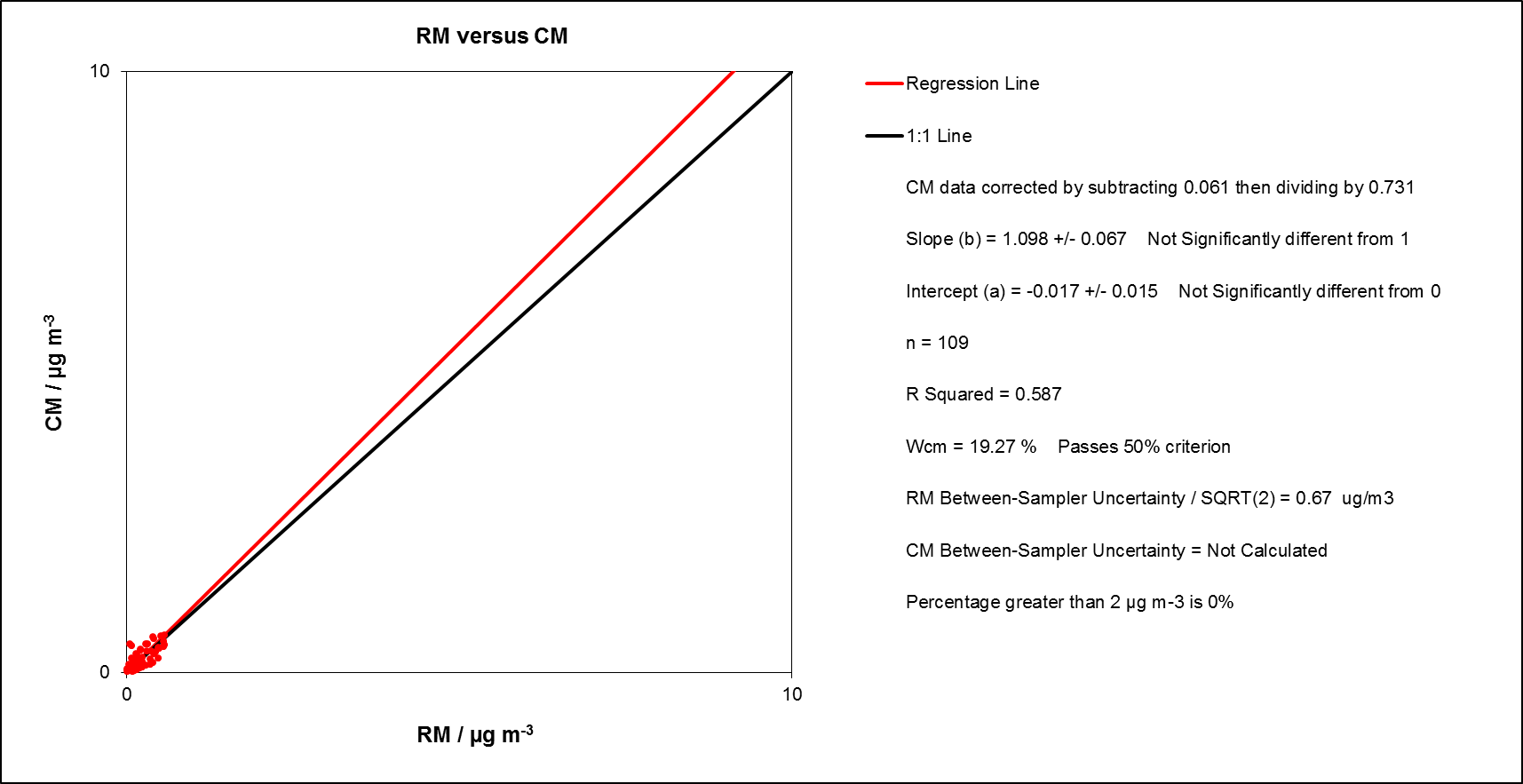


Figure S42, Mg^2+^, MARGA (PM_10_) vs RM (PM_10_), Melpitz, Germany.


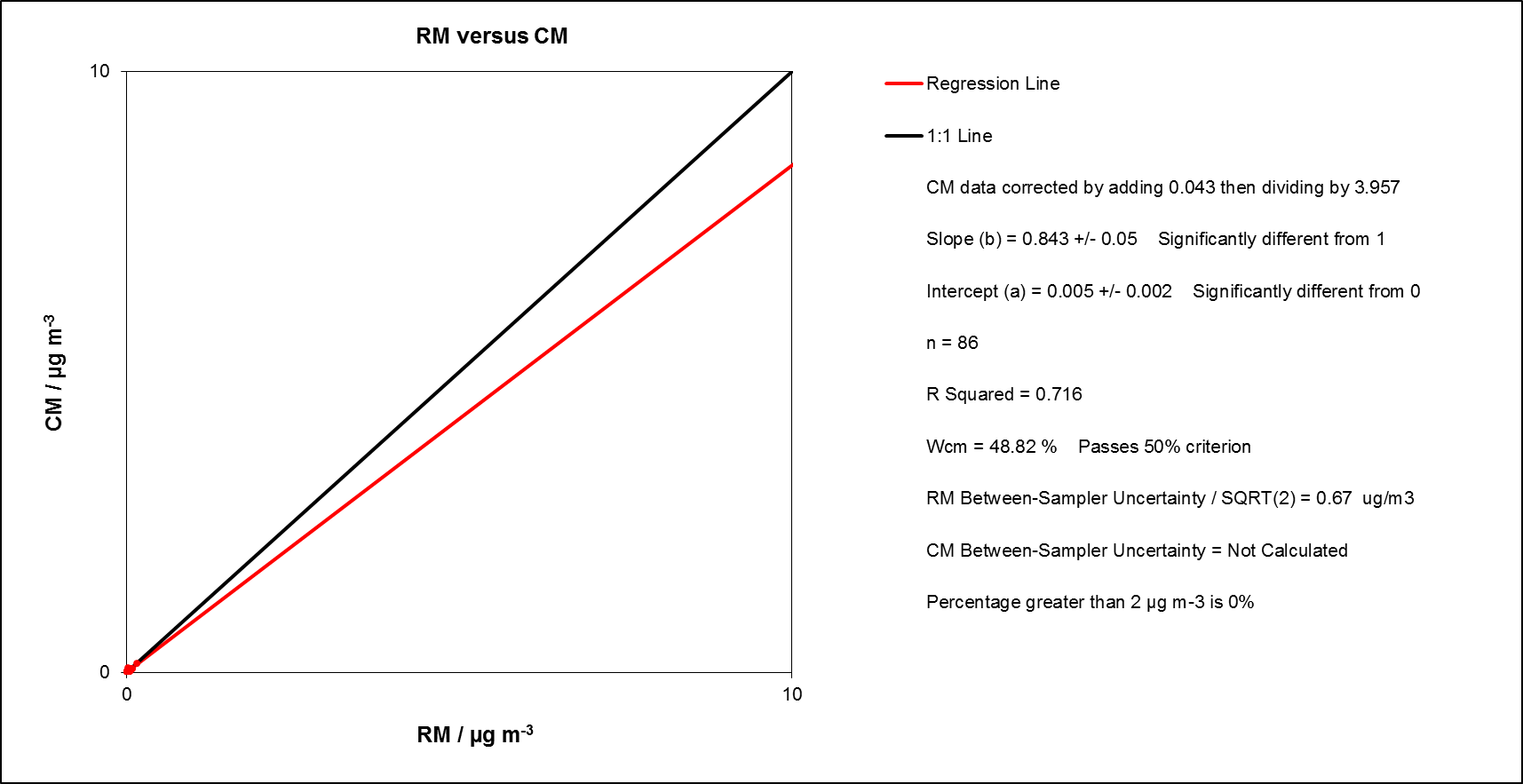


Figure S43, Mg^2+^, MARGA (PM_10_) vs RM (PM_10_), Kumpula, Finland.


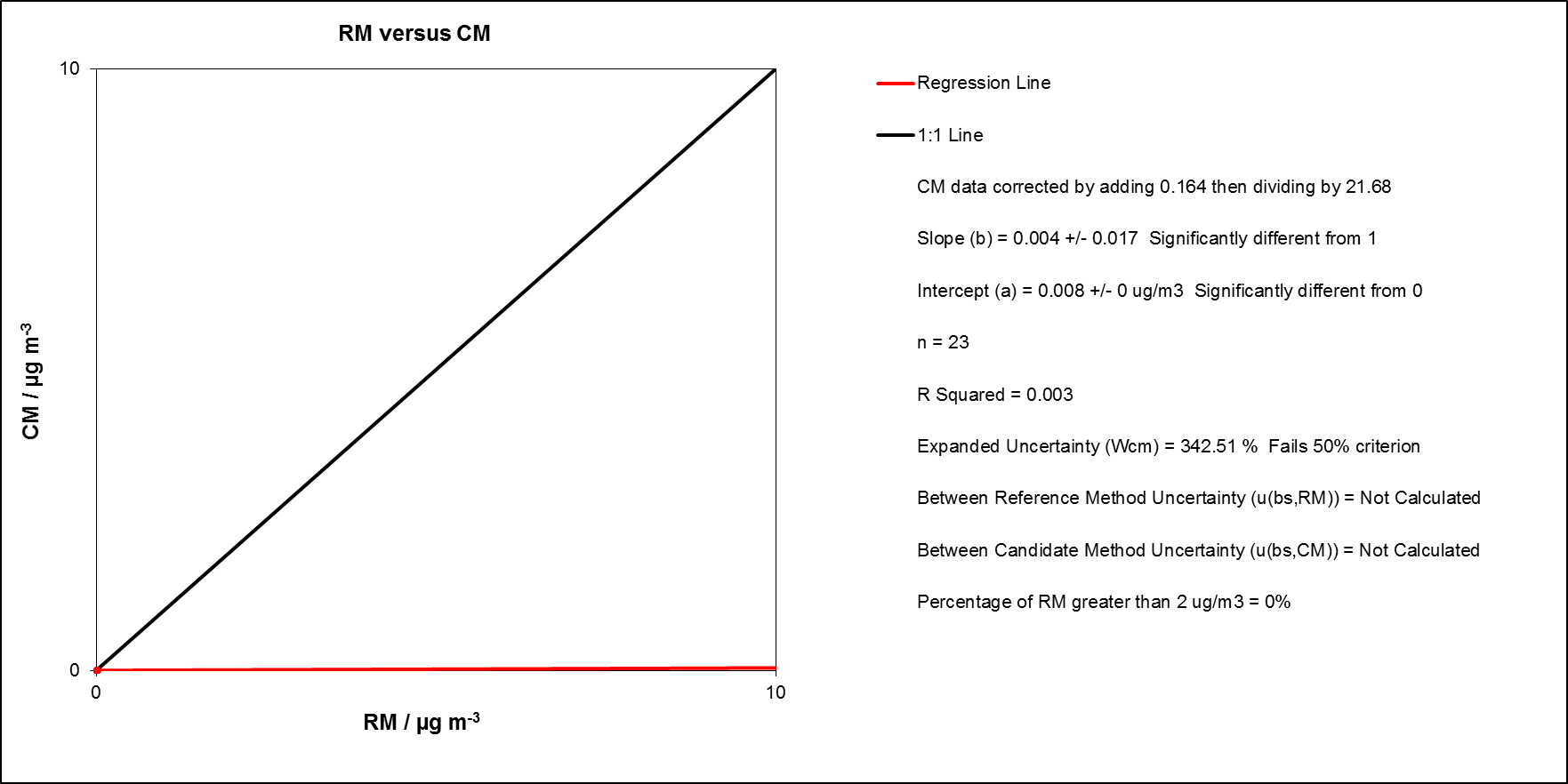


Figure S44, Mg^2+^, MARGA (PM_1_) vs RM (PM_1_), San Pietro Capofiume, Italy


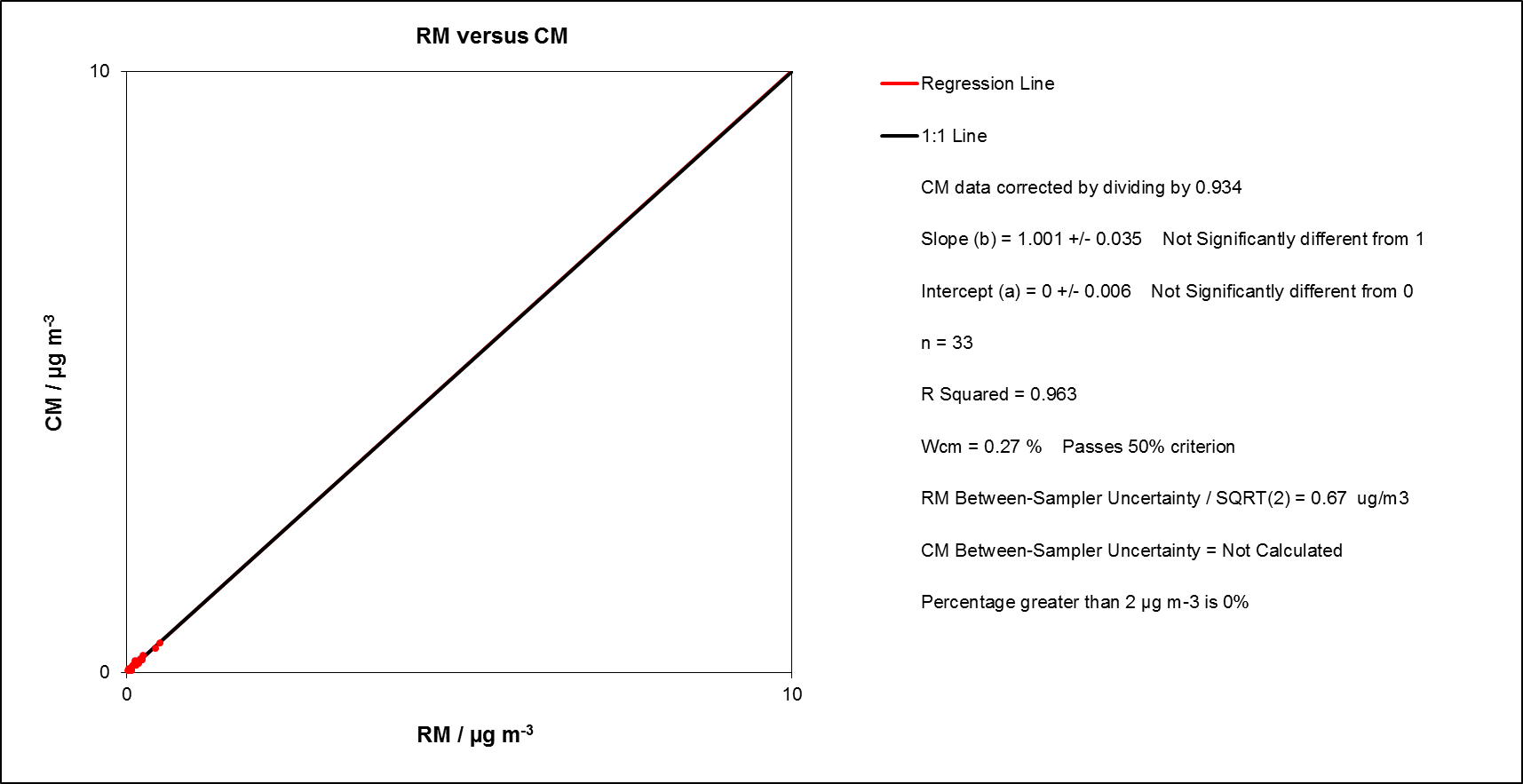


Figure S45, Mg^2+^, AIM (PM_10_) vs RM (PM_10_), North Kensington, UK.

Case study of elevated Na^+^ in the AIM


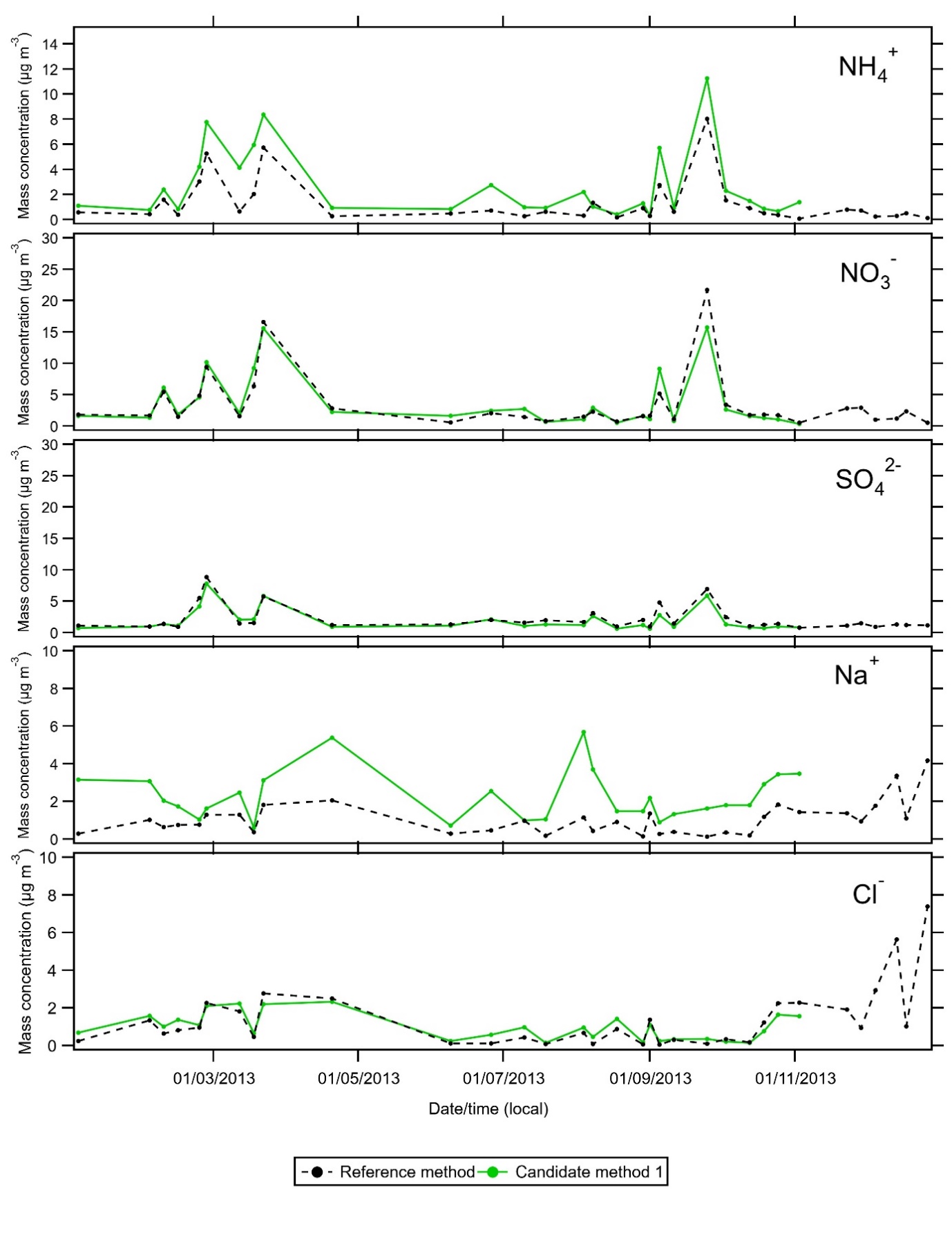


Figure S46 Time series of reference method (PM_10_) and candidate method (AIM) of PM_10_ major ions at North Kensington, London


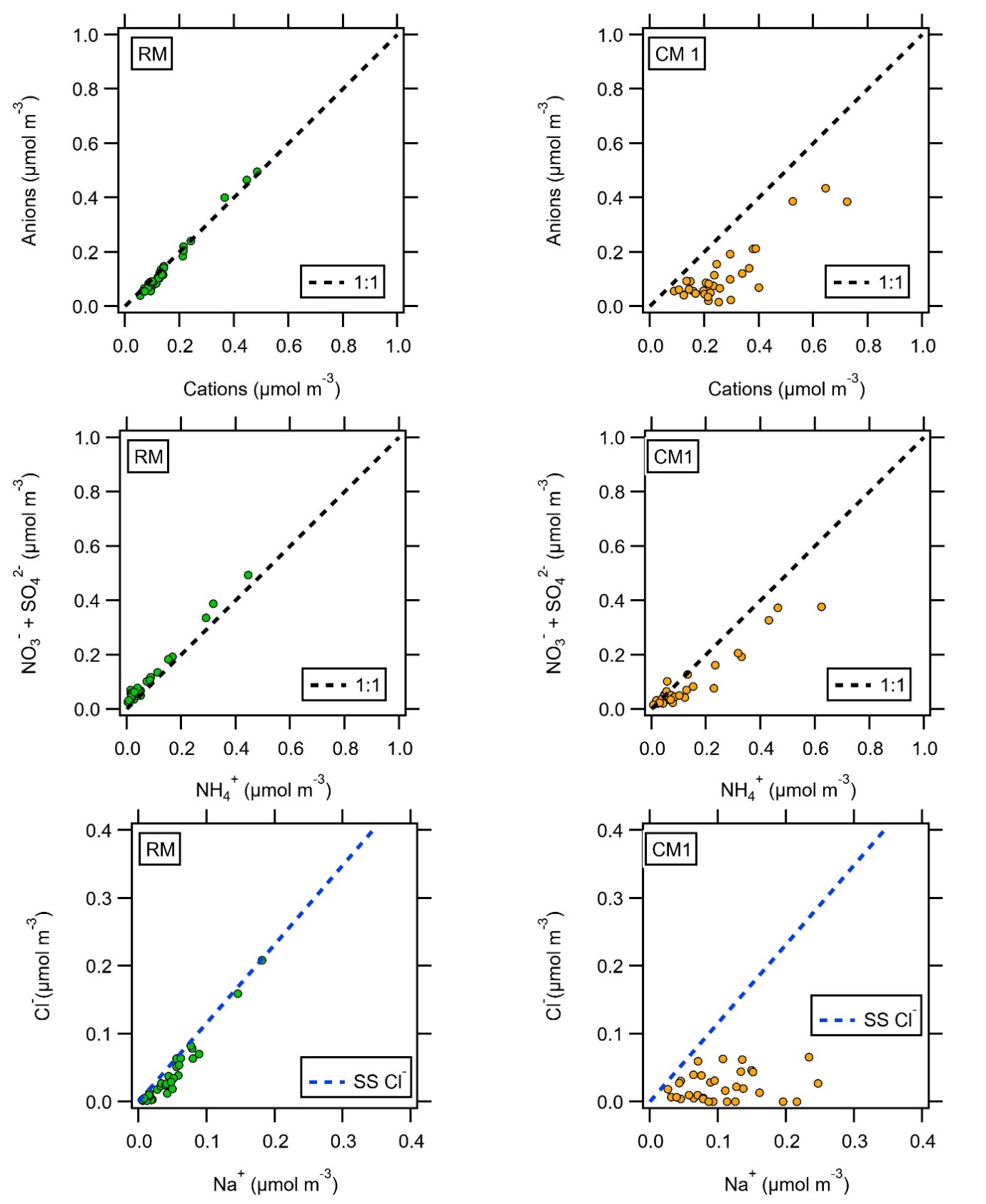


Figure S47 Ion balance of the reference method (RM, PM_10_) and candidate method (CM1, AIM) of PM_10_ at North Kensington, London from January to December 2013. Sea Salt chloride (SS Cl^-^) = calculated concentration of Cl^-^ found in sea salt based on Na^+^ concentration.

# References

1. GDE. *Guide to demonstration of equivalence of ambient air monitoring methods*. (2010).

2. C E N. *ENV 13005 Guide to the expression of uncertainty in measurement*. (1999).

3. Green, D. C., Fuller, G. W. & Baker, T. Development and validation of the volatile correction model for PM10 – An empirical method for adjusting TEOM measurements for their loss of volatile particulate matter. *Atmos. Environ.* **43,** 2132–2141 (2009).
